# Charge Transport Across Dynamic Covalent Chemical Bridges

**DOI:** 10.1021/acs.nanolett.2c03288

**Published:** 2022-10-10

**Authors:** Zelin Miao, Timothy Quainoo, Thomas M. Czyszczon-Burton, Nils Rotthowe, Joseph M. Parr, Zhen-Fei Liu, Michael S. Inkpen

**Affiliations:** † Department of Chemistry, University of Southern California, Los Angeles, California 90089, United States; ‡ Department of Chemistry, Wayne State University, Detroit, Michigan 48202, United States

**Keywords:** single-molecule junctions, electronic coupling, dynamic covalent chemistry, ordered polymers, electronegativity

## Abstract

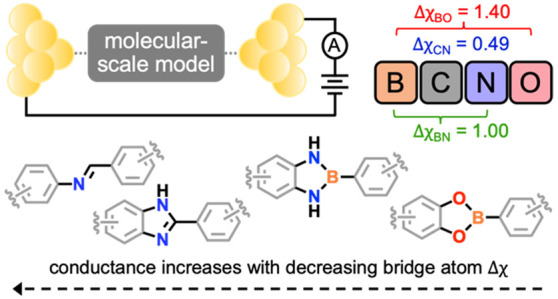

Relationships between chemical structure and conductivity in ordered polymers (OPs) are difficult to probe using bulk samples. We propose that conductance measurements of appropriate molecular-scale models can reveal trends in electronic coupling(s) between repeat units that may help inform OP design. Here, we apply the scanning tunneling microscope-based break-junction (STM-BJ) method to study transport through single-molecules comprising OP-relevant imine, imidazole, diazaborole, and boronate ester dynamic covalent chemical bridges. Notably, solution-stable boron-based compounds dissociate *in situ* unless measured under a rigorously inert glovebox atmosphere. We find that junction conductance negatively correlates with the electronegativity difference between bridge atoms, and corroborative first-principles calculations further reveal a different nodal structure in the transmission eigenchannels of boronate ester junctions. This work reaffirms expectations that highly polarized bridge motifs represent poor choices for the construction of OPs with high through-bond conductivity and underscores the utility of glovebox STM-BJ instrumentation for studies of air-sensitive materials.

Permanently porous, two- and three-dimensional (2D and 3D) ordered polymers (OPs; often referred to as covalent organic or metal–organic frameworks, COFs and MOFs) are a highly modular emergent class of functional materials.^1−4^ Conductive OPs, often comprising pores and channels that permit ion and molecular diffusion, are of broad interest for applications ranging from electrical energy storage,^5,6^ resistive sensing,^7−9^ thermoelectrics,^10^ photovoltaics,^11,12^ or electrocatalysis.^13,14^ They are commonly constructed using dynamic covalent chemical (DCC) groups that link together different building blocks at specific angles to form a desired framework topology.^1,15,16^ Though these and other linking groups (such as metal coordination complexes) have played an important role as fundamental structural elements of many OPs, their impact on bulk material electronic properties is not often clear.^4,17^ For example, the conductivity of a given 2D OP may be dominated by interplane, rather than intrasheet, band transport.^18,19^ Given that conductivity measurements of bulk OP samples, particularly pressed pellets, are influenced by atomic-level defects, grain boundaries, and crystallite anisotropy (among other factors), their intrinsic chemical structure-electronic property relationships remain extremely challenging to elucidate.^18^

Although the underlying mechanisms differ, transport through molecular-scale junctions and OPs are both impacted by the electronic coupling(s) between orbitals or monomer units. Greater coupling between relevant orbitals can increase junction conductance through orbital delocalization^20^ or contribute to a reduced distance-dependent junction conductance decay (β).^21^ It can also improve band dispersion (and so charge carrier mobility) in bulk materials.^22^ For some molecular wires, inverse correlations between β(E) and valence/conduction band dispersion in their hypothetical extended (1D) materials have been identified.^23−25^ Though care must be taken when extrapolating to higher dimensions,^25−29^ we propose that conductance measurements of appropriate molecular models can reveal trends in electronic coupling(s) for OP-relevant chemical structures that may help inform band structure design in extended materials. Such experiments, which directly probe through-bond transport processes, are complementary to conventional approaches such as the use of HOMO–LUMO gaps to probe the extent of conjugation (which in some cases may reflect only the properties of isolated subunits)^26^ or “dimensional reduction” strategies such as the use of Cu, Co, or Ni triphenylene complexes as models to probe bulk spin/electronic coupling interactions.^30−32^

In this establishing study, we use the scanning tunneling microscope-based break-junction (STM-BJ, Figure 1b) method^20,33^ to compare the single-molecule conductance of a series of aurophilic thioether-terminated^34,35^ compounds containing one or two imine^36^ (**2CN** or **2CN-L**), imidazole^37^ (**CN** or **CN-L**), diazaborole^38^ (**BN** or **BN-L**), or boronate ester^39^ (**BO** or **BO-L**) DCC bridge groups (Figures 1c, 2a, and 4a; “**L**” = long and “**2**” = number of bridge atoms). Although the use of imine and boronate ester groups for the construction of OPs is well-established (e.g., Figure 1a),^1^ the lesser utilized imidazole and diazaborole groups are included here to systematically evaluate how conductance is impacted by differences in electronegativity (Δχ) between C, N, B, and O bridge atoms. These comparisons are enabled through use of both air- and glovebox-based STM-BJ setups. While the boron-containing molecules are air-stable in solution, they appear to dissociate *in situ* unless measured under a rigorously inert atmosphere. We observe a clear trend between the decreasing conductance of the intact compounds and Δχ and find that the decay in tunneling conductance with length extension is most rapid in molecules containing boronate ester groups. First-principles calculations using a combination of density functional theory (DFT) and nonequilibrium Green’s functions (NEGF)^40^ corroborate the experimentally observed trends and reveal differences in the nodal structure of the transmission eigenchannels for **BO** and **BO-L** junctions, showing the B–O bonds serve as charge traps that decrease conductance through orbital localization. In support of our underlying premise, the trends observed here for our single-molecule models are in good qualitative agreement with the calculated and experimental properties of relevant OPs, where materials comprising B–O based linkages exhibit flat bands and low charge carrier mobilities relative to those connected by other chemical groups.^41−43^

**Figure 1:**
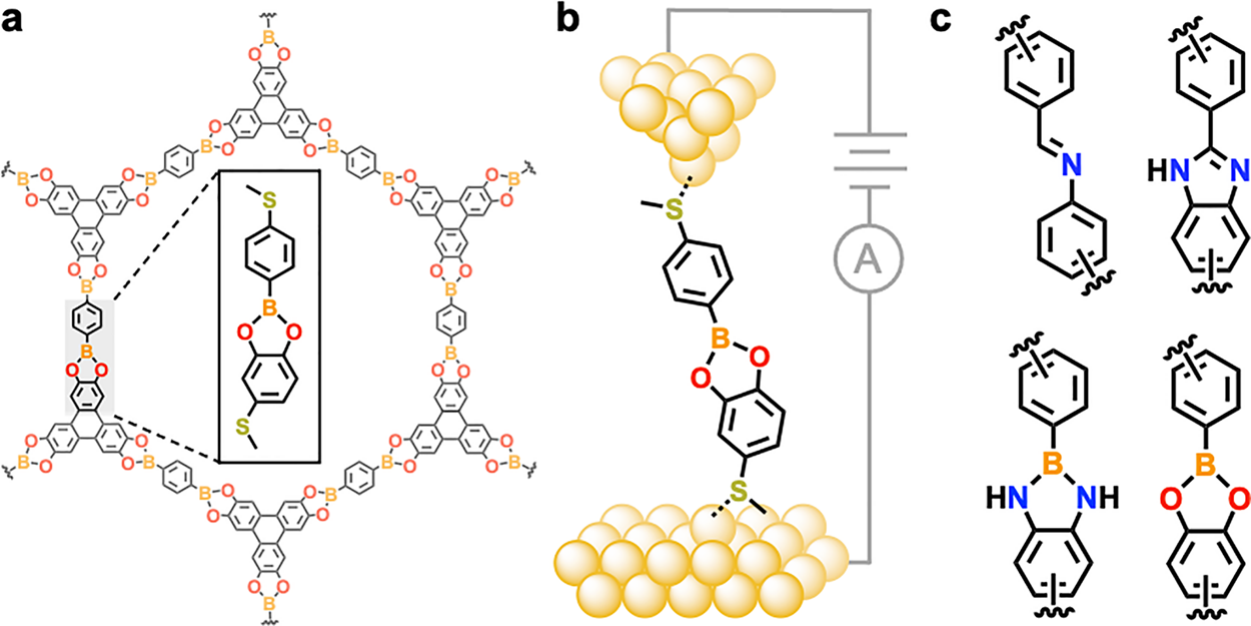
(a) The structure of COF-5, a seminal example of a permanently porous ordered polymer prepared using DCC. In this case, condensation reactions between boronic acids and 1,2-diols (catechols) lead to the formation of an extended molecular framework comprising alternating phenylene and triphenylene groups linked by boronate ester bridges. Inset: the molecular structure of **BO**, a model compound comprising aryl thioether electrode linkers bridged by a single boronate ester group. (b) A schematic representation of a nanoscale molecular junction comprising **BO**. Such junctions are formed using the STM-BJ method, facilitating single-molecule conductance measurements of different model compounds connected between gold electrodes. (c) Molecular structures of imine, imidazole, diazaborole, and boronate ester DCC bridge groups investigated in this study.

**Figure 2:**
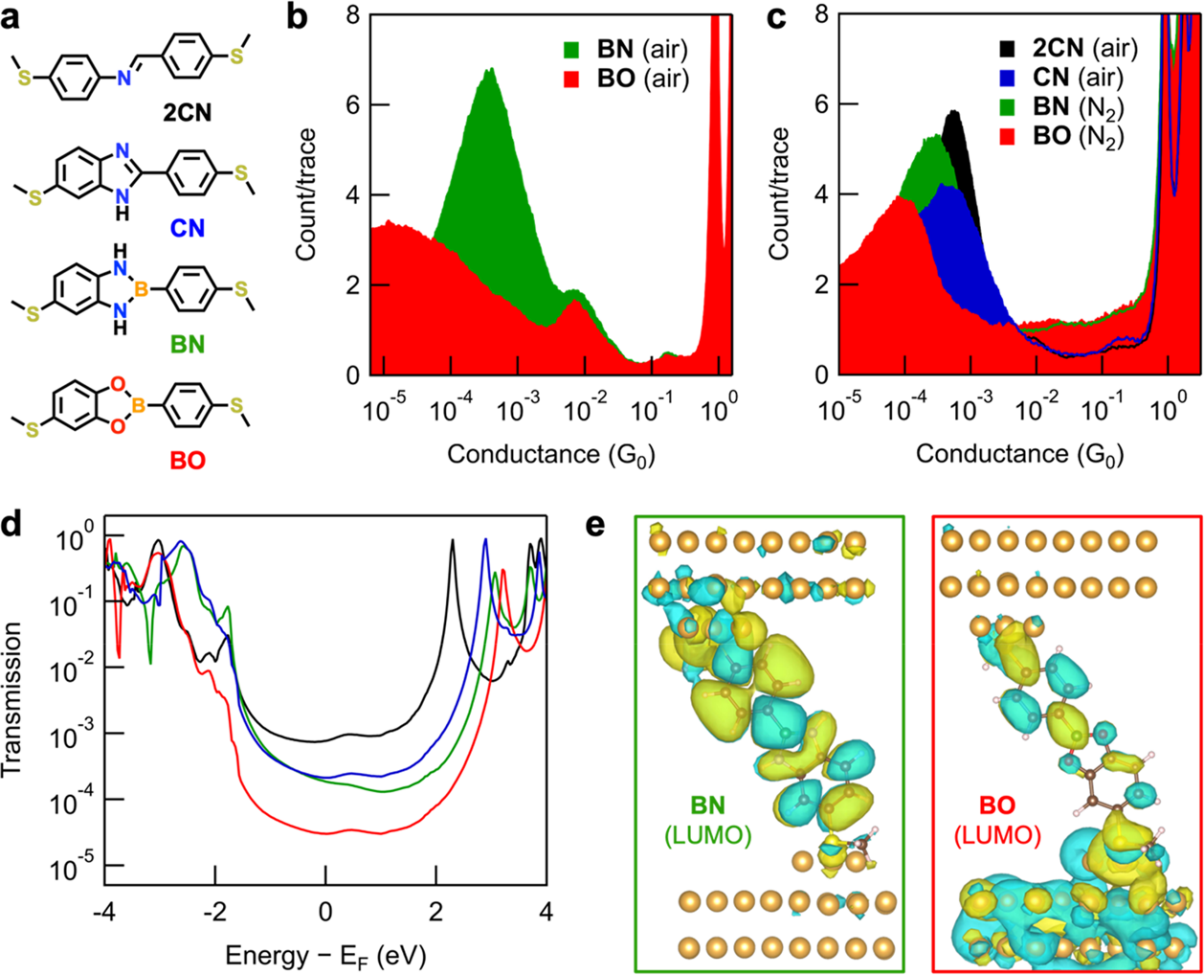
(a) Molecular structures of model compounds containing one DCC bridge group. **CN** has two tautomeric forms but is drawn here with the C=N bond para to the terminal thioether group for simplicity. (b) Overlaid 1D histograms obtained from measurements of **BO** and **BN** in TCB solutions in air (*V*_bias_ = 100 mV, 10,000 traces). We assign the common peak at ∼10^–2^ G_0_ primarily to junctions formed from 4-(methylthio)phenylboronic acid following *in situ* hydrolysis of these compounds (Figure S3), or to covalent Au–C linked junctions following direct transmetalation of the aryl-B group. (c) Overlaid 1D histograms for intact **BN** and **BO** junctions measured under an inert nitrogen atmosphere, and **CN** and **2CN** junctions measured in air (*V*_bias_ = 100 mV, 10,000 traces). For measurements under inert atmosphere, we attribute the higher counts between ∼10^–2^–10° G_0_ to solubilized adventitious impurities or increased interactions between undercoordinated gold atoms and aromatic molecules in the absence of air (see SI for further discussion). (d) Transmission functions for the four molecular junctions obtained from NEGF, using the DFT+∑ approach.^55,56^ The Fermi energy (*E*_F_) of the junction is set to be zero. LUMO resonance peaks for **CN** and **2CN** (between 2 and 3 eV above *E*_F_) approach unity, suggesting symmetric conducting orbitals. Those for **BO** and **BN** (around 3 eV above E_F_) are much smaller than 1, suggesting asymmetric conducting orbitals. (e) Transmission eigenchannels for **BN** and **BO**, evaluated at the LUMO resonance peak. The reduced conductance of **BO**, relative to **BN**, is attributed to its different nodal structure and charge localization around the oxygen atom.

We initially apply the STM-BJ method to measure the conductance of single-molecule junctions comprising one DCC bridge group (Figures 1c and 2a). Synthetic routes to all compounds are described in the SI. In a typical experiment, we repeatedly push a mechanically cut gold STM tip in and out of electrical contact with a gold substrate and measure the conductance as a function of tip–substrate displacement. Each conductance-displacement trace shows step features around integer multiples of the conductance quantum (G_0_ = 2e^2^/h = 7.748 × 10^–5^ S) which are attributed to the formation of single-atom gold point contacts. In the presence of molecules that can bridge the gap, we observe new step features in these traces below 1 G_0_ corresponding to the formation of single-molecule junctions. By compiling thousands of consecutively measured traces into histograms (constructed without data selection), the individual steps add up to form peaks representing the most probable conductance values.

In Figure 2b, we first highlight initial overlaid 1D conductance histograms obtained from 1,2,4-trichlorobenzene (TCB) solutions of **BN** and **BO** prepared and measured in air. Control experiments reveal that the conductance feature observed in each measurement at ∼10^–2^ G_0_ results primarily from the same junctions formed by 4-(methylthio)phenylboronic acid (**MeS-BO**), a common hydrolysis product of both compounds (see the SI for more details). While different boron-containing molecules have been studied using the STM-BJ method,^44−49^ it has recently been reported that arylboron-based compounds can undergo electric field-induced^50−52^ transmetalation reactions at gold surfaces in the presence of oxygen and water to form covalent aryl-Au linked single-molecule junctions.^53^ We therefore propose that the feature observed at ∼10^–2^ G_0_ in our measurements may originate from junctions formed from **MeS-BO** (generated by the hydrolysis of **BN** and **BO**) or by direct aryl-B transmetalation from the intact compounds. Notably, the ^1^H NMR spectra of **BN** and **BO** solutions exposed to air for up to 7 days (Figure S38), or for a **BO** solution stirred in air with Au powder (<10 μm diameter) for ∼2 h (Figure S39),^54^ reveal no signs of significant decomposition. These NMR studies suggest that the dissociation of **BO** and **BN** occurs *in situ* during STM-BJ measurements under ambient conditions, possibly mediated by the applied electric field and/or by reactive undercoordinated gold atoms at the locally roughened tip–substrate interface.^54^ This process is further illustrated in STM-BJ experiments using **BO** solutions prepared under an inert atmosphere then immediately measured in air, showing rapid disappearance of the conductance peak over ∼30 min (Figure S4; junction bias = 100 mV). Notably, we do not observe a clear conductance peak for analogous **BO** measurements in air even when these are performed at a smaller applied electric field (junction bias = 35 mV; close to the minimum we can apply to resolve the conductance peak of **BO** above the instrumental noise floor).

To mitigate the apparent *in situ* dissociation of these arylboron-based compounds, we subsequently use a custom-built STM-BJ setup to form **BN** and **BO** junctions in a N_2_-filled glovebox capable of operating at <1 ppm of H_2_O, <1 ppm of O_2_ (see the SI for further details). In Figure 2c, we plot overlaid 1D conductance histograms for **BO** and **BN** measured in this inert N_2_ atmosphere, as well as for **CN** and **2CN** measured in air. Each shows a single conductance peak as well as the conspicuous absence of features at 10^–2^ G_0_. This indicates that **BO** and **BN** are stable when measured under air-free conditions and that **CN** and **2CN** remain intact during measurements in air (see further discussion in the SI). Despite recent reports of molecular junctions comprising imidazole-based linker groups^57,58^ we see no conductance features attributable to **CN** electrode-binding through the bridge N atoms. This suggests that such features are obscured by the primary conductance peak (attributed to thioether-connected junctions) or that the aryl groups in **CN** serve to sterically inhibit imidazole-electrode binding in these systems. Taken together, the most probable conductance values for the intact junctions, obtained from Gaussian fits to each peak in Figure 2c, are *G*_**2CN**_ = 5.3 × 10^–4^ G_0_ ∼ *G*_**CN**_ = 4.3 × 10^–4^ G_0_ > *G*_**BN**_ = 2.4 × 10^–4^ G_0_ > *G*_**BO**_ = 8.7 × 10^–5^ G_0_.

From these measured conductance values, we recognize an apparent relationship between the decreasing conductance of molecules and the increasing Δχ between bridge group atoms (Δχ_CN_ = 0.49 < Δχ_BN_ = 1.00 < Δχ_BO_ = 1.40 using the Pauling scale). Previous studies have rationalized the low conductance of oligosiloxanes^24^ (Δχ_(Si–O)_ = 1.54) and peptides^59^ (Δχ_(C–N)_ = 0.49), compared to alkanethiols of similar length (Δχ_(C–C)_ = 0), in terms of bond polarization that serves to localize the molecular orbitals responsible for junction transport. The higher conductance of a fluorene-based NPh-bridged wire compared to an O-bridged analogue was also attributed to the higher energy (due to lower χ_(N)_) filled p-orbital of the sp^2^-hybridized N atom that improved alignment and orbital overlap with the carbon-based π-systems.^60^ Of the DCC bridges studied here the B–O bonds in **BO** certainly have the greatest ionic character, resulting from energetically well-separated B and O sp^2^ hybrid atomic orbitals. Although the B–O and B–N bonds of **BO** and **BN** may exhibit partial double bond character (from donation of O/N lone pairs to the empty B p_*z*_ orbital), any π-conjugation is again expected to be reduced for B–O compared to B–N systems in line with their Δχ.^61^

To further explore the potential impact of bond polarization in our DCC-bridged systems, we perform first-principles calculations to determine the electronic transmission of model molecular junctions comprising **2CN**, **CN**, **BN**, and **BO**. Figure 2d shows the transmission functions T(E) for these junctions, calculated using the DFT+∑^55,56^ approach within the NEGF formalism^40^ (see the SI for technical details and additional computational parameters). Reading T(E) at the Fermi level (*E*_F_, set to be zero in Figure 2d), the computed conductance values are *G*_**2CN**_ = 7.7 × 10^–4^ G_0_ > *G*_**CN**_ = 2.1 × 10^–4^ G_0_ > *G*_**BN**_ = 1.9 × 10^–4^ G_0_ > *G*_**BO**_ = 3.0 × 10^–5^ G_0_. These values are in quantitative agreement with experimental results within a factor of 2 in general. From Figure 2d, for every junction, *T*(*E*_F_) is almost flat around *E*_F_, being influenced by both a complex gateway state around −2 eV below *E*_F_ resulting from the hybridization between molecular orbitals and Au *d*-states and a well-defined lowest unoccupied molecular orbital (LUMO) resonance between 2 and 4 eV above E_F_. The gateway states are similar in energy and shape for all junctions; hence we focus on the clear LUMO resonances in our analysis of the transmission differences for each junction. We note that these resonances exhibit a remarkable difference between the junctions with and without boron atoms. While the resonance peaks for **CN** and **2CN** (between 2 and 3 eV above E_F_) approach unity, suggesting symmetric conducting orbitals, the resonance peaks for **BO** and **BN** (around 3 eV above E_F_) are much smaller than 1, suggesting asymmetric conducting orbitals.^62^ This difference indeed highlights the effect of the highly polarized B–O and B–N bonds (compared to the C–N bonds in **CN** and **2CN**) in trapping charges, making the molecular orbital asymmetric, and reducing their conductance in molecular junctions. Furthermore, Figure 2e shows the transmission eigenchannels^63^ of **BN** and **BO** evaluated at their LUMO resonance peaks. The **BO** junction has a different nodal structure than that of **BN** and features charge localization around the oxygen atom (the lobes are not connected with lobes on other atoms), leading to an additional decrease in conductance compared to **BN**.

We next subject **2CN** and **CN** junctions to a closer analysis. Though they each comprise C–N bond motifs, their comparable experimental conductance is perhaps surprising given their different bridge connectivity (Figure 2c). The chemical structure of imidazole, diazaborole, and boronate ester bridges necessitates that one aryl group has bridge connections located at both para and meta positions relative to the thioether substituent in these model compounds. For phenylene bridges, such meta*-*substitution patterns result in destructive interference effects that decrease junction conductance relative to their para*-*substituted analogues.^64−66^ While the two B–O/B-N bonds in **BO**/**BN** are formally identical, the imidazole bridge of **CN** contains distinct C-NH and C=N bonding motifs. Here the N–H proton can be transferred between nitrogen sites with a concurrent shift in the N=C double bond position via a tautomerization reaction,^67^ positioning this either para (**CN*****-*****p**) or meta (**CN*****-*****m**) to the thioether substituent (Figure 3a). Indeed, the ^1^H NMR spectrum of **CN** in DMSO-*d*_6_ shows two distinguishable sets of resonances, indicating both tautomeric forms are present in DMSO solutions at room temperature (Figure S21). However, we only observe one peak in the conductance histograms of **CN** measured in TCB (Figure 2c) or propylene carbonate (PC; Figure S9b).

**Figure 3:**
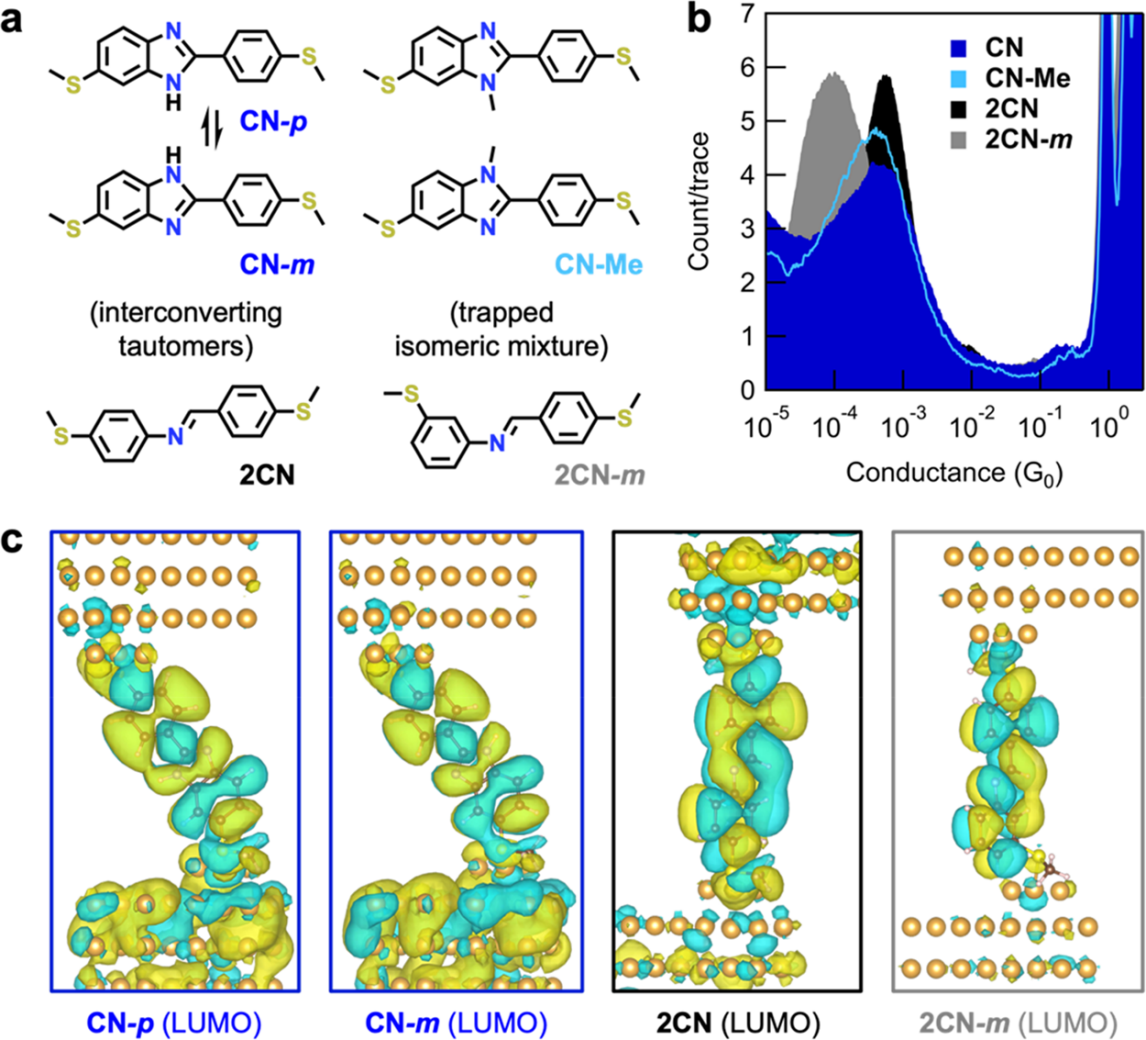
(a) Molecular structures of **CN** (now showing tautomeric equilibrium between **CN*****-*****p** and **CN*****-*****m**), **CN-Me** (an isomeric mixture in ∼1:1 ratio), **2CN**, and **2CN*****-*****m**. (b) Overlaid 1D conductance histograms for **CN**, **CN-Me**, **2CN**, and **2CN*****-*****m** obtained in TCB (*V*_bias_ = 100 mV, 5000–10 000 traces). **CN** and **CN-Me** junctions exhibit a single peak at comparable conductance, showing that the precise position of the C=N bond (and exchange of NH for N-Me) does not significantly change junction conductance. By comparison, **2CN*****-*****m**, a molecule with a meta*-*connected C=N linkage, has a conductance almost ∼5 times lower than **2CN**. Histograms for **CN** and **2CN** are reproduced here from Figure 2c for convenience. (c) Transmission eigenchannels of the two tautomers of **CN**, **2CN**, and **2CN*****-*****m**, evaluated at the LUMO resonance peaks. While the eigenchannels for **CN** tautomers are qualitatively similar, the eigenchannel for **2CN*****-*****m** shows an additional node and charge depletion at the thioether linker relative to **2CN** that leads to a lower conductance value.

To help rationalize these observations, we synthesize and study two additional control compounds: **CN-Me**, an ∼1:1 mixture of each isomeric structure, trapped by replacing the readily exchanged N–H protons with inexchangable methyl groups; and **2CN*****-*****m**, an analogue of **2CN** where the imine group is meta*-*connected to one of the thioether anchor groups (Figure 3a). In Figure 3b, we overlay conductance histograms for **CN**, **CN-Me**, **2CN**, and **2CN*****-*****m**. Remarkably, measurements of the **CN-Me** mixture also show only a single conductance peak. As the peaks in **CN-Me** and **CN** histograms occur at highly comparable conductance values, it is apparent that substitution of NH for NMe, and the formal position of the C=N double bond in **CN-Me** or **CN**, has only a minor impact on junction conductance (close to, or beyond, experimental resolution). In stark contrast, the most probable conductance of **2CN*****-*****m** junctions (9.7 × 10^–5^ G_0_), with only a single C=N double bond meta-connected to the thioether group, is ∼5 times lower than for **2CN** junctions.

Such results are qualitatively supported by our first-principles calculations, where the difference between **2CN** and **2CN-m** is more pronounced than that between **CN-p** and **CN-m**. The transmission functions for these junctions are shown in Figure S12. The sharp difference between the two conformations of **CN** and **2CN** is reflected in the transmission eigenchannel analysis (performed at the LUMO resonance peaks), shown in Figure 3c. While the eigenchannels for the two tautomers of **CN** are qualitatively similar, we observe different charge localization patterns near the linker thioether group between **2CN** and **2CN-m**. In the case of **2CN*****-*****m**, an additional node and charge depletion at the linker thioether group leads to a lower conductance value. Our results are also consistent with predictive chemical models (Figure S8).

To explore how bridge group composition influences the rate of tunneling decay with length extension, we next study a series of analogous compounds comprising two bridge groups (Figure 4a). In Figure 4b, we present overlaid 1D conductance histograms for this longer series of molecules. Their conductance broadly exhibits the same correlation with bridge composition and Δχ as observed for the shorter series, with *G*_**2CN-L**_ = 9.8 × 10^–5^ G_0_ > *G*_**CN-L**_ = 4.6 × 10^–5^ G_0_ > *G*_**BN-L**_ = 2.4 × 10^–5^ G_0_ > *G*_**BO-L**_ = 3.0 × 10^–6^ G_0_. The same trend is predicted from our first-principles NEGF calculations using DFT+∑: *G*_**2CN-L**_ = 1.9 × 10^–4^ G_0_ > *G*_**CN-L**_ = 1.6 × 10^–4^ G_0_ > *G*_**BN-L**_ = 8.6 × 10^–6^ G_0_ > *G*_**BO-L**_ = 3.8 × 10^–6^ G_0_ (for transmission functions see Figure S13). Transmission eigenchannel analysis carried out at the LUMO resonance peaks for the longer molecules (Figure S14) shows that the LUMO resonance is symmetric for all molecules (their structures exhibit C_2_-symmetry about the central aromatic ring, as drawn in Figure 4a). As a result, in contrast to observations for **BN** and **BO** (Figure 2d), T(E) for **BN-L** and **BO-L** now reaches unity between 2 and 3 eV above *E*_F_ (Figure S13). The LUMO resonances for the long boron-containing molecules are noticeably narrower than for those without boron atoms (indicating reduced electronic coupling to the electrodes, which is likely due to reduced intersite coupling within the molecular backbone) and the resonance for **BO-L** shows distinct charge localization near the oxygen atoms, consistent with our findings for the short molecules (Figure 2e).

**Figure 4:**
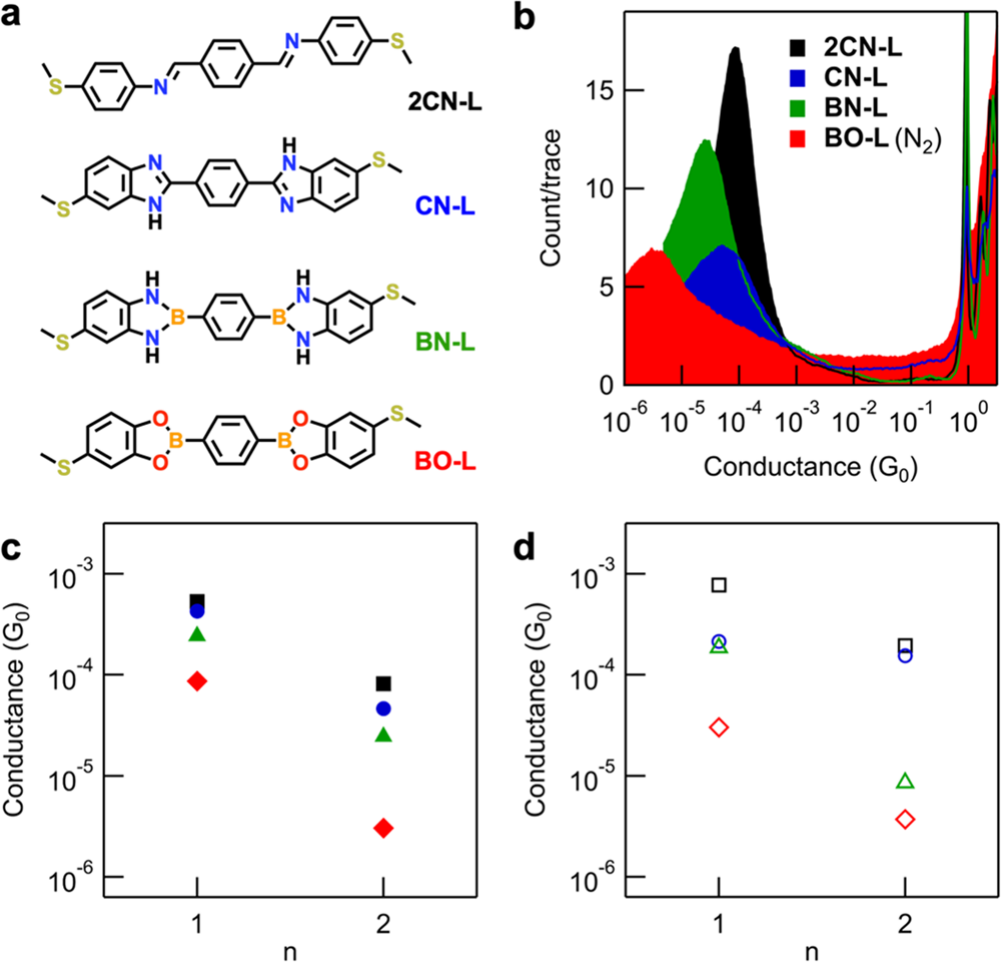
(a) Molecular structures of model compounds containing two DCC bridge groups. **CN-L** has three tautomeric forms but is drawn here with both C=N bonds para to terminal thioether groups for simplicity. (b) Overlaid 1D conductance histograms for junctions comprising two DCC bridge groups (10,000 traces). **BO-L** (red) is measured in TCB under an inert nitrogen atmosphere with *V*_bias_ = 750 mV to lower the instrumental noise floor and better resolve the conductance peak. All other molecules are measured in TCB in air with *V*_bias_ = 100 mV, except for **BN-L** (green) which is measured in PC due to its insolubility in TCB (*V*_tip-bias_ = +100 mV; see the SI for a justification of why these measurements in TCB and PC are comparable). (c) A plot of measured conductance versus number of bridge groups (*n*), showing that junction conductance decays more rapidly with length extension in systems containing boronate esters (red) compared to other bridges. Conductance values were obtained from Gaussian fits to peaks in histograms presented here and in Figure 2c. (d) The same plot as (c), but for calculated conductance from DFT+∑. This illustrates the same qualitative trends.

In Figure 4c,d, respectively, we summarize our findings by plotting the measured and computed conductance against the number of DCC groups. However, these cannot be used to determine true tunneling decay constants (β-values). Because of synthetic challenges, we compare structures without exact oligomeric repeating groups and only isolate and study molecules of two lengths. As an alternative metric for tunneling decay, we instead calculate the ratio of conductance for molecules with 1 and 2 bridge groups for each series (*G*_1/2_). We find the largest measured conductance ratio is *G*_**BO/BO-L**_ = 28.6 (*G*_**2CN/2CN-L**_ = 5.4, *G*_**CN/CN-L**_ = 9.2, *G*_**BN/BN-L**_ = 9.9), showing that addition of a second boronate ester group most significantly impacts the conductance of junctions compared to the other DCC bridge types studied. The conductance ratios for *G*_**CN**/**BO**_ (one bridge group) and *G*_**CN-L**/**BO-L**_ (two bridge groups) junctions are ∼6 and ∼33, respectively, further highlighting the cumulative impact of orbital localizing bridge groups on tunneling over extended distances.

Arguments based on electronegativity and conjugation have similarly been applied to interpret *trends* in the properties of structurally related OPs, for example, in the calculated band dispersions for materials comprising 1,3,5-connected benzene or analogous triazine and boroxine rings.^41^ They may also be used to rationalize why the hole mobility for an imine-bridged porphyrin-based OP was higher than for a structurally similar system with boronate ester linkages (8.1 and 3.0 cm^2^ V^–1^ s^–1^, respectively).^42^ In such bulk materials, decreased π-conjugation (resulting from weaker electronic coupling between aromatic groups) is thought to increase band gaps and reduce band dispersion/charge carrier mobilities.^26,43^ While we re-emphasize here that the absolute values of single-molecule conductance and bulk OP conductivities are governed by distinct charge transport mechanisms and may not be directly related, our study nonetheless helps to reinforce the above interpretations of bulk OP structure–property relationships by using through-bond tunneling transport to probe the impact of DCC bridge groups on trends in the (de)localization of, and so electronic coupling between, relevant molecular orbitals. For comparison to our single-molecule junction studies we also apply a more conventional molecular-scale approach to probe π-conjugation in analogous 1D models,^26^ by analyzing changes in the calculated HOMO–LUMO gaps for different boronate ester and imine oligomers (see Figures S15 and S16). Our results are again in broad qualitative agreement with the proposed electronic coupling trends for these molecular families, showing HOMO–LUMO gaps for imine oligomers are relatively smaller and decrease more rapidly upon length extension.

In conclusion, with the aid of a robust glovebox-based STM-BJ platform we have shown it is possible to resolve, quantify, and rationalize conductance differences for a series of OP inspired compounds comprising 1 or 2 DCC bridge groups of similar connectivity but distinct composition. We find that imine bridges are the most, and polarized boronate ester bridges the least, electronically transparent to tunneling electrons in 1D, reflecting the degree of conducting orbital localization due to bond polarization. Despite fundamental differences in their underlying transport mechanisms, studies of molecular-scale junctions comprising OP-relevant structures can be applied to elucidate trends in local electronic coupling(s). These have the potential to provide new, complementary insights that help in the collective advancement of OP properties and capabilities. Given their demonstrated utility to study intact boronate ester wires, we anticipate that further development of rigorously air-free STM-BJ systems will ultimately provide access to a greatly expanded scope of ambient-pressure single-molecule junction experiments involving air/moisture-sensitive molecular backbones, linkers, and/or electrode materials with the potential to expose a suite of unusual nanoscale chemical and charge transport phenomena.

## Supporting Information Available

Additional synthetic, STM-BJ, and computational details, synthetic procedures, 1D and 2D conductance histograms, transmission calculations, ^1^H and ^13^C{^1^H} NMR spectra for all new compounds (PDF)

## References

[ref1] Yaghi O. M.; Kalmutzki M. J.; Diercks C. S. Introduction to Reticular Chemistry: Metal-Organic Frameworks and Covalent Organic Frameworks, 1st ed.; Wiley Online Books; Wiley-VCH Verlag GmbH & Co. KGaA: Weinheim, Germany, 2019.

[ref2] Geng K.; He T.; Liu R.; Dalapati S.; Tan K. T.; Li Z.; Tao S.; Gong Y.; Jiang Q.; Jiang D. Covalent Organic Frameworks: Design, Synthesis, and Functions. Chem. Rev. 2020, 120, 8814–8933. 10.1021/acs.chemrev.9b00550. 31967791

[ref3] Freund R.; Zaremba O.; Arnauts G.; Ameloot R.; Skorupskii G.; Dincă M.; Bavykina A.; Gascon J.; Ejsmont A.; Goscianska J.; Kalmutzki M.; Lächelt U.; Ploetz E.; Diercks C. S.; Wuttke S. The Current Status of MOF and COF Applications. Angew. Chem., Int. Ed. 2021, 60, 23975–24001. 10.1002/anie.202106259. 33989445

[ref4] Evans A. M.; Strauss M. J.; Corcos A. R.; Hirani Z.; Ji W.; Hamachi L. S.; Aguilar-Enriquez X.; Chavez A. D.; Smith B. J.; Dichtel W. R. Two-Dimensional Polymers and Polymerizations. Chem. Rev. 2022, 122, 442–564. 10.1021/acs.chemrev.0c01184. 34852192

[ref5] Zhang Y.; Riduan S. N.; Wang J. Redox Active Metal- and Covalent Organic Frameworks for Energy Storage: Balancing Porosity and Electrical Conductivity. Chem. - A Eur. J. 2017, 23, 16419–16431. 10.1002/chem.201702919. 28766817

[ref6] Calbo J.; Golomb M. J.; Walsh A. Redox-Active Metal–Organic Frameworks for Energy Conversion and Storage. J. Mater. Chem. A 2019, 7, 16571–16597. 10.1039/C9TA04680A.

[ref7] Kreno L. E.; Leong K.; Farha O. K.; Allendorf M.; Van Duyne R. P.; Hupp J. T. Metal–Organic Framework Materials as Chemical Sensors. Chem. Rev. 2012, 112, 1105–1125. 10.1021/cr200324t. 22070233

[ref8] Meng Z.; Stolz R. M.; Mendecki L.; Mirica K. A. Electrically-Transduced Chemical Sensors Based on Two-Dimensional Nanomaterials. Chem. Rev. 2019, 119, 478–598. 10.1021/acs.chemrev.8b00311. 30604969

[ref9] Meng Z.; Stolz R. M.; Mirica K. A. Two-Dimensional Chemiresistive Covalent Organic Framework with High Intrinsic Conductivity. J. Am. Chem. Soc. 2019, 141, 11929–11937. 10.1021/jacs.9b03441. 31241936

[ref10] Talin A. A.; Jones R. E.; Hopkins P. E. Metal–Organic Frameworks for Thermoelectric Energy-Conversion Applications. MRS Bull. 2016, 41, 877–882. 10.1557/mrs.2016.242.

[ref11] Kaur R.; Kim K.-H.; Paul A. K.; Deep A. Recent Advances in the Photovoltaic Applications of Coordination Polymers and Metal Organic Frameworks. J. Mater. Chem. A 2016, 4, 3991–4002. 10.1039/C5TA09668E.

[ref12] Chueh C.-C.; Chen C.-I.; Su Y.-A.; Konnerth H.; Gu Y.-J.; Kung C.-W.; Wu K. C.-W. Harnessing MOF Materials in Photovoltaic Devices: Recent Advances, Challenges, and Perspectives. J. Mater. Chem. A 2019, 7, 17079–17095. 10.1039/C9TA03595H.

[ref13] Liu J.; Zhu D.; Guo C.; Vasileff A.; Qiao S.-Z. Design Strategies toward Advanced MOF-Derived Electrocatalysts for Energy-Conversion Reactions. Adv. Energy Mater. 2017, 7, 1700518 10.1002/aenm.201700518.

[ref14] Downes C. A.; Marinescu S. C. Electrocatalytic Metal–Organic Frameworks for Energy Applications. ChemSusChem 2017, 10, 4374–4392. 10.1002/cssc.201701420. 28968485

[ref15] Jiang H.; Alezi D.; Eddaoudi M. A Reticular Chemistry Guide for the Design of Periodic Solids. Nat. Rev. Mater. 2021, 6, 466–487. 10.1038/s41578-021-00287-y.

[ref16] Hu J.; Gupta S. K.; Ozdemir J.; Beyzavi M. H. Applications of Dynamic Covalent Chemistry Concept toward Tailored Covalent Organic Framework Nanomaterials: A Review. ACS Appl. Nano Mater. 2020, 3, 6239–6269. 10.1021/acsanm.0c01327. 34327307 PMC8317485

[ref17] Xie L. S.; Skorupskii G.; Dincă M. Electrically Conductive Metal–Organic Frameworks. Chem. Rev. 2020, 120, 8536–8580. 10.1021/acs.chemrev.9b00766. 32275412 PMC7453401

[ref18] Flanders N. C.; Kirschner M. S.; Kim P.; Fauvell T. J.; Evans A. M.; Helweh W.; Spencer A. P.; Schaller R. D.; Dichtel W. R.; Chen L. X. Large Exciton Diffusion Coefficients in Two-Dimensional Covalent Organic Frameworks with Different Domain Sizes Revealed by Ultrafast Exciton Dynamics. J. Am. Chem. Soc. 2020, 142, 14957–14965. 10.1021/jacs.0c05404. 32657123

[ref19] Skorupskii G.; Trump B. A.; Kasel T. W.; Brown C. M.; Hendon C. H.; Dincă M. Efficient and Tunable One-Dimensional Charge Transport in Layered Lanthanide Metal–Organic Frameworks. Nat. Chem. 2020, 12, 131–136. 10.1038/s41557-019-0372-0. 31767997 PMC11060427

[ref20] Venkataraman L.; Klare J. E.; Nuckolls C.; Hybertsen M. S.; Steigerwald M. L. Dependence of Single-Molecule Junction Conductance on Molecular Conformation. Nature 2006, 442, 904–907. 10.1038/nature05037. 16929295

[ref21] Su T. A.; Neupane M.; Steigerwald M. L.; Venkataraman L.; Nuckolls C. Chemical Principles of Single-Molecule Electronics. Nat. Rev. Mater. 2016, 1, 16002 10.1038/natrevmats.2016.2.

[ref22] Hoffmann R. How Chemistry and Physics Meet in the Solid State. Angew. Chem., Int. Ed. 1987, 26, 846–878. 10.1002/anie.198708461.

[ref23] Tomfohr J. K.; Sankey O. F. Complex Band Structure, Decay Lengths, and Fermi Level Alignment in Simple Molecular Electronic Systems. Phys. Rev. B 2002, 65, 1–12. 10.1103/PhysRevB.65.245105.

[ref24] Li H.; Garner M. H.; Su T. A.; Jensen A.; Inkpen M. S.; Steigerwald M. L.; Venkataraman L.; Solomon G. C.; Nuckolls C. Extreme Conductance Suppression in Molecular Siloxanes. J. Am. Chem. Soc. 2017, 139, 10212–10215. 10.1021/jacs.7b05599. 28702995

[ref25] Jensen A.; Strange M.; Smidstrup S.; Stokbro K.; Solomon G. C.; Reuter M. G. Complex Band Structure and Electronic Transmission Eigenchannels. J. Chem. Phys. 2017, 147, 224104 10.1063/1.5016179. 29246062

[ref26] Gutzler R.; Perepichka D. F. Π-Electron Conjugation in Two Dimensions. J. Am. Chem. Soc. 2013, 135, 16585–16594. 10.1021/ja408355p. 24047465

[ref27] Thomas S.; Li H.; Zhong C.; Matsumoto M.; Dichtel W. R.; Bredas J.-L. Electronic Structure of Two-Dimensional π-Conjugated Covalent Organic Frameworks. Chem. Mater. 2019, 31, 3051–3065. 10.1021/acs.chemmater.8b04986.

[ref28] Ni X.; Brédas J. L. Electronic Structure of Zinc-5,10,15,20-Tetraethynylporphyrin: Evolution from the Molecule to a One-Dimensional Chain, a Two-Dimensional Covalent Organic Framework, and a Nanotube. Chem. Mater. 2022, 34, 1334–1341. 10.1021/acs.chemmater.1c04013.

[ref29] Ni X.; Li H.; Liu F.; Brédas J. L. Engineering of Flat Bands and Dirac Bands in Two-Dimensional Covalent Organic Frameworks (COFs): Relationships among Molecular Orbital Symmetry, Lattice Symmetry, and Electronic-Structure Characteristics. Mater. Horizons 2022, 9, 88–98. 10.1039/D1MH00935D. 34866138

[ref30] Yang L.; He X.; Dincâ M. Triphenylene-Bridged Trinuclear Complexes of Cu: Models for Spin Interactions in Two-Dimensional Electrically Conductive Metal-Organic Frameworks. J. Am. Chem. Soc. 2019, 141, 10475–10480. 10.1021/jacs.9b04822. 31180665

[ref31] Intrator J. A.; Orchanian N. M.; Clough A. J.; Haiges R.; Marinescu S. C. Electronically-Coupled Redox Centers in Trimetallic Cobalt Complexes. Dalton Trans. 2022, 51, 5660–5672. 10.1039/D1DT03404A. 35322818

[ref32] Yang L.; Dincă M. Redox Ladder of Ni3 Complexes with Closed-Shell, Mono-, and Diradical Triphenylene Units: Molecular Models for Conductive 2D MOFs. Angew. Chem., Int. Ed. 2021, 60, 23784–23789. 10.1002/anie.202109304. 34472695

[ref33] Xu B.; Tao N. J. Measurement of Single-Molecule Resistance by Repeated Formation of Molecular Junctions. Science 2003, 301, 1221–1223. 10.1126/science.1087481. 12947193

[ref34] Park Y. S.; Whalley A. C.; Kamenetska M.; Steigerwald M. L.; Hybertsen M. S.; Nuckolls C.; Venkataraman L. Contact Chemistry and Single-Molecule Conductance: A Comparison of Phosphines, Methyl Sulfides, and Amines. J. Am. Chem. Soc. 2007, 129, 15768–15769. 10.1021/ja0773857. 18052282

[ref35] Park Y. S.; Widawsky J. R.; Kamenetska M.; Steigerwald M. L.; Hybertsen M. S.; Nuckolls C.; Venkataraman L. Frustrated Rotations in Single-Molecule Junctions. J. Am. Chem. Soc. 2009, 131, 10820–10821. 10.1021/ja903731m. 19722660

[ref36] Uribe-Romo F. J.; Hunt J. R.; Furukawa H.; Klöck C.; O’Keeffe M.; Yaghi O. M. A Crystalline Imine-Linked 3-D Porous Covalent Organic Framework. J. Am. Chem. Soc. 2009, 131, 4570–4571. 10.1021/ja8096256. 19281246

[ref37] Ranjeesh K. C.; Illathvalappil R.; Veer S. D.; Peter J.; Wakchaure V. C.; Goudappagouda; Raj K. V.; Kurungot S.; Babu S. S. Imidazole-Linked Crystalline Two-Dimensional Polymer with Ultrahigh Proton-Conductivity. J. Am. Chem. Soc. 2019, 141, 14950–14954. 10.1021/jacs.9b06080. 31510740

[ref38] Kahveci Z.; Sekizkardes A. K.; Arvapally R. K.; Wilder L.; El-Kaderi H. M. Highly Porous Photoluminescent Diazaborole-Linked Polymers: Synthesis, Characterization, and Application to Selective Gas Adsorption. Polym. Chem. 2017, 8, 2509–2515. 10.1039/C6PY02156E.

[ref39] Côté A. P.; Benin A. I.; Ockwig N. W.; O’Keeffe M.; Matzger A. J.; Yaghi O. M. Porous, Crystalline, Covalent Organic Frameworks. Science 2005, 310, 1166–1170. 10.1126/science.1120411. 16293756

[ref40] Brandbyge M.; Mozos J.-L.; Ordejón P.; Taylor J.; Stokbro K. Density-Functional Method for Nonequilibrium Electron Transport. Phys. Rev. B 2002, 65, 165401 10.1103/PhysRevB.65.165401.

[ref41] Gutzler R. Band-Structure Engineering in Conjugated 2D Polymers. Phys. Chem. Chem. Phys. 2016, 18, 29092–29100. 10.1039/C6CP06101J. 27711626

[ref42] Wan S.; Gándara F.; Asano A.; Furukawa H.; Saeki A.; Dey S. K.; Liao L.; Ambrogio M. W.; Botros Y. Y.; Duan X.; Seki S.; Stoddart J. F.; Yaghi O. M. Covalent Organic Frameworks with High Charge Carrier Mobility. Chem. Mater. 2011, 23, 4094–4097. 10.1021/cm201140r.

[ref43] Raptakis A.; Croy A.; Dianat A.; Gutierrez R.; Cuniberti G. Exploring the Similarity of Single-Layer Covalent Organic Frameworks Using Electronic Structure Calculations. RSC Adv. 2022, 12, 12283–12291. 10.1039/D2RA01007K. 35480357 PMC9027257

[ref44] Olavarría-Contreras I. J.; Etcheverry-Berríos A.; Qian W.; Gutiérrez-Cerón C.; Campos-Olguín A.; Sañudo E. C.; Dulić D.; Ruiz E.; Aliaga-Alcalde N.; Soler M.; Van Der Zant H. S. J. Electric-Field Induced Bistability in Single-Molecule Conductance Measurements for Boron Coordinated Curcuminoid Compounds. Chem. Sci. 2018, 9, 6988–6996. 10.1039/C8SC02337A. 30210774 PMC6124902

[ref45] Liu X.; Li X.; Sangtarash S.; Sadeghi H.; Decurtins S.; Häner R.; Hong W.; Lambert C. J.; Liu S. X. Probing Lewis Acid-Base Interactions in Single-Molecule Junctions. Nanoscale 2018, 10, 18131–18134. 10.1039/C8NR06562D. 30256379

[ref46] Zhao Z.-H.; Wang L.; Li S.; Zhang W.-D.; He G.; Wang D.; Hou S.-M.; Wan L.-J. Single-Molecule Conductance through an Isoelectronic B–N Substituted Phenanthrene Junction. J. Am. Chem. Soc. 2020, 142, 8068–8073. 10.1021/jacs.0c00879. 32321243

[ref47] Zhao Y.-Q.; Lan J.-Q.; Hu C.-E.; Mu Y.; Chen X.-R. Electron Transport of the Nanojunctions of (BN)n (n = 1–4) Linear Chains: A First-Principles Study. ACS Omega 2021, 6, 15727–15736. 10.1021/acsomega.1c00999. 34179616 PMC8223222

[ref48] Baghernejad M.; Van Dyck C.; Bergfield J.; Levine D. R.; Gubicza A.; Tovar J. D.; Calame M.; Broekmann P.; Hong W. Quantum Interference Enhanced Chemical Responsivity in Single-Molecule Dithienoborepin Junctions. Chem. - A Eur. J. 2019, 25, 15141–15146. 10.1002/chem.201903315. 31529793

[ref49] Li H.; Wang R.; Song K.; Wei C.; Hong W.; Zang Y.; Qu D. Substitution Pattern Controlled Charge Transport in BN-Embedded Aromatics-Based Single-Molecule Junctions. Phys. Chem. Chem. Phys. 2022, 24, 2227–2233. 10.1039/D1CP04671C. 35014644

[ref50] Alemani M.; Peters M. V.; Hecht S.; Rieder K. H.; Moresco F.; Grill L. Electric Field-Induced Isomerization of Azobenzene by STM. J. Am. Chem. Soc. 2006, 128, 14446–14447. 10.1021/ja065449s. 17090013

[ref51] Aragonès A. C.; Haworth N. L.; Darwish N.; Ciampi S.; Bloomfield N. J.; Wallace G. G.; Diez-Perez I.; Coote M. L. Electrostatic Catalysis of a Diels–Alder Reaction. Nature 2016, 531, 88–91. 10.1038/nature16989. 26935697

[ref52] Zang Y.; Zou Q.; Fu T.; Ng F.; Fowler B.; Yang J.; Li H.; Steigerwald M. L.; Nuckolls C.; Venkataraman L. Directing Isomerization Reactions of Cumulenes with Electric Fields. Nat. Commun. 2019, 10, 4482 10.1038/s41467-019-12487-w. 31578333 PMC6775130

[ref53] Li Y.; Zhao C.; Wang R.; Tang A.; Hong W.; Qu D.; Tian H.; Li H. In Situ Monitoring of Transmetallation in Electric Potential-Promoted Oxidative Coupling in a Single-Molecule Junction. CCS Chem. 2022, 1–9.

[ref54] Stone I. B.; Starr R. L.; Hoffmann N.; Wang X.; Evans A. M.; Nuckolls C.; Lambert T. H.; Steigerwald M. L.; Berkelbach T. C.; Roy X.; Venkataraman L. Interfacial Electric Fields Catalyze Ullmann Coupling Reactions on Gold Surfaces. Chem. Sci. 2022, 20–24. 10.1039/d2sc03780g PMC9491086 36320717

[ref55] Quek S. Y.; Venkataraman L.; Choi H. J.; Louie S. G.; Hybertsen M. S.; Neaton J. B. Amine–Gold Linked Single-Molecule Circuits: Experiment and Theory. Nano Lett. 2007, 7, 3477–3482. 10.1021/nl072058i. 17900162

[ref56] Neaton J. B.; Hybertsen M. S.; Louie S. G. Renormalization of Molecular Electronic Levels at Metal-Molecule Interfaces. Phys. Rev. Lett. 2006, 97, 216405 10.1103/PhysRevLett.97.216405. 17155759

[ref57] Fu T.; Smith S.; Camarasa-Gómez M.; Yu X.; Xue J.; Nuckolls C.; Evers F.; Venkataraman L.; Wei S. Enhanced Coupling through π-Stacking in Imidazole-Based Molecular Junctions. Chem. Sci. 2019, 10, 9998–10002. 10.1039/C9SC03760H. 32055356 PMC6979055

[ref58] Pan X.; Lawson B.; Rustad A.; Kamenetska M. PH-Activated Single Molecule Conductance and Binding Mechanism of Imidazole on Gold. Nano Lett. 2020, 20, 4687–4692. 10.1021/acs.nanolett.0c01710. 32364746

[ref59] Brisendine J. M.; Refaely-Abramson S.; Liu Z.-F.; Cui J.; Ng F.; Neaton J. B.; Koder R. L.; Venkataraman L. Probing Charge Transport through Peptide Bonds. J. Phys. Chem. Lett. 2018, 9, 763–767. 10.1021/acs.jpclett.8b00176. 29376375 PMC6420303

[ref60] Klausen R. S.; Widawsky J. R.; Su T. A.; Li H.; Chen Q.; Steigerwald M. L.; Venkataraman L.; Nuckolls C. Evaluating Atomic Components in Fluorene Wires. Chem. Sci. 2014, 5, 1561–1564. 10.1039/c4sc00064a.

[ref61] Dewar M. J. S.; Kubba V. P.; Pettit R. New Heteroaromatic Compounds. Part II. Boron Compounds Isconjugate with Indole, 2 : 3-Benzofuran, and Thionaphthen. J. Chem. Soc. 1958, 3076–3079. 10.1039/jr9580003076.

[ref62] Liu Z. F.; Neaton J. B. Voltage Dependence of Molecule-Electrode Coupling in Biased Molecular Junctions. J. Phys. Chem. C 2017, 121, 21136–21144. 10.1021/acs.jpcc.7b05567.

[ref63] Paulsson M.; Brandbyge M. Transmission Eigenchannels from Nonequilibrium Green’s Functions. Phys. Rev. B 2007, 76, 115117 10.1103/PhysRevB.76.115117.

[ref64] Sautet P.; Joachim C. Electronic Interference Produced by a Benzene Embedded in a Polyacetylene Chain. Chem. Phys. Lett. 1988, 153, 511–516. 10.1016/0009-2614(88)85252-7.

[ref65] Mayor M.; Weber H. B.; Reichert J.; Elbing M.; von Hanisch C.; Beckmann D.; Fisher M. Electric Current through a Molecular Rod - Relevance of the Position of the Anchor Groups. Angew. Chem., Int. Ed. Engl. 2003, 42, 5834–5838. 10.1002/anie.200352179. 14673912

[ref66] Solomon G. C.; Herrmann C.; Hansen T.; Mujica V.; Ratner M. A. Exploring Local Currents in Molecular Junctions. Nat. Chem. 2010, 2, 223–228. 10.1038/nchem.546. 21124481

[ref67] Khristich B. I. Tautomerism in a Number of Asymmetrical Imidazole Systems. Chem. Heterocycl. Compd. 1970, 6, 1572–1575. 10.1007/BF00522585.

