# Supplementary material for: Charge Transport Across Dynamic Covalent Chemical Bridges
Source: Nano Lett. 2022 Oct 10;22(20):8331–8. doi: 10.1021/acs.nanolett.2c03288 (PMC9614958; doi:10.1021/acs.nanolett.2c03288)
Supplement: Supplementary file 1 — nl2c03288_si_001.pdf [file nl2c03288_si_001.pdf]

Supplementary Information

Charge transport across dynamic covalent chemical bridges

Zelin Miao,[a] Timothy Quainoo,[b] Thomas M. Czyszczon-Burton,[a] Nils Rotthowe,[a] Joseph M. Parr,[a] Zhen-Fei Liu,[b]* and Michael S. Inkpen[a]*

[a] *Department of Chemistry, University of Southern California, Los Angeles, California 90089, United States*
[b] *Department of Chemistry, Wayne State University, Detroit, Michigan 48202, United States*

E-mail: zfliu@wayne.edu, inkpen@usc.edu

Contents

1. Experimental and Computational Methods S2

2. Synthetic Procedures S8

3. Additional Conductance Data S16

4. Additional Computational Data S25

5. NMR Spectra S30

6. References S43

1. Experimental and Computational Methods

Synthesis and Characterization

All manipulations were carried out in oven-dried glassware under a nitrogen atmosphere using standard Schlenk line techniques. No special precautions were taken to exclude air or moisture during workup unless otherwise stated. Tetrahydrofuran (THF) and dichloromethane (CH_2Cl_2) were sparged with nitrogen and dried using a two-column solvent purification system packed with alumina (Pure Process Technologies, Nashua, NH, USA). *N,N*-Dimethylformamide (DMF) was purified by vacuum distillation, dried over 3Å molecular sieves,[1] and stored under nitrogen. Molecular sieves were activated by heating for ≥3.5 h at 350°C in a muffle furnace (Thermolyne, Thermo Scientific, Asheville, NC, USA), and stored in a desiccator until use. Other reaction solvents (sparged with nitrogen prior to use) and chemical regents were commercially available and used without further purification. Deuterated solvents were purchased from Cambridge Isotope Laboratories, Inc., Cambridge Isotope Laboratories, Tewksbury, MA USA. Flash chromatography was performed using a Pure C-850 FlashPrep automated chromatography system and FlashPure EcoFlex flash cartridges (silica, irregular 40-63 μm particle size, 55-75 Å pore size; BUCHI Corporation, New Castle, DE, USA), or by hand using Alfa Aesar silica gel 60 (215-400 mesh). Reaction yields are unoptimized.

^{1}H and $^{13}C\{^{1}H\}$ NMR spectra were recorded at room temperature on Varian VNMRS 500 (500 MHz), 400MR (400 MHz), VNMRS 600 (600 MHz), or Mercury 400 (400 MHz) NMR spectrometers. ^{1}H NMR data recorded in $CDCl_3$, DMSO-d_6, and toluene-d_8 is referenced to residual internal $C\mathit{H}Cl_3$ (δ 7.26), $(C\mathit{H}D_2)(CD_3)SO$ (δ 2.50), and $C\mathit{H}D_2$-C_6D_5 (δ 2.08) solvent signals.[2] $^{13}C\{^{1}H\}$ NMR data recorded in $CDCl_3$ and DMSO-d_6 is referenced to internal $\mathit{C}DCl_3$ (δ 77.16) and $(\mathit{C}D_3)_2SO$-d_6 (δ 39.52).[2] Mass spectrometry analyses were performed on an Agilent 6545 QTOF mass spectrometer fitted with an atmospheric pressure electrospray ionization source (Dual AJS ESI), or on a Waters Synapt G2-Si (ESI) or Waters GCT Premier (EI) at the Mass Spectrometry Lab, University of Illinois Urbana-Champaign.

Scanning Tunneling Microscope-based Break-Junction (STM-BJ)

Measurements in Air

Scanning tunneling microscope–based break-junction (STM–BJ) measurements were performed using a custom-built STM that has been described previously.[3,4] Hardware was controlled and analyses were performed using custom software (written using IgorPro, Wavemetrics Inc., OR, USA). Experiments were conducted at room temperature under ambient conditions unless otherwise stated. STM tips were prepared from freshly cut Au wire (Ø = 0.25 mm, 99.998%, Alfa Aesar). Substrates for solution measurements were prepared from evaporation of >100 nm Au (99.9985%, Alfa Aesar) at a rate of ~1 Å/s onto mechanically polished AFM/STM steel specimen discs (Ted Pella Inc., CA, USA) with a COVAP Physical Vapor Deposition System (Angstrom Engineering Inc., ON, Canada) used exclusively for metal evaporation. Tip–substrate distances were controlled with sub-angstrom precision using a single-axis preloaded piezoelectric actuator (P-840.1, Physik Instrumente, MA, USA). Tunneling currents were measured using a DLPCA-200 variable gain low noise transimpedance current amplifier (FEMTO Messtechnik GmbH, Berlin, Germany).

Applying a bias between the tip and substrate, conductance was measured as a function of tip–substrate displacement (at 40 kHz acquisition rate) as the tip was repeatedly pushed into the substrate to reach a conductance of >5 G_0 (where $G_0 = 2e^2/h$) and then retracted 5–10 nm (at 20 nm/s) to break the contact. The resulting conductance-distance traces were compiled into 1D conductance histograms (using 100 bins/decade along the conductance axis), or 2D conductance-distance histograms (using 100 bins/decade along the conductance axis and 1000 bins/nm along the displacement axis). Traces in 2D histograms were aligned such that displacement = 0 nm where $G = 0.5\ G_0$. Color scales inset in 2D histograms are in count/1000 traces. Most probable conductance values for molecules studied here were obtained through Gaussian fits to their corresponding conductance peaks in 1D histograms. Directly before starting a solution measurement, substrates were treated with UV/ozone for 20 min (using a NovaScan PSD UV-Ozone Cleaner, Novascan Technologies, Inc., IA, USA) and ≥1,000 traces were collected to check the gold surface was free from contamination. Analytes were studied as solutions (0.1-1 mM) in 1,2,4-trichlorobenzene (TCB; Sigma Aldrich, *ReagentPlus*® or anhydrous, ≥99%), mesitylene (Acros Organics, 99%), tetradecane (TD; Sigma Aldrich, >99%), or propylene carbonate (PC;

Sigma Aldrich, anhydrous, >99.7%). Unless otherwise stated, STM-BJ experiments use normal (non-dried) TCB solutions that are prepared and measured in air. To minimize background (non-tunneling) electrochemical currents, conductance measurements in polar PC solvent were performed using STM tips coated with wax (Apiezon® Vacuum Sealing Wax W, M & I Materials Ltd, Manchester, UK) to leave only the apex area exposed.[5]

Measurements under Inert Atmosphere

Air-free STM-BJ measurements were performed using an ambient temperature and pressure STM-BJ setup identical to the one described above but now housed inside of a customized OMNI-Lab 4-port glovebox (Vacuum Atmospheres Company, Hawthorne, CA, USA; **Figure S1**). Experiments were typically started at single digit or sub-ppm H_2O and O_2 concentrations, as measured by internal glove atmosphere sensors, though the limits of operation have not been systematically probed. O_2 concentrations typically increase to 5-10 ppm during an experiment, as estimated by sensor readings upon re-initiation of air circulation (see below). To the best of our knowledge, previous single-molecule junction experiments conducted under inert atmosphere have utilized environmental chamber, glove bag, or glovebox-based environmental controls that are typically less effective at maintaining low H_2O and O_2 conditions, or these conditions have not been explicitly reported.[6–21]

To isolate our setup from acoustic, electrical, and vibrational noise, the STM head was enclosed in a grounded aluminum box lined with acoustic foam. This environmental isolation box was supported by a conductive aluminum plate which in turn was suspended from the interior ceiling of the glovebox using elastic cords. Neodymium magnets positioned beneath the suspended plate provided additional eddy current dampening. BNC and USB feedthroughs (GS GLOVEBOX Systemtechnik GmbH, Malsch, Germany) were used to connect the STM head to controller and measurement hardware outside the box. Gold substrates were UV-ozone cleaned immediately prior to pumping/sparging them into the glovebox and used for measurements that same day. Solvents for air-free STM-BJ measurements were sparged with nitrogen and dried over 3Å molecular sieves[1] prior to use. To minimize noise during data acquisition, circulation of the glovebox atmosphere was suspended, circulation valves were closed to isolate the catalyst, the vacuum pump was switched off, and the glovebox gloves were tied down to provide additional atmospheric stabilization.

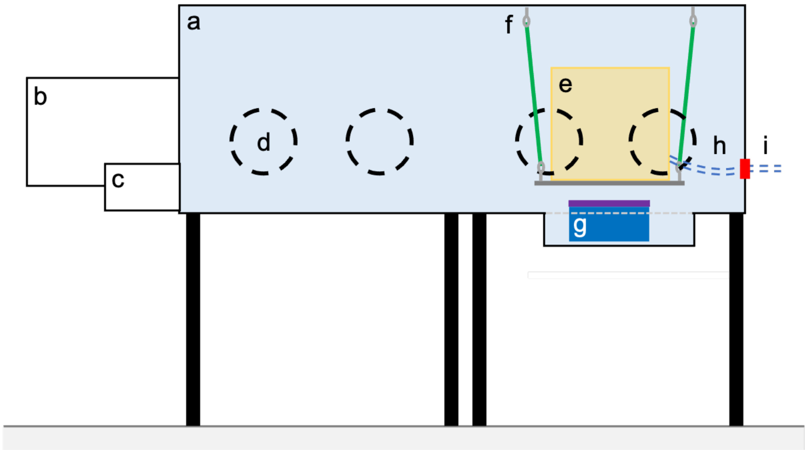

Figure S1. Schematic of glovebox STM-BJ setup: **(a)** The glovebox is capable of maintaining an nitrogen atmosphere with <1 ppm H_2O, <1 ppm O_2 (light blue); **(b)** Large and **(c)** small vacuum/nitrogen antechamber for bringing items in and out of the glovebox; **(d)** Glove ports enable manipulations within the glovebox; **(e)** STM head environmental isolation box; **(f)** Hooks connect a conductive aluminum base plate to the glovebox interior ceiling via elastic cords (green); **(g)** A sunken chamber floor containing an adjustable lab jack (dark blue) allows the STM-BJ head to be raised (providing stabilization to make experimental adjustments) and lowered (for vibrational isolation). The jack is topped with neodymium magnets (purple) to provide eddy current damping at small plate-magnet distances; **(h)** Interior electrical cables (blue dashed lines) connect to (**i**) external cables and hardware via KF-40 feedthroughs (red).

Transmission Calculations using DFT+Σ

The junction structures were constructed by placing seven Au (111) layers (with 4×4 Au atoms on each layer) at each side of the molecule, with the sulfur atom in the molecule binding to an Au trimer.[4,22] During the geometry relaxation, the outer three Au layers on each side were considered as a rigid body, with their relative positions kept as those in the bulk and the force acting on these three Au layers taken as the average force on the atoms in the fourth Au layer. All degrees of freedom of the extended molecule (the inner three Au layers on each side + an Au trimer on each side + the molecule) are fully relaxed, until all forces are below 0.04 eV/Å. The geometry relaxation used the SIESTA package,[23] the Perdew-Burke-Ernzerhof (PBE) functional,[24] a 4×4×1 k-mesh. The Au pseudopotential and basis functions were adapted from prior work[25] and were

chosen to reproduce the work function of Au (111) surface. Single-zeta basis functions were used for Au, and double-zeta basis functions were used for all other elements.

After junction geometry relaxation, the transport calculations were performed within the framework of non-equilibrium Green's function as implemented in TranSIESTA,[26] using the same functional, pseudopotential, basis set, and k-point sampling. After the non-equilibrium density matrix is converged, the coherent transmission functions as a function of energy were computed using the Landauer formula in a post-processing manner,[27] with a 16×16 k-mesh. To correct the quantitative errors of PBE functional in estimating the transport properties, the DFT+ Σ approach[25,28] was applied following the procedure outlined by Liu *et al.*[22] In this approach, the self-energy correction to the molecular orbitals (defined as the eigenvalues in the subblock of the molecular Hamiltonian within the junction) includes two contributions: a gas-phase contribution and a surface polarization, which we discuss separately below.

The gas-phase contribution, Σ_{gp}, is calculated for the isolated molecule. For occupied [unoccupied] levels, this quantity shifts all levels downward [upward] and is defined as the difference between the energy of the highest occupied molecular orbital (HOMO) [the lowest unoccupied molecular orbital (LUMO)] calculated from the OT-RSH functional (see below) and that calculated from the PBE functional. The optimally tuned range-separated hybrid (OT-RSH) functional[29] consists of 20% short-range exact exchange and 100% long-range exact exchange and minimizes the quantity J as a function of γ, the range-separation parameter:

$$J(\gamma) = |\mathrm{IP} + \mathrm{HOMO}(N)| + |\mathrm{EA} + \mathrm{HOMO}(N+1)|.$$

In this equation, HOMO(N) [HOMO(N+1)] is the HOMO of the neutral molecule [the anion with an extra electron]. IP is the ionization potential of the neutral molecule, defined as E(N-1)-E(N). EA is the electron affinity of the molecule, defined as E(N)-E(N+1). Both IP and EA are calculated using total energy differences. As a result, all quantities on the right-hand side of the equation above are γ-dependent, and the OT-RSH HOMO or LUMO energies are those evaluated at the optimal γ that minimizes $J(\gamma)$. In **Table S1**, we report the γ values, HOMO and LUMO energies calculated from both OT-RSH and PBE for the gas phase molecule, as well as the Σ_{gp}. All gas-phase calculations were performed using the Gaussian 16 package[30] with a cc-pVTZ basis.

The surface polarization, Σ_{img}, is modelled using a classical image-charge model,[28] where the image-plane position is set to be at 1.47 Å above the top Au (111) layer (excluding the trimer binding motif). The charge density of the molecule is approximated by a collection of point

charges located at the nuclei with the charge being the Mulliken charge of each atom. In **Table S1**, we also report the Σ_{img} for HOMO and LUMO, as well as the total self-energy correction, $\Sigma = \Sigma_{\mathrm{gp}} + \Sigma_{\mathrm{img}}$. We assume all occupied levels have the same self-energy correction and all unoccupied levels have the same self-energy correction.

Evaluation of HOMO-LUMO Gaps of 1D Oligomers

For a complete picture of how DCC bridge groups modulate the delocalization of frontier orbitals, we probe the π-conjugation in 1D molecules that are analogous to the model compounds studied in the main text. The structures of the 1D molecules are shown in **Figure S15**. We optimize the molecular structures using the B3LYP functional with the 6-31++G** basis, using the Gaussian 16 package. To enable a direct comparison between the 1D molecules studied here and the model compounds studied in the main text, we have constrained all molecular structures to be planar during the geometry optimizations (the free optimization would have resulted non-planar structures for some of the molecules in the ***trans*-2CN** and ***cis*-2CN** series). The trends in HOMO-LUMO gaps are summarized in **Figure S15**, with orbital plots of the longest oligomer in each series presented in **Figure S16** and the raw data of HOMO and LUMO values for all molecules compiled in **Table S2**.

2. Synthetic Procedures

*4-(Methylthio)benzene-1,2-diamine (**MeS-2N**)*

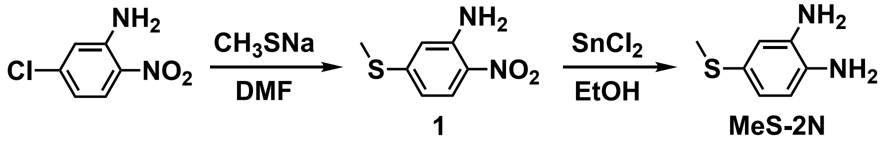

This method was adapted from an analogous procedure.[31] DMF (60 mL) was added to a flask containing 5-chloro-2-nitroaniline (4.491 g, 26.02 mmol) and sodium thiomethoxide (2.98 g, 42.5 mmol). The mixture was heated to 65°C with vigorous stirring for 20 h. After cooling to room temperature, the dark-red mixture was transferred to a separatory funnel and extracted with ethyl acetate (2 × 200 mL). The combined organic extracts were washed with water (3 × 50 mL) and brine (50 mL) before drying them over anhydrous $MgSO_4$. After filtration, solvent was removed by rotary evaporation and the residue purified via flash chromatography (SiO_2; ethyl acetate-hexanes, 0:1→1:1 v/v) to provide **1** as a dark red oil that darkened and solidified overnight (1.813 g, 38%; stored under a nitrogen atmosphere at 5°C). Spectroscopic data was consistent with previous reports.[31] ^{1}H NMR (500 MHz, DMSO-d_6): δ (ppm) 7.86 (d, J = 9.2 Hz, 1H, Ar-H), 7.45 (br s, 2H, -NH_2), 6.79 (d, J = 2.2 Hz, 1H, Ar-H), 6.49 (dd, J = 9.2 and 2.1 Hz, 1H, Ar-H), 2.48 (s, 3H, -CH_3). ^{13}C{^{1}H} NMR (125 MHz, DMSO-d_6): δ (ppm) 148.80, 146.36, 127.55, 125.58, 113.25, 112.12, 13.82 (-SCH$_3$).

1 (1.011 g, 5.488 mmol) and anhydrous $SnCl_2$ (6.314 g, 33.30 mmol) were added to a three-neck round-bottom flask equipped with a reflux condenser. Ethanol (40 mL) was added, and the reaction mixture was heated at reflux for 20 h. After cooling to room temperature, the pH of solution was adjusted to 10-13 by careful addition of aqueous 3 M NaOH. The mixture was then diluted with ethyl acetate (400 mL) and filtered through Celite. The filtrate was washed with brine (50 mL) then dried over $MgSO_4$. After filtration, solvent was removed by rotary evaporation to provide **MeS-2N** as a brown solid (0.805 g, 95%). Spectroscopic data was consistent with previous reports.[31] ^{1}H NMR (500 MHz, DMSO-d_6): δ (ppm) 6.53 (d, J = 2.2 Hz, 1H, Ar-H), 6.45 (d, J = 8.0 Hz, 1H, Ar-H), 6.38 (dd, J = 8.01 and 2.2 Hz, 1H, Ar-H), 4.50 (br s, 4H, -NH_2), 2.29 (s, 3H, -CH_3). ^{13}C{^{1}H} NMR (125 MHz, DMSO-d_6): δ (ppm) 135.70, 133.84, 123.32, 118.32, 115.32, 114.91, 17.83 (-SCH$_3$).

*4-(Methylthio)benzene-1,2-diol (**MeS-2O**)*

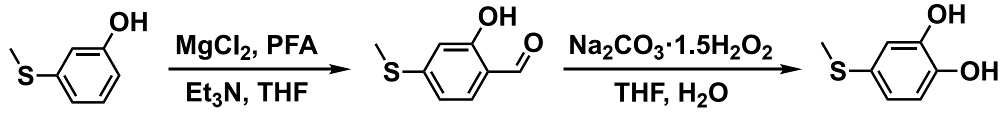

This method was adapted from analogous procedures.[32,33] A 50 mL oven-dried Schlenk flask equipped with a condenser was charged with anhydrous $MgCl_2$ (1.005 g, 10.56 mmol), 3-(methylthio)phenol (1.1 g, 7.8 mmol), triethylamine (3.7 mL, 26.5 mmol), and paraformaldehyde (PFA; 1.478 g, 49.22 mmol). THF (30 mL) was added, and the stirred mixture was heated to reflux for 18 h. After cooling to room temperature, aqueous 2 M HCl (50 mL) was added dropwise to quench the reaction. The mixture was extracted by ethyl acetate (2 × 50 mL), and the combined extracts were dried over $MgSO_4$. After filtration, solvent was removed via rotary evaporation then under high vacuum to provide crude **2** (1.009 g) as a brown solid.

A suspension of crude **2** (0.984 g) and $Na_2CO_3 \cdot 1.5H_2O_2$ (0.936 g, 5.96 mmol) in THF (20 mL) and water (8 mL) was sonicated an in ultrasonic bath for 3 h. The reaction was quenched using acetic acid (2 mL), then solvent was removed under vacuum. The residue was dissolved in methanol, filtered through Celite, and solvent removed by rotary evaporation. The crude product was purified by flash chromatography (SiO_2; ethyl acetate-hexanes, 3:7 v/v) to provide **MeS-2O** as a pale-yellow solid (0.141 g, 12% over two steps). Spectroscopic data was consistent with previous reports.[34] ^{1}H NMR (500 MHz, $CDCl_3$): δ (ppm) 6.87 (s, 1H, Ar-*H*), 6.80 (m, 2H, Ar-*H*), 5.30 (br s, 1H, -O*H*), 5.15 (br s, 1H, -O*H*), 2.43 (s, 3H, -C*H*$_3$). ^{13}C{^{1}H} NMR (125 MHz, $CDCl_3$): δ (ppm) 144.08, 142.03, 129.91, 121.42, 116.09, 115.90, 17.84 (-S*C*H$_3$).

2CN

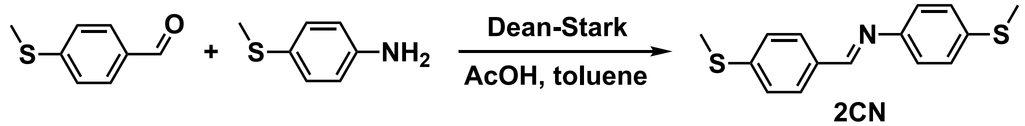

4-(Methylthio)aniline (0.62 mL, 5.0 mmol), toluene (25 mL), 4-(methylthio)benzaldehyde (0.67 mL, 5.0 mmol), and acetic acid (0.05 mL, 0.9 mmol) were added to three-neck round-bottom flask equipped with a Dean-Stark trap. The mixture was heated to reflux overnight with vigorous stirring, then slowly cooled to room temperature. The yellow crystalline solid that precipitated was collected by filtration and washed with hexanes to provide **2CN** (1.158 g, 85%). Spectroscopic data was consistent with previous reports.[35] ^{1}H NMR (600 MHz, $CDCl_3$): δ (ppm) 8.41 (s, 1H, -

N=C*H*-), 7.80 (d, *J* = 8.4 Hz, 2H, Ar-*H*), 7.30 (m, 4H, Ar-*H*), 7.17 (d, *J* = 9.0 Hz, 2H, Ar-*H*), 2.53 (s, 3H, -C*H*3), 2.51 (s, 3H, -C*H*3). ^{1}H NMR (600 MHz, DMSO-d$_6$): δ (ppm) 8.59 (s, 1H, -N=C*H*-), 7.85 (d, *J* = 8.51 Hz, 2H, Ar-*H*), 7.37 (d, *J* = 8.52 Hz, 2H, Ar-*H*), 7.30 (d, *J* = 8.64 Hz, 2H, Ar-*H*), 7.25 (d, *J* = 8.64 Hz, 2H, Ar-*H*), 2.54 (s, 3H, -C*H*3), 2.49 (s, 3H, -C*H*3). ^{13}C{^{1}H} NMR (125 MHz, DMSO-d$_6$): δ (ppm) 159.18, 148.37, 142.79, 135.50, 132.55, 128.98, 126.94, 125.36, 121.79, 15.12 (-S*C*H3), 14.13 (-S*C*H3). HR-MS (ESI+) *m/z*: 274.0733 ([M+H]$^+$ calc. for $C_{15}H_{16}NS_2$: 274.0724).

2CN-m

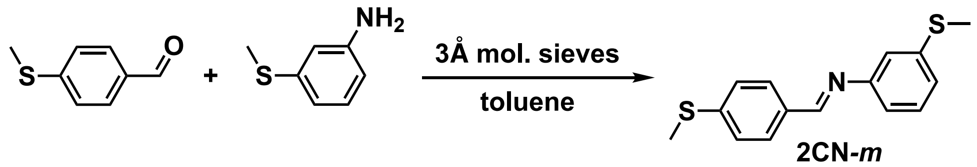

3Å molecular sieves (1.5 g) were added to a solution of 4-(methylthio)benzaldehyde (0.20 mL, 1.5 mmol) and 3-(methylthio)aniline (0.19 mL, 1.5 mmol) in CH_2Cl_2 (10 mL). After stirring overnight at room temperature, the mixture was filtered through Celite and solvent was removed by rotary evaporation. The residue was purified by column chromatography (SiO_2; ethyl acetate-hexanes, 5:1 v/v) to provide **2CN-*m*** as a yellow oil that darkens in air overnight (0.302 g, 74%). ^{1}H NMR (400 MHz, $CDCl_3$): δ (ppm) 8.39 (s, 1H, -N=C*H*-), 7.80 (d, *J* = 8.5 Hz, 2H, Ar-H), 7.30 (m, 3H, Ar-*H*), 7.12 (d, *J* = 7.8 Hz, 1H, Ar-*H*), 7.08 (t, *J* = 2.0 Hz, 1H, Ar-*H*), 6.96 (d, *J* = 8.0 Hz, 1H, Ar-*H*), 2.54 (s, 3H, -C*H*3), 2.52 (s, 3H, -C*H*3). ^{13}C{^{1}H} NMR (100 MHz, $CDCl_3$): δ (ppm) 160.14, 152.82, 143.62, 139.62, 132.83, 129.55, 129.28, 125.82, 123.98, 118.95, 117.54, 15.88, 15.22. HR-MS (ESI+) *m/z*: 274.0741 ([M+H]$^+$ calc. for $C_{15}H_{16}NS_2$: 274.0724).

CN

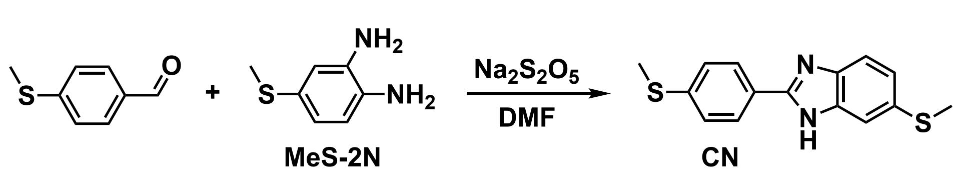

This method was adapted from an analogous procedure.[36] 4-(Methylthio)benzaldehyde (0.13 mL, 0.98 mmol) was added to a stirred solution of **MeS-2N** (0.146 g, 0.947 mmol) and sodium metabisulfite (0.190 g, 1.00 mmol) in DMF (4 mL). The mixture was heated to reflux overnight with vigorous stirring. After cooling to room temperature deionized water (20 mL) was added and the resulting precipitate collected by filtration. Purification by flash chromatography (SiO_2; ethyl

acetate-hexanes, 2:8→1:1 v/v) provided **CN** as a brown solid (0.090 g, 33%). NMR spectra show two sets of partially overlapping resonances for each tautomer (only one tautomer is shown in the reaction scheme for simplicity). ^{1}H NMR (500 MHz, DMSO-d$_6$): δ (ppm) 12.87 (s, 1H, -N*H*-), 12.81 (s, 1H, -N*H*-), 8.08 (m, 4H, Ar-*H*, *both tautomers*), 7.58 (m, 2H, Ar-*H*, *both tautomers*), 7.46-7.40 (m, 5H, Ar-H, *both tautomers*), 7.36 (br s, 1H, Ar-*H*), 7.16 (d, *J* = 8.3 Hz, 1H, Ar-H), 7.13 (d, *J* = 8.5 Hz, 1H, Ar-H), 2.54 (s, 6H, -CH_3, *both tautomers*), 2.53 (s, 3H, -CH_3), 2.51 (s, 3H, -CH_3). ^{13}C{^{1}H} NMR (125 MHz, DMSO-d$_6$): δ (ppm) 151.53, 151.02, 144.67, 142.14, 140.83, 140.71, 135.72, 133.28, 131.38, 130.18, 126.74, 126.29, 125.77, 122.79, 121.72, 119.06, 117.17, 111.63, 109.22, 16.69 (-SCH_3), 16.36 (-SCH_3), 14.27 (-SCH_3, *both tautomers*). HR-MS (ESI+) *m/z*: 287.0679 ($[M+H]^+$ calc. for $C_{15}H_{15}N_2S_2$: 287.0677).

CN-Me

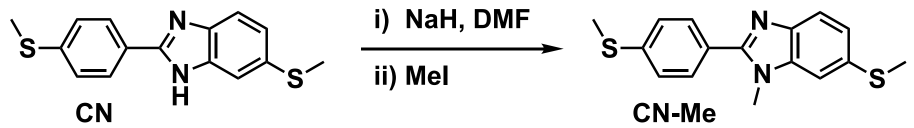

This method was adapted from an analogous procedure.[37] A solution of **CN** (0.174 g, 0.608 mmol) in DMF (10 mL) was cooled to 0°C (water-ice bath) whereby sodium hydride (60% dispersion in mineral oil, 0.052 g, 1.3 mmol) was added portion-wise with stirring. After warming to room temperature over 30 min, methyl iodide (0.2 mL, 3 mmol) was added and the reaction mixture stirred for a further 12 h. The mixture was diluted with ethyl acetate and water, then the organic layer was separated, washed with water, and finally dried over $MgSO_4$. The solvent was removed under reduced pressure to provide the crude product in form of an oil. Crystallization was initiated by the addition of heptane, sonication, and gentle heating to 50°C. The supernatant was decanted off and the residue recrystallized from heptane to provide **CN-Me** as a pale yellow-brown powder (0.178 g, 98%). NMR spectra show two sets of partially overlapping resonances for each isomer (only one isomer is shown in the reaction scheme for simplicity). ^{1}H NMR (400 MHz, DMSO-d$_6$): δ (ppm) 7.79 (m, 4H, Ar-*H*, *both isomers*), 7.57 (m, 4H, Ar-H, *both isomers*), 7.43 (m, 4H, Ar-H, *both isomers*), 7.24 (dd, *J* = 8.5 and 1.8 Hz, 1H, Ar-H), 7.17 (dd, *J* = 8.4 and 1.8 Hz, 1H, Ar-H), 3.87 (s, 3H, NCH_3), 3.86 (s, 3H, NCH_3), 2.56 (s, 3H, -SCH_3), 2.55 (s, 6H, -SCH_3, *both isomers*), 2.52 (s, 3H, -SCH_3). ^{13}C{^{1}H} NMR (100 MHz, DMSO-d$_6$): δ (ppm) 153.16, 152.67, 143.26, 140.76, 140.74, 140.63, 137.44, 135.08, 131.58, 130.70, 129.65, 129.61, 126.15, 126.09, 125.47, 122.52, 121.88, 119.23, 117.16, 111.09, 108.73, 31.87 (m, NCH_3), 16.63 (-SCH_3), 16.38 (-SCH_3),

14.26 (-SCH_3, *both isomers*). HR-MS (ESI+) *m/z*: 301.0841 ($[M+H]^+$ calc. for $C_{16}H_{17}N_2S_2$: 301.0833).

BN

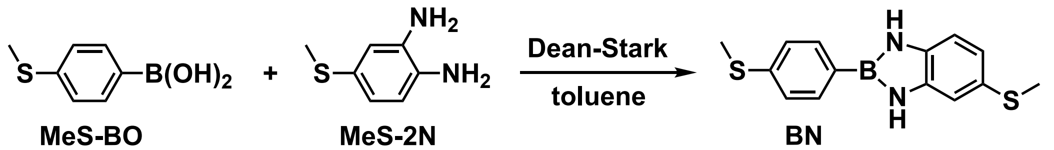

This method was adapted from an analogous procedure.[38] 4-(Methylthio)phenylboronic acid (0.337 g, 2.01 mmol), **MeS-2N** (0.316 g, 2.05 mmol), and toluene (50 mL) were added to a three-neck round-bottom flask equipped with a Dean-Stark trap. The mixture was heated to reflux for 3 h with vigorous stirring, then slowly cooled to room temperature. The pale purple solid that precipitated was collected by filtration and washed with the minimum volume of toluene to provide **BN** (0.122 g, 21%). ^{1}H NMR (600 MHz, DMSO-d_6): δ (ppm) 9.12 (s, 1H, N*H*), 9.11 (s, 1H, N*H*), 7.80 (d, *J* = 8.3 Hz, 2H, Ar-*H*), 7.30 (d, *J* = 8.3 Hz, 2H, Ar-*H*), 7.02 (d, *J* = 1.9 Hz, 1H, Ar-*H*), 6.98 (d, *J* = 7.9 Hz, 1H, Ar-*H*), 6.83 (dd, *J* = 8.1 and 1.9 Hz, 1H), 2.50 (s, 3H, -CH_3), 2.42 (s, 3H, -CH_3). ^{13}C{^{1}H} NMR (150 MHz, DMSO-d_6): δ (ppm) 139.86, 137.94, 136.06, 133.82, 125.40, 125.09, 119.49, 111.43, 111.01, 17.96 (SCH_3), 14.24 (SCH_3), the expected resonance for C_{ipso}-B is absent (**Figure S37**). HR-MS (ESI+) *m/z*: 286.0769 ($[M]^+$ calc. for $C_{14}H_{15}BN_2S_2$: 286.0770).

BO

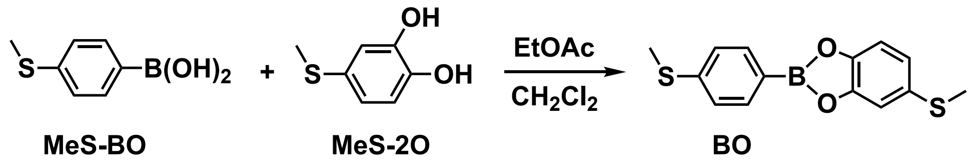

This method was adapted from analogous procedures.[39,40] Ethyl acetate (~6 mL) was added dropwise to a stirred suspension containing **MeS-2O** (0.153 g, 0.979 mmol) and 4-(methylthio)phenylboronic acid (**MeS-BO**, 0.171 g, 1.02 mmol) in CH_2Cl_2 (10 mL) until a homogeneous solution was obtained. The solution was heated to 50°C with vigorous stirring for 3 h, then cooled to room temperature. The resulting mixture was dried over anhydrous $MgSO_4$ and filtered through Celite, then solvent was removed by rotary evaporation to give a brown solid. The crude product was recrystallized from hexanes at (−20 °C) to provide **BO** as a pale-yellow solid (0.032 g, 11%). ^{1}H NMR (500 MHz, $CDCl_3$): δ (ppm) 7.95 (d, *J* = 8.3 Hz, 2H, Ar-*H*), 7.33 (d, *J* = 8.3 Hz, 2H, Ar-*H*), 7.28 (d, *J* = 1.9 Hz, 1H, Ar-*H*), 7.21 (d, *J* = 8.3 Hz, 1H, Ar-*H*), 7.09 (dd, *J* =

8.3 and 2.0 Hz, 1H, Ar-*H*), 2.54 (s, 3H, -*C*H_3), 2.51 (s, 3H, -*C*H_3). $^{13}C\{^1H\}$ NMR (125 MHz, $CDCl_3$): δ (ppm) 149.17, 147.09, 144.74, 135.35, 132.44, 125.31, 122.91, 112.71, 112.67, 17.92 (-S*C*H_3), 14.94 (-S*C*H_3), the expected resonance for C_{ipso}-B is absent (**Figure S37**). HR-MS (EI+) *m/z*: 288.0459 ($[M]^+$ calc. for $C_{14}H_{13}BO_2S_2$: 288.0450).

2CN-L

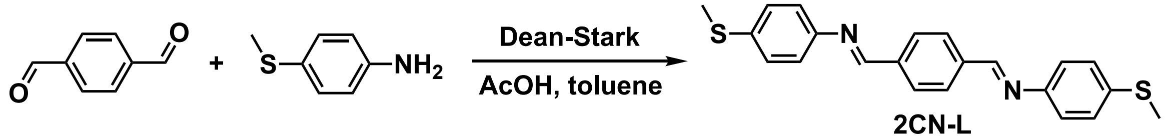

This method was adapted from the one used to prepare **2CN**, described above. Terephthalaldehyde (0.339 g, 2.52 mmol), toluene (25 mL), 4-(methylthio)aniline (0.62 mL, 5.0 mmol), and acetic acid (0.05 mL, 0.9 mmol) were added to a three-neck round-bottom flask fitted with a Dean-Stark trap. The reaction was heated to reflux overnight with vigorous stirring, then slowly cooled to room temperature. The yellow-green crystalline solid that precipitated was collected by filtration and washed with toluene then hexanes to provide **2CN-L** (0.205 g, 22%). ^1H NMR (400 MHz, $CDCl_3$) δ (ppm) 8.53 (s, 2H, -N=C*H*-), 8.00 (s, 4H, Ar-*H*), 7.31 (d, J = 8.6 Hz, 2H, Ar-*H*), 7.22 (d, J = 8.6 Hz, 2H, Ar-*H*), 2.52 (s, 6H, -CH_3). $^{13}C\{^1H\}$ NMR (125 MHz, $CDCl_3$) δ (ppm) 158.80, 148.98, 138.76, 136.69, 129.23, 127.67, 121.79, 16.38 (-S*C*H_3). HR-MS (ESI+) *m/z*: 377.1146 ($[M+H]^+$ calc. for $C_{22}H_{21}N_2S_2$: 377.1146).

CN-L

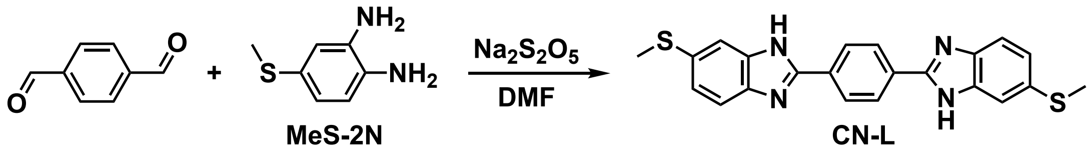

This method was adapted from the one used to prepare **CN**, described above. A mixture of terephthalaldehyde (0.138 g, 1.03 mmol), **MeS-2N** (0.315 g, 2.04 mmol), and sodium metabisulfite (0.581 g, 3.06 mmol) in DMF (4 mL) was heated to reflux overnight with vigorous stirring. After cooling to room temperature, deionized water (20 mL) was added. The resulting dark-green precipitate was collected by filtration and purified by flash chromatography (SiO_2; ethyl acetate-hexanes, 1:1 →0:1 v/v) to provide **CN-L** as a brown solid (0.086 g, 21%). NMR spectra show partially overlapping resonances for three possible tautomers (only one tautomer is shown in the reaction scheme for simplicity). ^1H NMR (600 MHz, DMSO-d_6): δ (ppm) 13.06 (s, 1H, N*H*), 13.00

(s, 1H, N*H*), 8.32 (t, *J* = 3.5 Hz, 4H, Ar-*H*), 7.63 (m, 2H, Ar-*H*), 7.51 (d, *J* = 8.3 Hz, 1H, Ar-*H*), 7.41 (d, *J* = 2.0 Hz, 1H, Ar-*H*), 7.21 (dd, *J* = 8.2 and 1.8 Hz, 1H, Ar-*H*), 7.17 (dd, *J* = 8.4 and 1.8 Hz, 1H, Ar-*H*), 2.55 (s, 3H, -C*H*₃), 2.53 (s, 3H, -C*H*₃). $^{13}C\{^1H\}$ NMR (150 MHz, DMSO-d_6): δ (ppm) 151.06, 150.56, 144.71, 142.15, 135.83, 133.37, 132.00, 130.97, 130.54, 126.85, 123.16, 121.88, 119.35, 117.30, 111.88, 109.21, 16.61 (-S*C*H₃), 16.25 (-S*C*H₃). HR-MS (ESI+) *m/z*: 403.1049 ($[M+H]^+$ calc. for $C_{22}H_{19}N_4S_2$: 403.1051).

BN-L

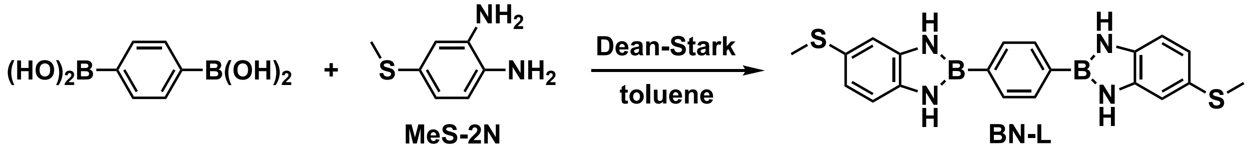

This method was adapted from the one used to prepare **BN**, described above. 1,4-Phenylenediboronic acid (0.168 g, 1.01 mmol), **MeS-2N** (0.306 g, 1.98 mmol), and toluene (50 mL) was added to a three-neck round-bottom flask equipped with a Dean-Stark trap. The mixture was heated to reflux for 3 h with vigorous stirring, then slowly cooled to room temperature. The pale-yellow crystalline solid that precipitated was collected by filtration and washed with methanol to provide **BN-L** (0.013 g, 3%). ^1H NMR (600 MHz, DMSO-d_6): δ (ppm) 9.20 (s, 2H, N*H*), 9.19 (s, 2H, N*H*), 7.92 (s, 4H, Ar-*H*), 7.05 (d, *J* = 1.9 Hz, 2H, Ar-*H*), 7.01 (d, *J* = 7.9 Hz, 2H, Ar-*H*), 6.85 (dd, *J* = 8.0, 1.9 Hz, 2H, Ar-*H*), 2.44 (s, 6H, -C*H*₃). $^{13}C\{^1H\}$ NMR (150 MHz, DMSO-d_6): δ (ppm) 137.95, 136.07, 132.86, 125.51, 119.55, 111.52, 111.15, 17.94 (-S*C*H₃), the expected resonance for C_{ipso}-B is absent (**Figure S37**). HR-MS (ESI+) *m/z*: 402.1345 ($[M]^+$ calc. for $C_{20}H_{20}B_2N_4S_2$: 402.1316).

BO-L

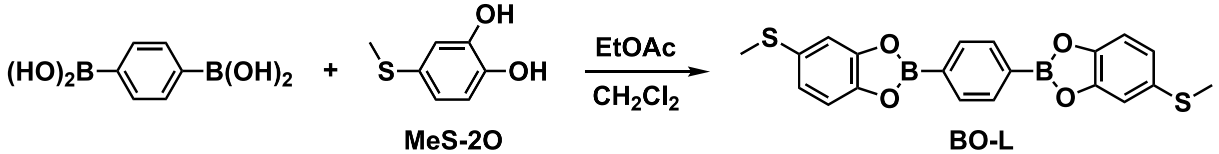

This method was adapted from the one used to prepare **BO**, described above. Ethyl acetate (~14 mL) was added dropwise to a stirred suspension of 1,4-phenylenediboronic acid (0.070 g, 0.42 mmol) and **MeS-2O** (0.132 g, 0.845 mmol) in CH_2Cl_2 (4.25 mL) until a homogeneous solution was obtained. The solution was vigorously stirred for 1 d at room temperature. The resulting mixture was dried over anhydrous $MgSO_4$ and then filtered through Celite. Reduction of the

solution volume by solvent evaporation under vacuum precipitated a white crystalline solid that was collected by filtration and washed with hexanes to provide **BO-L** (0.025 g, 15%). ^{1}H NMR (600 MHz, DMSO-d_6): δ (ppm) 7.35 (s, 4H, Ar-*H*), 6.73 (d, *J* = 2.2 Hz, 2H, Ar-*H*), 6.65 (d, *J* = 7.9 Hz, 2H, Ar-*H*), 6.59 (dd, *J* = 7.9 and 2.0 Hz, 2H, Ar-*H*), 2.38 (s, 6H, -CH_3). ^{13}C{^{1}H} NMR (150 MHz, DMSO-d_6): δ (ppm) 152.30, 150.57, 130.38, 125.45, 119.10, 109.86, 109.00, 17.62 (S*C*H$_3$), the expected resonance for C_{ipso}-B is absent (**Figure S37**). HR-MS (EI+) *m/z*: 406.0688 ([M]$^+$ calc. for $C_{20}H_{16}B_2O_4S_2$: 406.0676).

3. Additional Conductance Data

Stability of Boronate Ester and Diazaborole Junctions

The stability of **BO** and **BN** junctions when measured in the absence of air (**Figure 2c**) is further supported by analysis of 2D conductance-displacement histograms which show their most probable junction lengths are significantly longer (>0.4 nm; **Figure S10c,d**) compared to junctions formed from possible dissociation products (0.1-0.2 nm; **Figure S3c,d**). 4-Methylthioaniline, the common hydrolysis product of **2CN** and **CN**, forms junctions with a conductance around 10^{-2} G_0.[41] Such peak features are absent in conductance histograms for these compounds obtained from STM-BJ measurements in air, suggesting they do not hydrolyze under these conditions (**Figure 2c**).

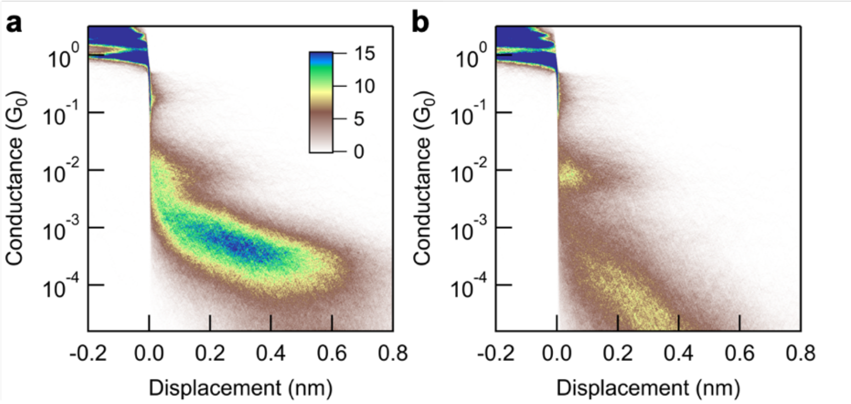

Figure S2. 2D conductance histograms for **(a) BN** and **(b) BO**, measured in air (V_{bias} = 100 mV, 10,000 traces). *Inset:* color scale is in count/1000 traces.

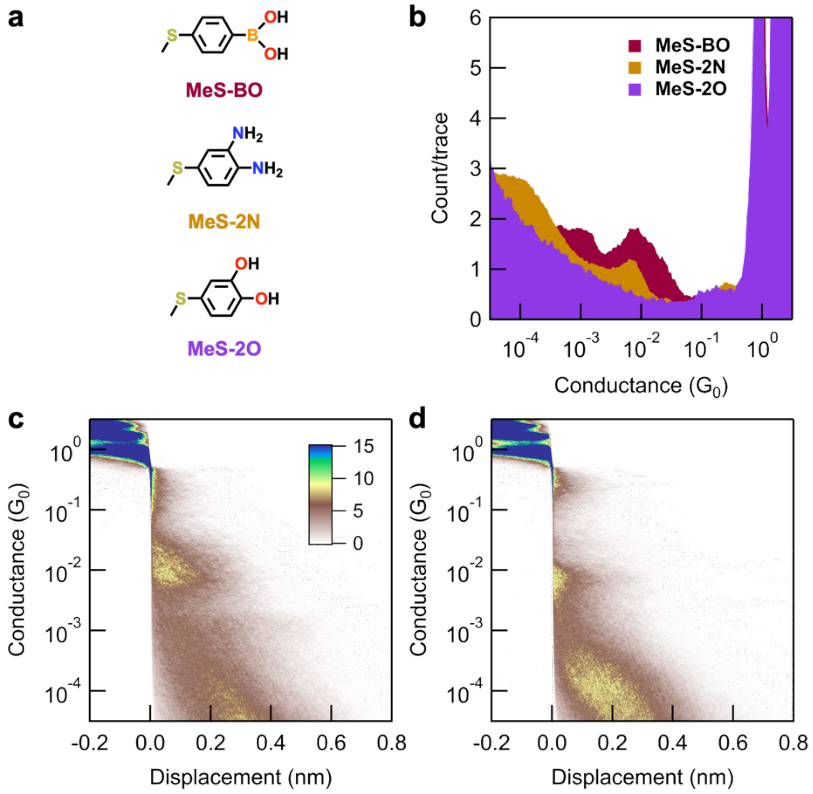

Figure S3. (a) Molecular structures and **(b)** 1D conductance histograms (V_{bias} = 100 mV, 3,500-10,000 traces) for **MeS-BO**, **MeS-2O**, and **MeS-2N**; the hydrolysis products of **BO** and **BN**. **(c)** Corresponding 2D conductance histograms of **MeS-BO** and **(d) MeS-2N**. A common feature is observed at ~10^{-2} G_0 and 1-2 Å displacement in histograms of **MeS-BO**, **MeS-2N**, **BO**, and **BN** (**Figure 2b** and **Figure S2**). We propose that **BO** and **BN** hydrolyze during STM-BJ measurements in air to form **MeS-BO** and **MeS-2N** which we subsequently measure in junctions, or form covalent Au-C linked junctions following direct aryl-B transmetalation.[42]

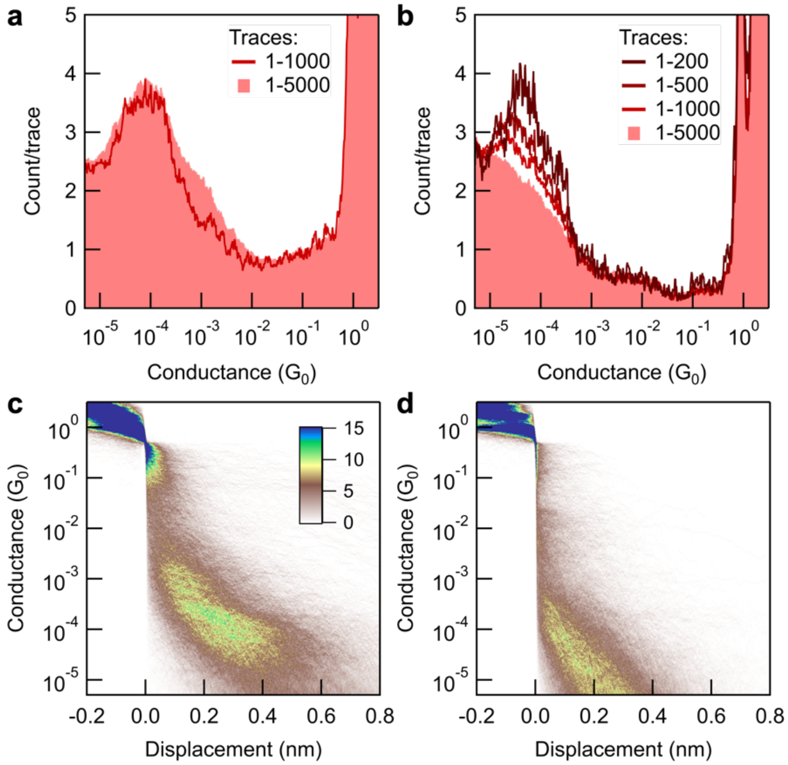

Figure S4. Conductance histograms obtained from the same 1 mM **BO** solution prepared in a glovebox using anhydrous, oxygen-free TCB, measured under an inert atmosphere **(a, c)** or in air **(b, d)**. Lines in overlaid 1D histogram plots (top) are constructed from the first 200-1,000 traces obtained over 5-25 min. Filled 1D histograms (pale red) in overlaid plots (top) and 2D histograms (bottom) are constructed from 5,000 traces obtained over ~2 h (V_{bias} = 100 mV). In **(a, c)**, the solution was measured in the glovebox under inert conditions, showing a persistent well-defined peak at ~10^{-4} G_0. In **(b, d)**, a sample of the same solution was removed from the glovebox and immediately subjected to measurements in air. A similar peak feature is observed in histograms built from the first 200-500 traces, which then rapidly decreases in intensity over ~30 min. Both sets of measurements were performed on the same day.

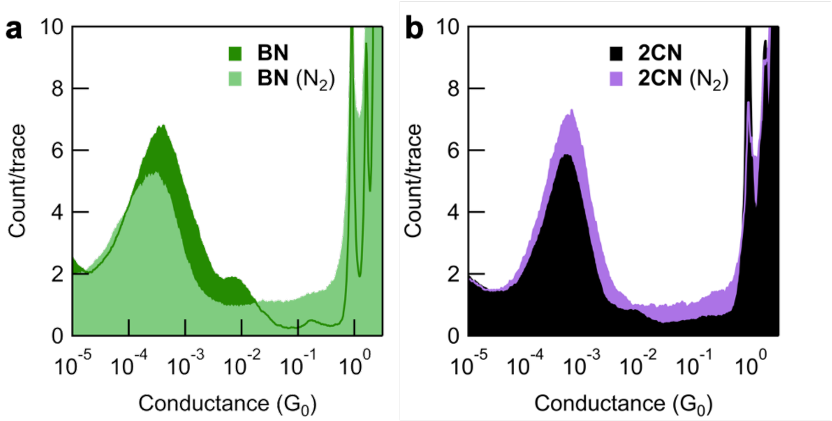

Figure S5. Overlaid 1D conductance histograms for **(a) BN** and **(b) 2CN**, measured in air and in a nitrogen-filled glovebox (V_{bias} = 100 mV, 5,000-10,000 traces). No significant differences are observed between the (primary) conductance peaks when measuring these molecules in air or under an inert atmosphere.

Apparent Noise in Glovebox STM-BJ Conductance Histograms

Beyond the interpretation of molecular conductance trends, we also recognize that the histograms obtained from glovebox-based STM-BJ experiments presented in **Figure 2c** exhibit an apparent higher noise than for analogous measurements in air, as indicated by the increased counts between 10^{-2}-10^{0} G_0 and decreased resolution of atomic point contact features above ~1 G_0 (see also **Figure S6**). While our glovebox STM-BJ setup operates in a more challenging chemical, vibrational, and acoustic environment with the potential to increase measurement noise, we find that these apparent noise features are typically absent in clean gold measurements prior to the addition of solvent (see **Figure S7a-c**, yellow). Glovebox STM-BJ studies in different anhydrous, deoxygenated solvents reveal that the apparent noise features are present when using TCB and mesitylene, but absent with tetradecane (TD; **SI, Figure S7a-c**, brown). These features are not observed for measurements in air using solvent samples taken from the same batches as used in the glovebox measurements, ruling out solvent contamination during drying or deoxygenation. We hypothesize that such "noise" features are attributable to solubilized adventitious impurities (e.g., present on the gold surface or in the atmosphere) or increased interactions between undercoordinated gold atoms and TCB/mesitylene in the absence of air. Notably, the presence of oxygen has been found to increase the length of single atomic gold chains in low temperature break-junction measurements,[43] suggesting that oxygen (and/or water) may serve to stabilize undercoordinated gold atoms during STM-BJ measurements in air.

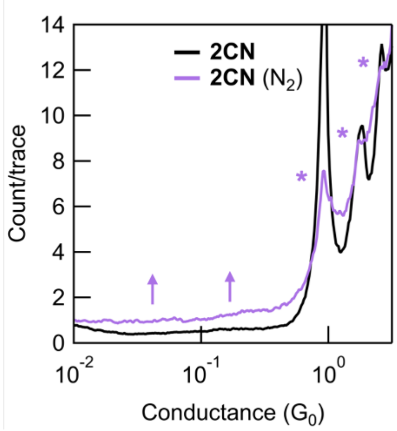

Figure S6. Expanded view of overlaid 1D conductance histograms for TCB solutions of **2CN** measured in air and in a nitrogen-filled glovebox (V_{bias} = 100 mV, 5,000-10,000 traces). We observe increased counts between 10^{-2}-10^{0} G_0 (arrows) and decreased resolution of atomic point contact features above ~1 G_0 (starred peaks).

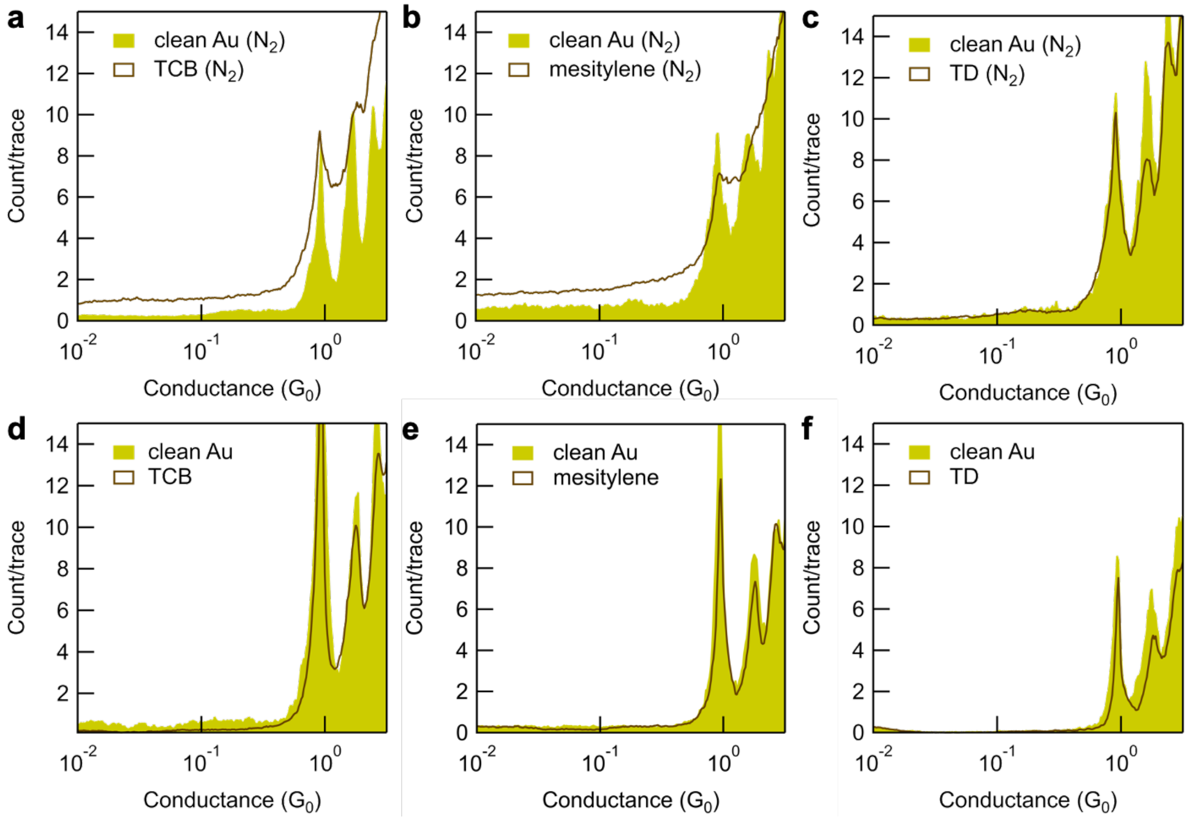

Figure S7. *Top:* Overlaid 1D conductance histograms obtained for clean gold substrates measured *in a nitrogen-filled glovebox* before (yellow; ≥1,000 traces) and after (brown; ≥5,000 traces) addition of **(a)** TCB, **(b)** mesitylene, or **(c)** TD (V_{bias} = 100 mV). *Bottom:* Overlaid 1D conductance histograms obtained for clean gold substrates measured *in air* before (yellow) and after (brown) addition of *the same samples* of **(d)** TCB, **(e)** mesitylene, or **(f)** TD (identical V_{bias} and number of traces as for (a)-(c)). The lack of significant apparent noise features for clean gold measurements measured in the glovebox prior to solvent addition (a-c, dark yellow) shows that the operation of this instrument is not significantly affected by chemical contamination, or vibrational or acoustic noise. For glovebox measurements using TCB or mesitylene we observe increased background counts and decreased resolution of atomic point contact features, relative to measurements of the same gold substrates prior to solvent addition, or to measurements using TD.

Predictions from Chemical Models

Application of the "extended curly arrow rules" recently presented by O'Driscoll and Bryce[44] to **CN-*m*** (where the C=N bond is positioned *meta* to -SMe, **Figure 3a**) suggest that it should exhibit a "*shifted* destructive" quantum interference (SDQI; **Figure S8a**) rather than DQI. Here the electron-donating *para*-connected -NH- substituent (absent in **2CN-*m***) is considered to increase the conductance of this tautomer/isomer relative to that of **2CN-*m*** by moving the transmission

antiresonance away from the Fermi level (E_F). While the predicted result from this model agrees with our findings for **CN-*m***, hybridization between molecular states and gold *d*-states complicates interpretation of the change in transmission in terms of a simple shift in antiresonance position (**Figure S12**). We also note, for completeness, that further arrow pushing allows us to draw a zwitterionic resonance form for **CN-*m*** that places the C=N bond *para* to the -SMe group (**SI, Figure S8b**), resulting in an electronic structure that resembles that of the most important resonance form of **CN-*p***.

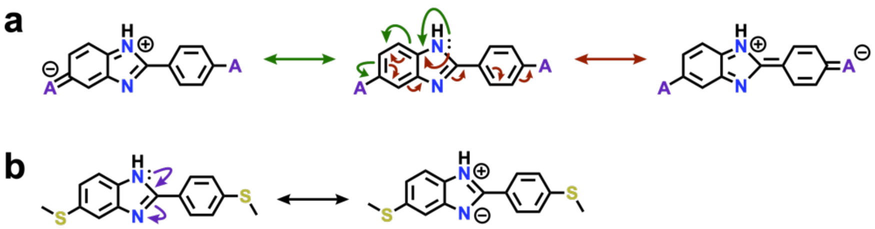

Figure S8. (a) Application of "extended curly arrow rules"[44] to the "**CN-*m*"** tautomer having the C=N bond *meta*-connected to one thioether (-SMe) group (denoted here as A = acceptor). The lone pair of the electron-donating -NH- group positioned *para* to the same -SMe/A substituent can be delocalized through curly arrow electron pushing to both -SMe/A. This indicates that **CN-*m*** should exhibit "shifted destructive" quantum interference (SDQI, transmission antiresonance far from E_F) rather than DQI (transmission antiresonance near to E_F). **(b)** A zwitterionic resonance form for **CN-*m*** that places the C=N bond para to both -SMe groups.

Measurements in a Polar Solvent (Propylene Carbonate)

Control studies in propylene carbonate (PC) show that changes in solvent or bias polarity (at low biases) do not significantly impact the most probable conductance of the molecular junctions studied here (**Figure S9**). These experiments further support our computational studies which show T(E) is almost flat around E_F (little to no current rectification should be observed when opening the bias window asymmetrically in polar solvents;[45] **Figure 2d** and **Figure S13**), and show it is reasonable to directly compare the conductance of **BN-L** in PC with the conductance of other molecules measured in TCB.

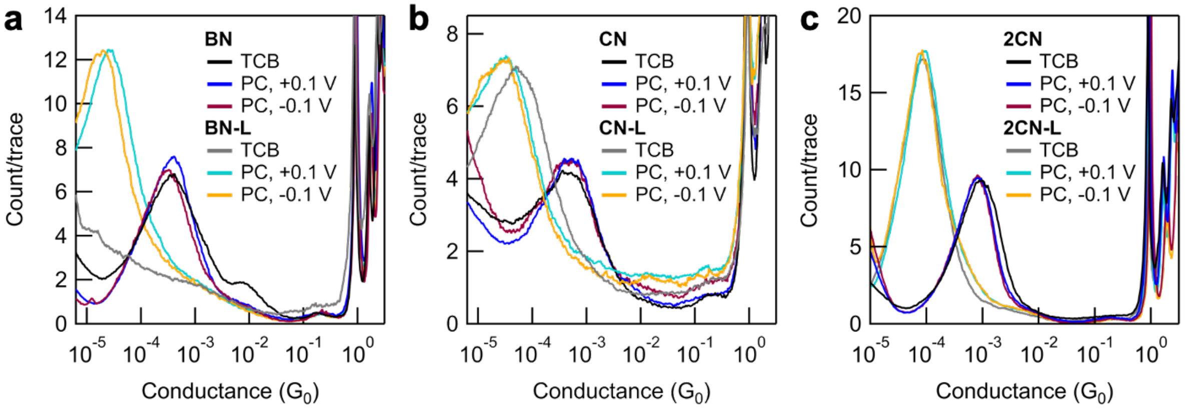

Figure S9. Overlaid 1D histograms for **(a) BN** and **BN-L**, **(b) CN** and **CN-L**, and **(c) 2CN** and **2CN-L**, measured in TCB (V_{bias} = 100 mV) and anhydrous PC in air at different tip bias polarities (3,000-10,000 traces). No significant effect of changing solvent or bias polarity on conductance is observed. Measurement of a saturated TCB solution of **BN-L** (panel a, grey) shows no conductance peak due to the low solubility of this compound in this solvent. No dissociation products (indicated by conductance features at ~10^{-2} G_0) are observed for **BN** or **BN-L** measured in anhydrous PC in air (panel a).

2D Conductance-Displacement Histograms

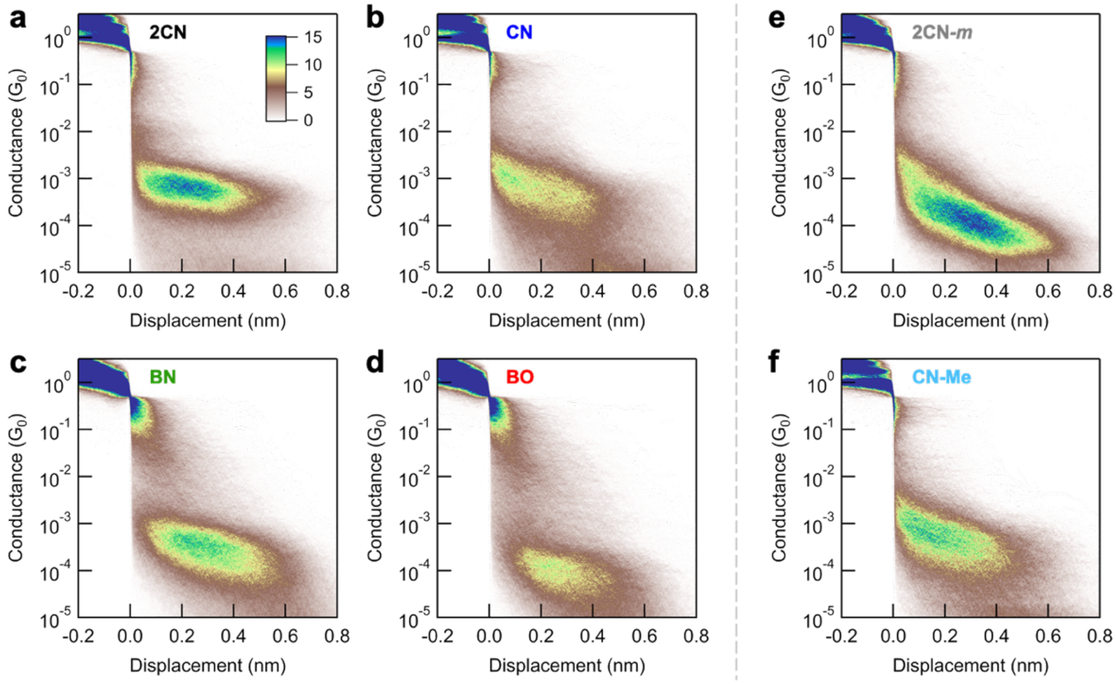

Figure S10. Conductance-displacement 2D histograms for the 1D data shown in **Figure 2c** and **3**. Measurements of **BN** and **BO** are performed in a glovebox in anhydrous TCB.

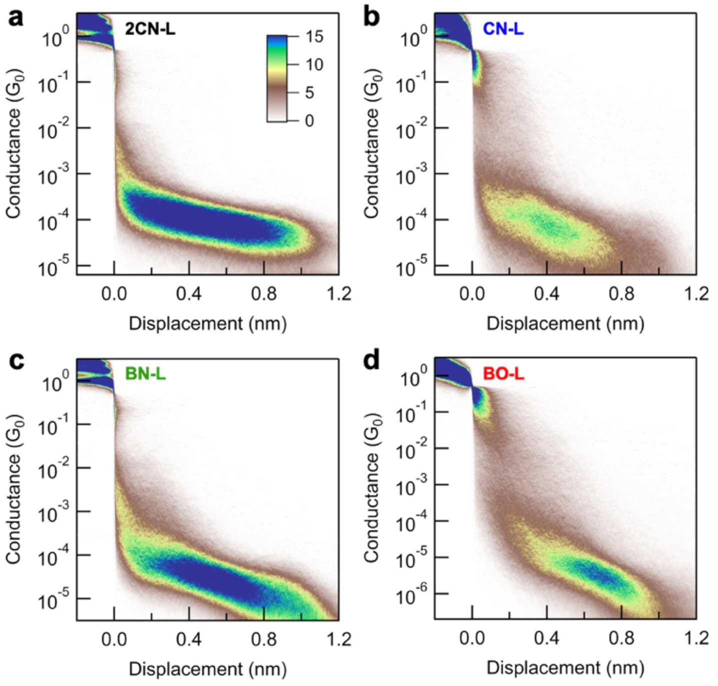

Figure S11. Conductance-displacement 2D histograms for the 1D data shown in **Figure 4**. **BN-L** is measured in anhydrous PC in air, **BO-L** is measured in a glovebox in anhydrous TCB.

4. Additional Computational Data

Table S1: Parameters used and results for self-energy calculations for the molecules. All energies are in eV.

		Self-energy calculation for occupied orbitals					Self-energy calculation for unoccupied orbitals				
Molecule	γ (Bohr^{-1})	HOMO (OT-RSH)	HOMO (PBE)	Σ_{gp}	Σ_{img}	Σ_{HOMO}	LUMO (OT-RSH)	LUMO (PBE)	Σ_{gp}	Σ_{img}	Σ_{LUMO}
**BO**	0.19	−7.62	−4.96	−2.66	1.36	−1.30	−0.04	−2.36	2.32	−0.86	1.46
**BN**	0.17	−6.90	−4.49	−2.41	1.16	−1.25	0.20	−1.96	2.16	−0.92	1.24
**CN**	0.18	−7.33	−4.80	−2.53	1.43	−1.10	−0.36	−2.49	2.13	−0.81	1.32
**2CN**	0.16	−7.39	−5.02	−2.37	1.12	−1.25	−0.86	−2.89	2.03	−0.70	1.33
**BO-L**	0.15	−7.34	−4.96	−2.38	1.08	−1.30	−0.61	−2.63	2.02	−0.53	1.49
**BN-L**	0.15	−6.73	−4.48	−2.25	0.86	−1.39	−0.24	−2.19	1.95	−0.54	1.41
**CN-L**	0.14	−6.81	−4.72	−2.09	0.78	−1.31	−0.95	−2.71	1.76	−0.56	1.20
**2CN-L**	0.14	−7.16	−5.04	−2.12	0.76	−1.36	−1.51	−3.29	1.78	−0.48	1.30

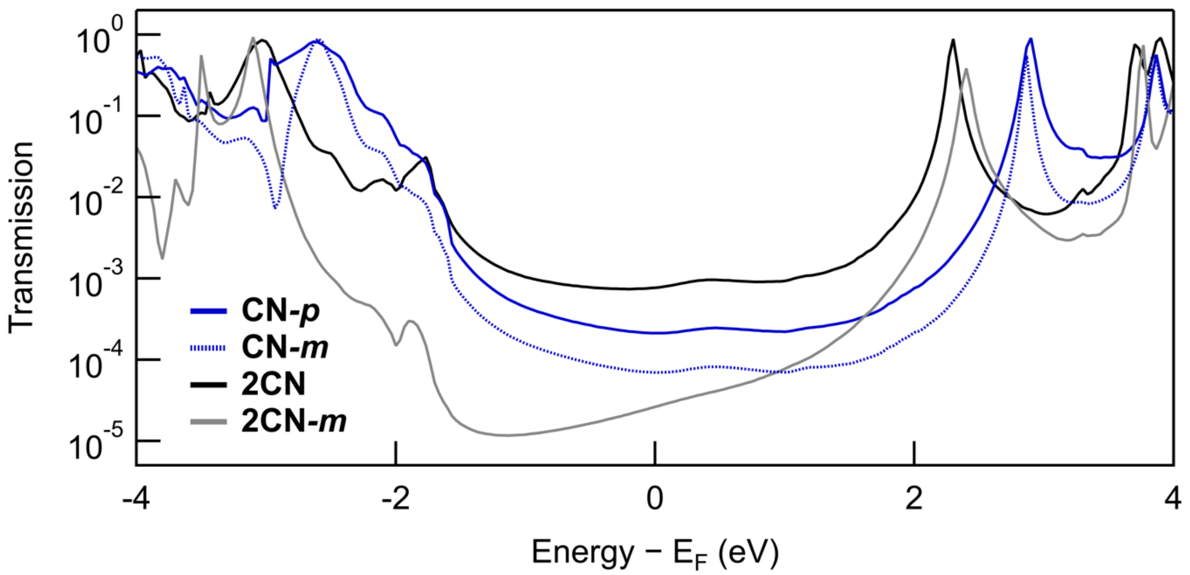

Figure S12. Overlaid transmission functions for **CN-*p***, **CN-*m***, **2CN**, and **2CN-*m***, calculated using DFT+Σ.[25,28] Molecular structures are provided in **Figure 3a**. The expected antiresonances for **CN-*m*** and **2CN-*m*** are observed at around −3 and −4 eV, respectively.[46–48]

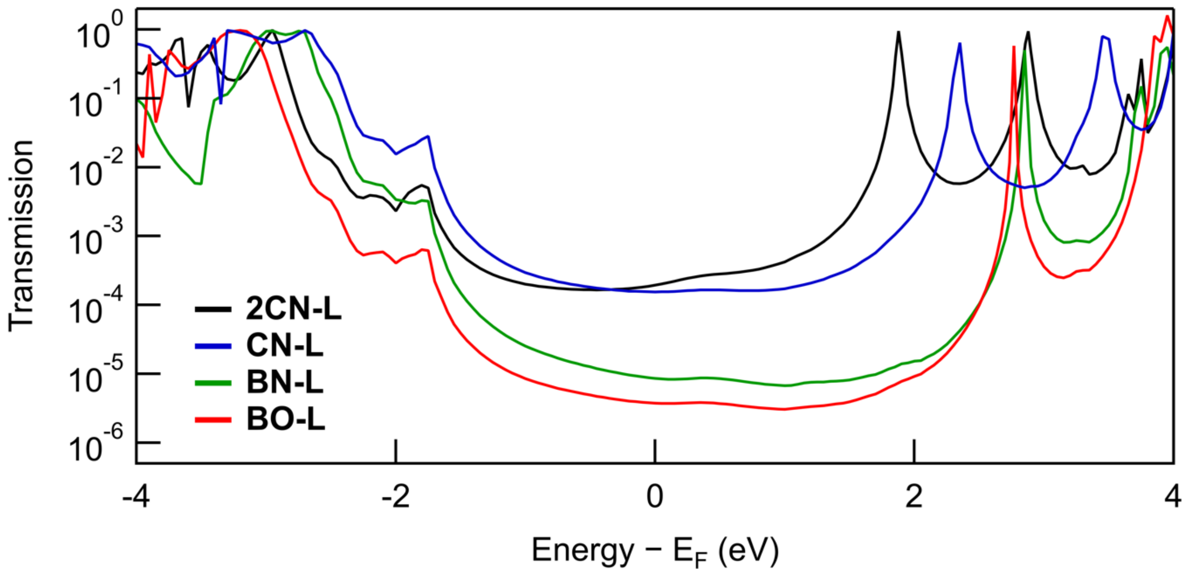

Figure S13. Overlaid transmission functions for the long molecules: **2CN-L**, **CN-L**, **BN-L**, and **BO-L**, calculated using DFT+Σ.[25,28] Molecular structures are provided in **Figure 4a**. The calculated conductance values are plotted in **Figure 4d**.

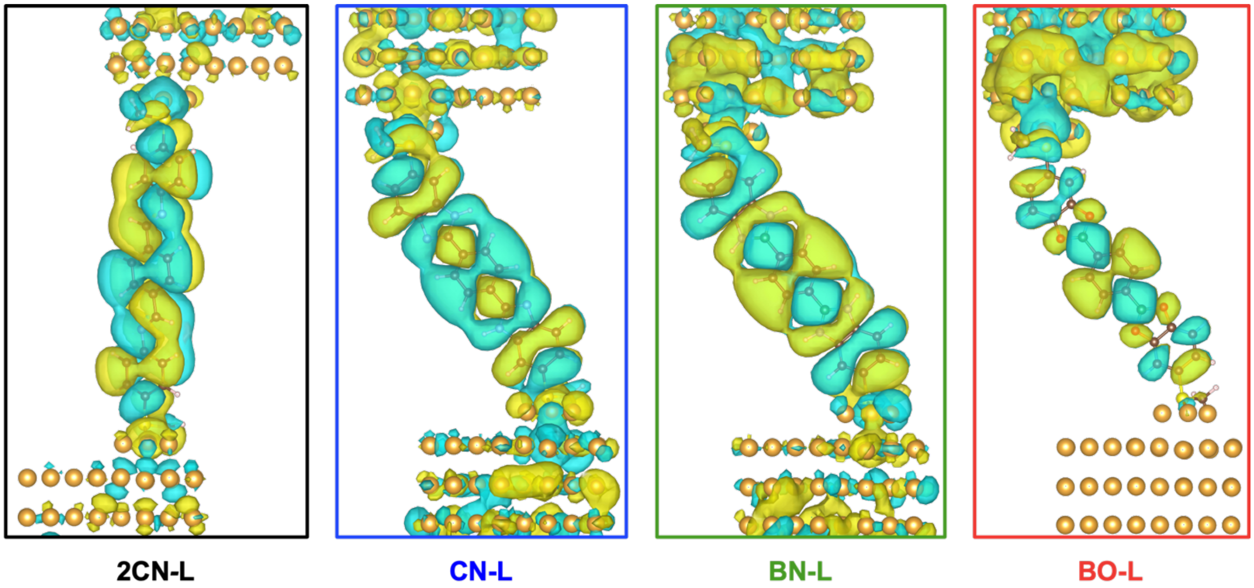

Figure S14. Transmission eigenchannels for molecules with two DCC bridge groups, evaluated at the LUMO resonance peaks. The LUMO resonance is symmetric for all molecules, and the resonance for **BO-L** shows distinct charge localization near the oxygen atoms (as also observed for **BO**). Molecular structures are provided in **Figure 4a**, with transmission functions shown in **Figure S13**.

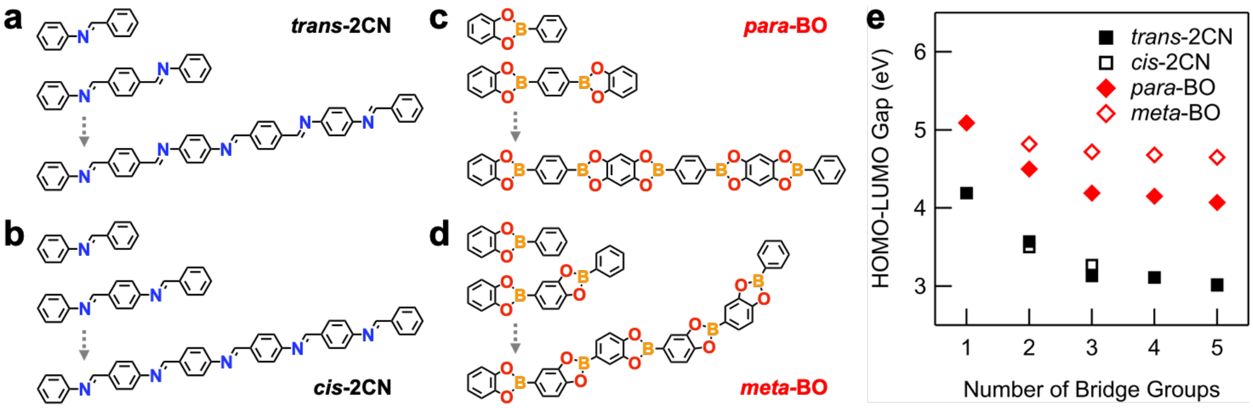

Figure S15. Evaluating changes in HOMO-LUMO gaps for oligomers containing imine (2CN) or boronate ester (BO) bridge groups with different lengths and connectivity. Molecular structures are provided in panels **a-d**. As shown in panel **e**, the calculated HOMO-LUMO gaps are smaller for oligomers containing *trans*-2CN groups, and exhibit a larger decrease upon length extension compared to *para*-BO (1.18 vs 1.02 eV). While only minor differences are observed between HOMO-LUMO gaps for *trans*- and *cis*-2CN oligomers, the gap decrease for *meta*-BO oligomers is different to *para*-BO and the smallest of all oligomers studied. We attribute the distinct properties of *meta*-BO to the *meta*-connectivity in these oligomers which limits orbital delocalization.

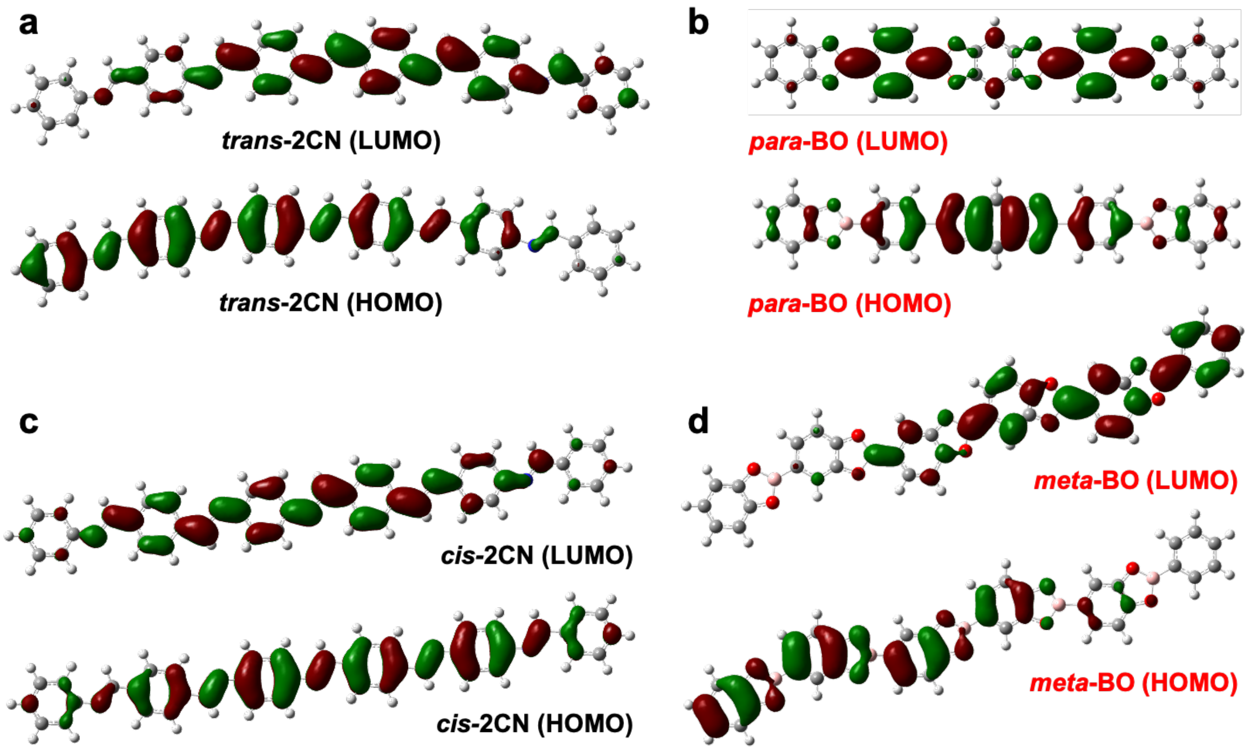

Figure S16. Isosurface plots of frontier orbitals for the longest oligomers shown in **Figure S15** as illustrative examples. The HOMO and LUMO for 2CN oligomers extend over 3-5 bridge groups without interruption. Nodes on atoms limit orbital delocalization across ~3 or fewer groups for BO oligomers, most significantly for *meta*-BO.

Table S2. Calculated frontier orbital energies for the oligomers shown in **Figure S15**.

oligomer	*# of bridge groups*	*HOMO (eV)*	*LUMO (eV)*	*HOMO-LUMO Gap (eV)*
trans-2CN	1	−6.27	−2.08	4.19
	2	−6.13	−2.56	3.57
	3	−5.87	−2.74	3.13
	4	−5.93	−2.82	3.11
	5	−5.87	−2.86	3.01
cis-2CN	1	−6.27	−2.08	4.19
	2	−6.03	−2.53	3.50
	3	−5.95	−2.68	3.27
	4	−5.90	−2.79	3.11
	5	−5.88	−2.86	3.02
para-BO	1	−6.50	−1.41	5.09
	2	−6.46	−1.96	4.50
	3	−6.19	−1.99	4.19
	4	−6.22	−2.07	4.15
	5	−6.16	−2.08	4.07
meta-BO	1	−6.50	−1.41	5.09
	2	−6.39	−1.56	4.82
	3	−6.35	−1.63	4.72
	4	−6.34	−1.66	4.68
	5	−6.33	−1.68	4.65

5. NMR Spectra

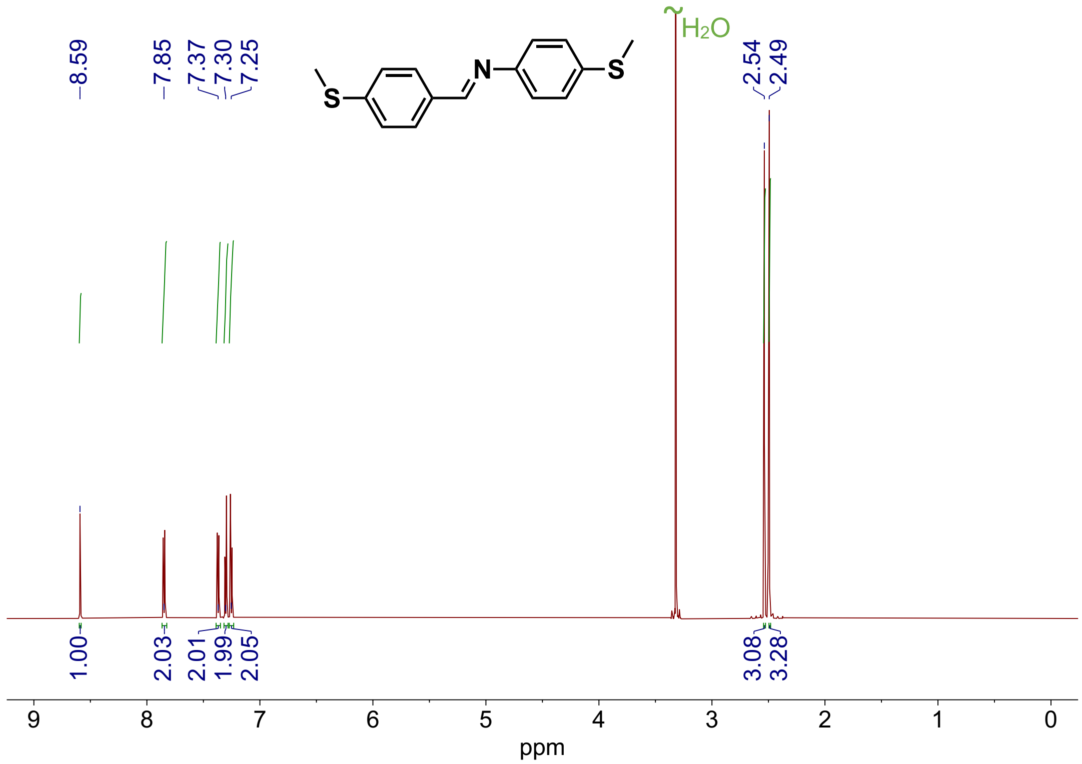

Figure S17. ^{1}H NMR (600 MHz) spectrum of **2CN** in DMSO-d_6.

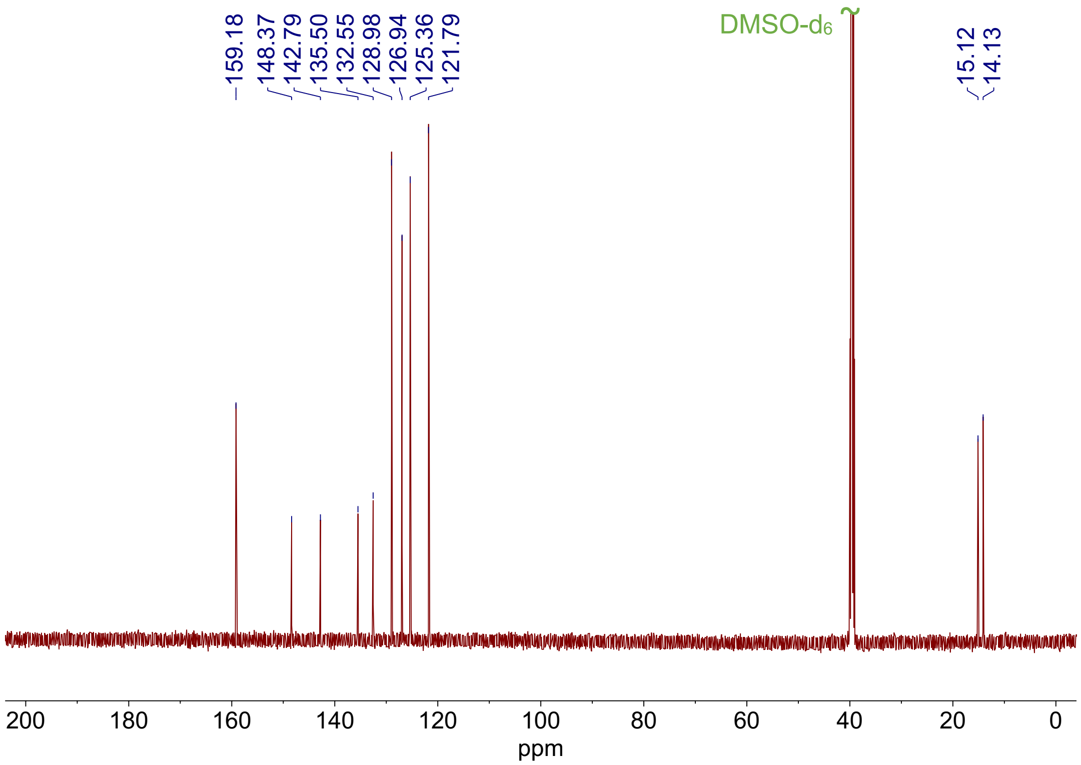

Figure S18. $^{13}C\{^{1}H\}$ NMR (150 MHz) spectrum of **2CN** in DMSO-d_6.

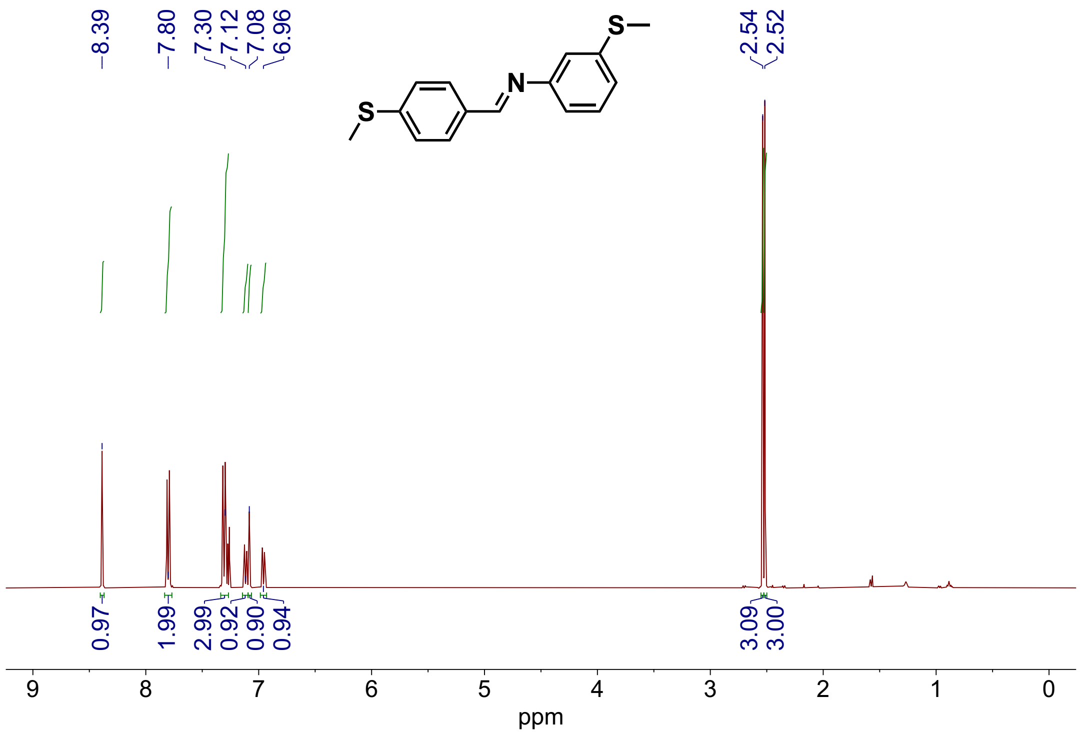

Figure S19. ^{1}H NMR (400 MHz) spectrum of **2CN-m** in $CDCl_3$.

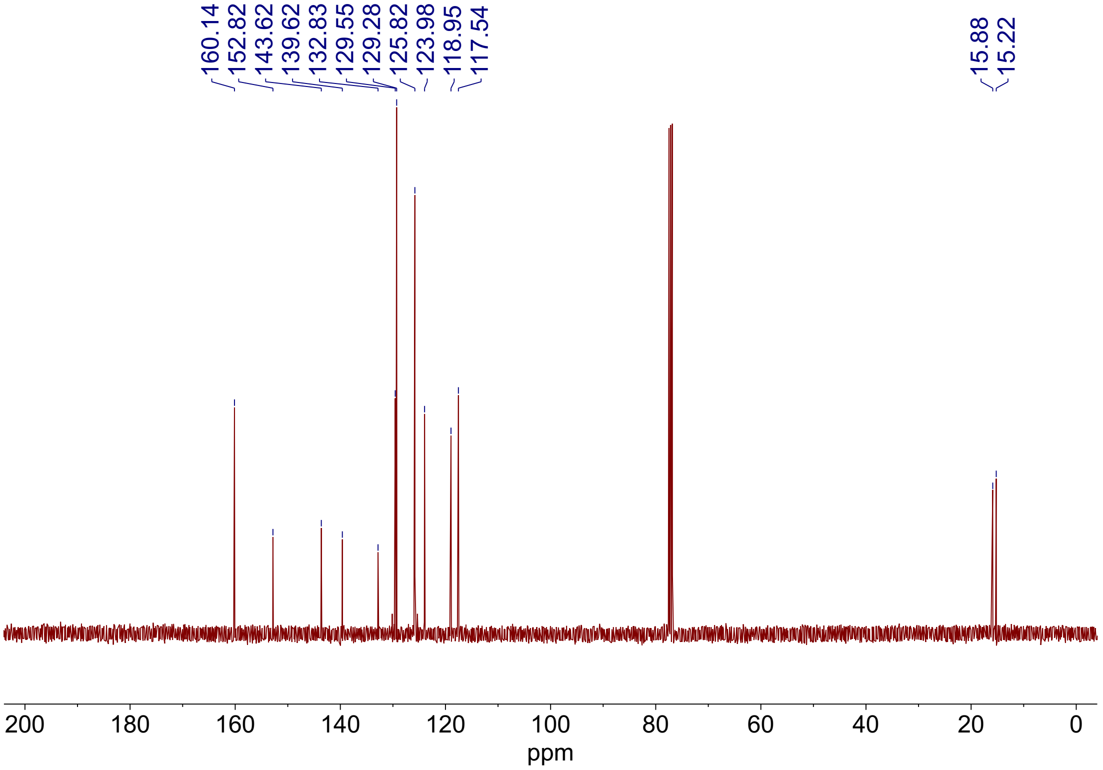

Figure S20. $^{13}C\{^{1}H\}$ NMR (100 MHz) spectrum of **2CN-m** in $CDCl_3$.

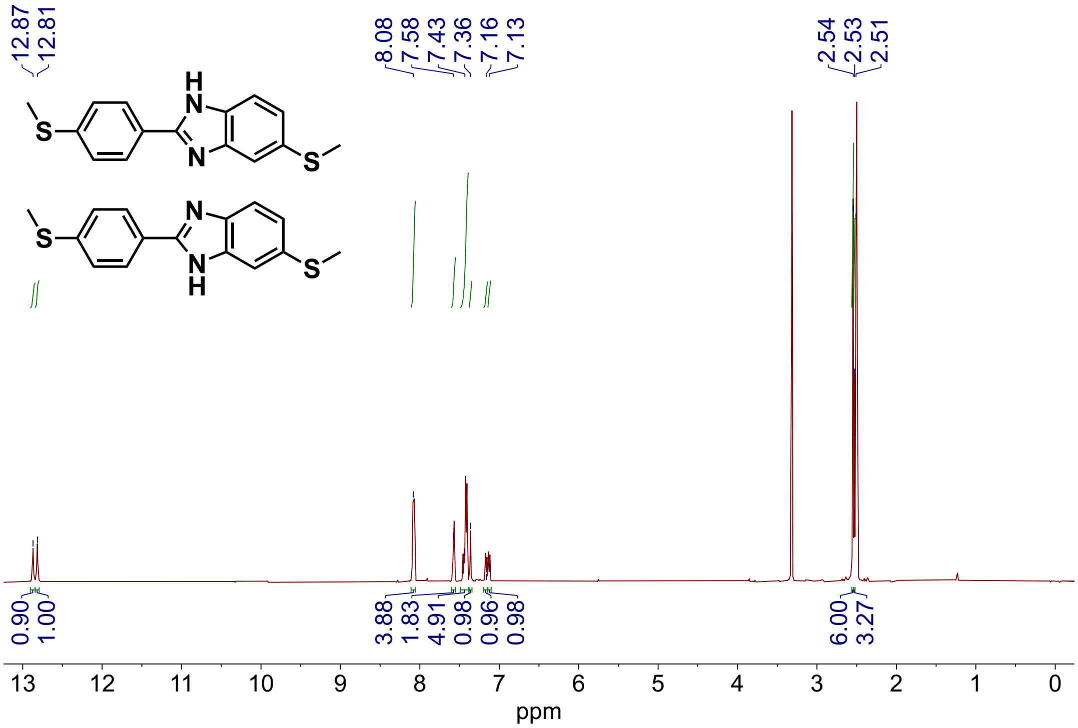

Figure S21. ^{1}H NMR (500 MHz) spectrum of **CN** (interconverting tautomers) in DMSO-d_6

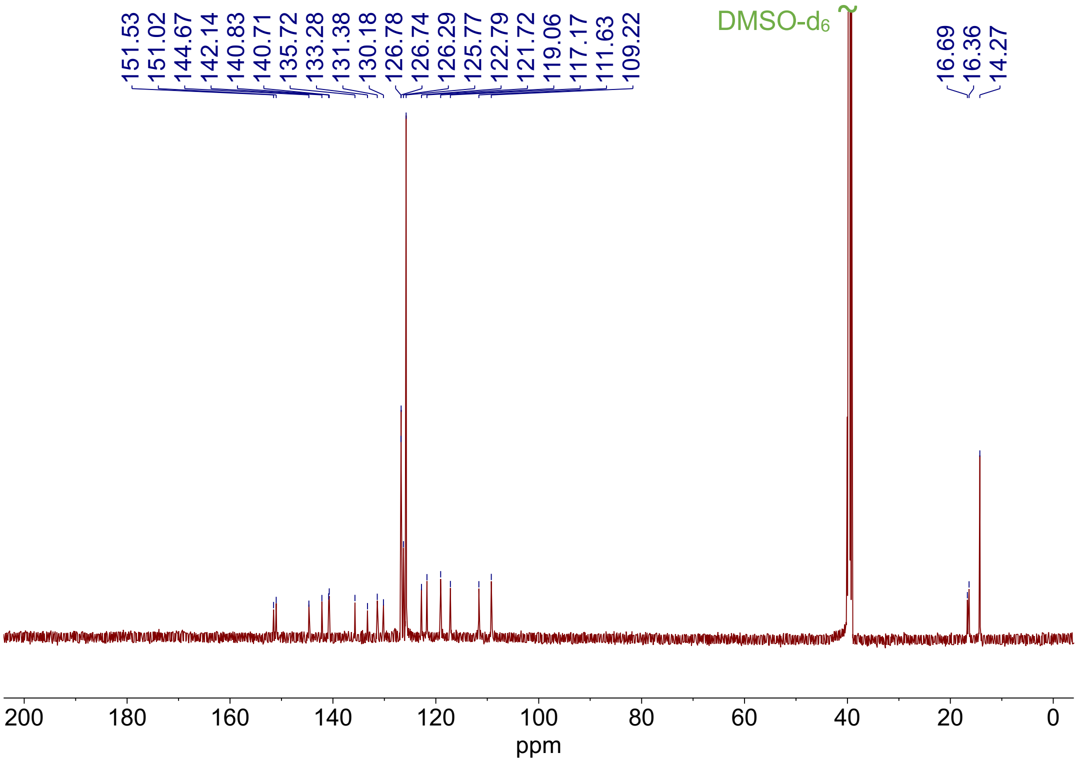

Figure S22. ^{13}C{^{1}H} NMR (125 MHz) spectrum of **CN** (interconverting tautomers) in DMSO-d_6.

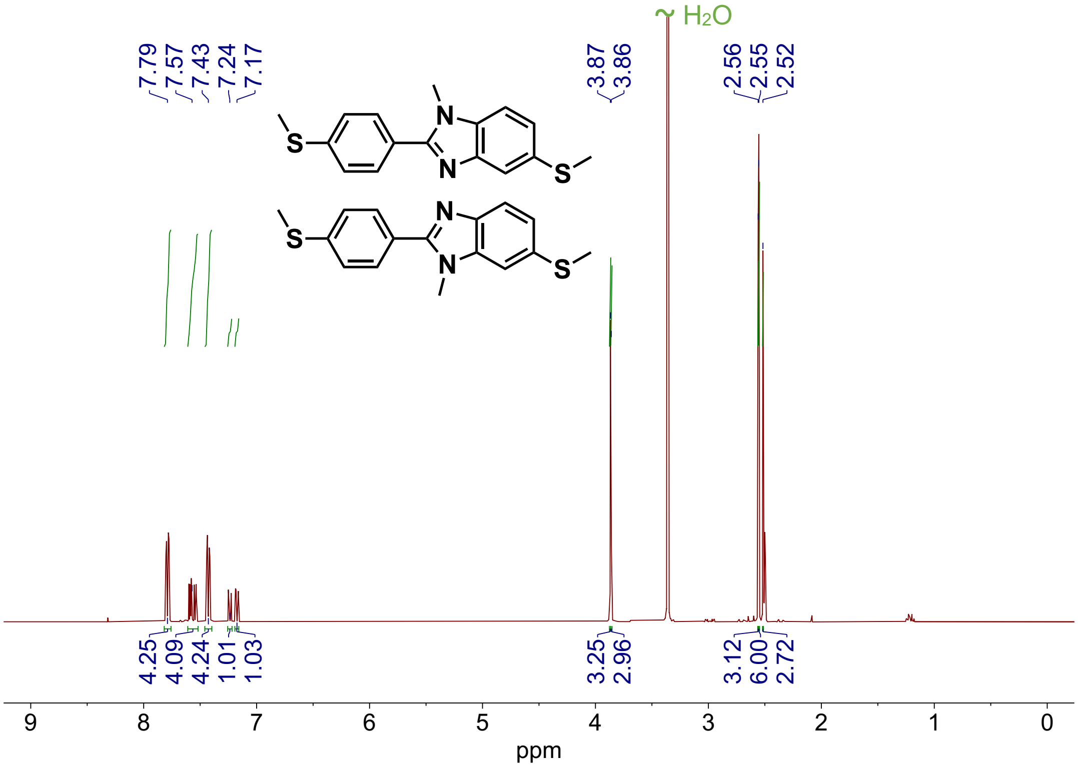

Figure S23. ^{1}H NMR (400 MHz) spectrum of **CN-Me** (isomeric mixture) in DMSO-d_6.

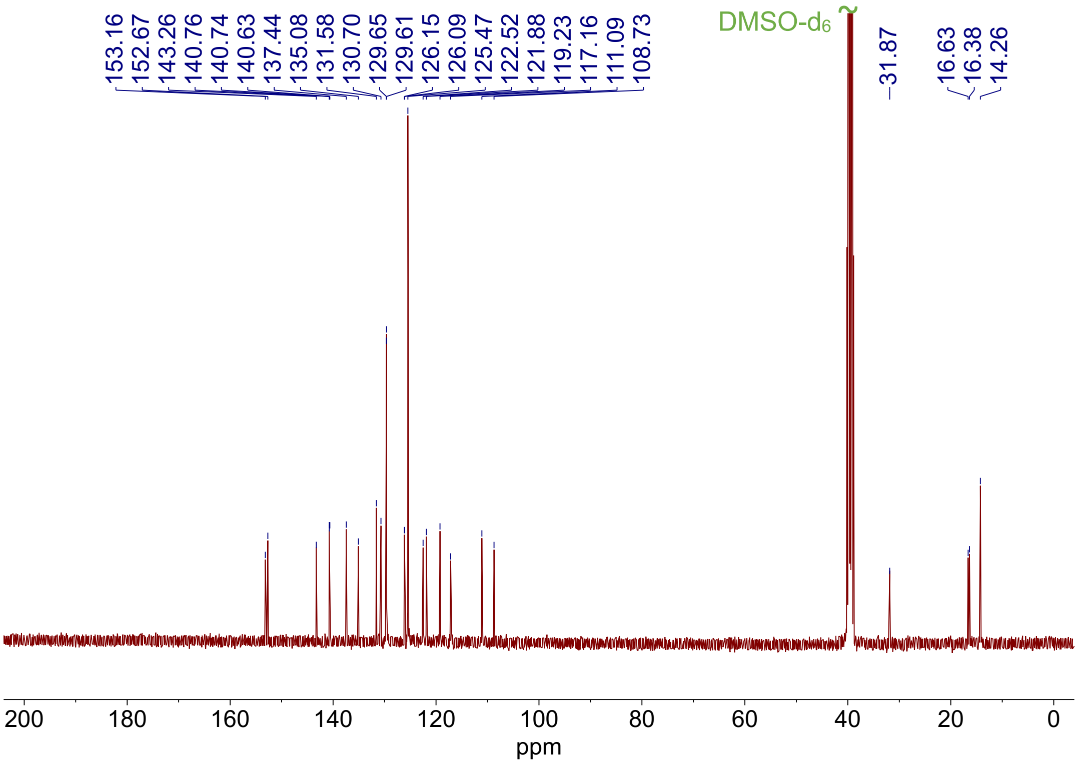

Figure S24. $^{13}C\{^{1}H\}$ NMR (100 MHz) spectrum of **CN-Me** (isomeric mixture) in DMSO-d_6.

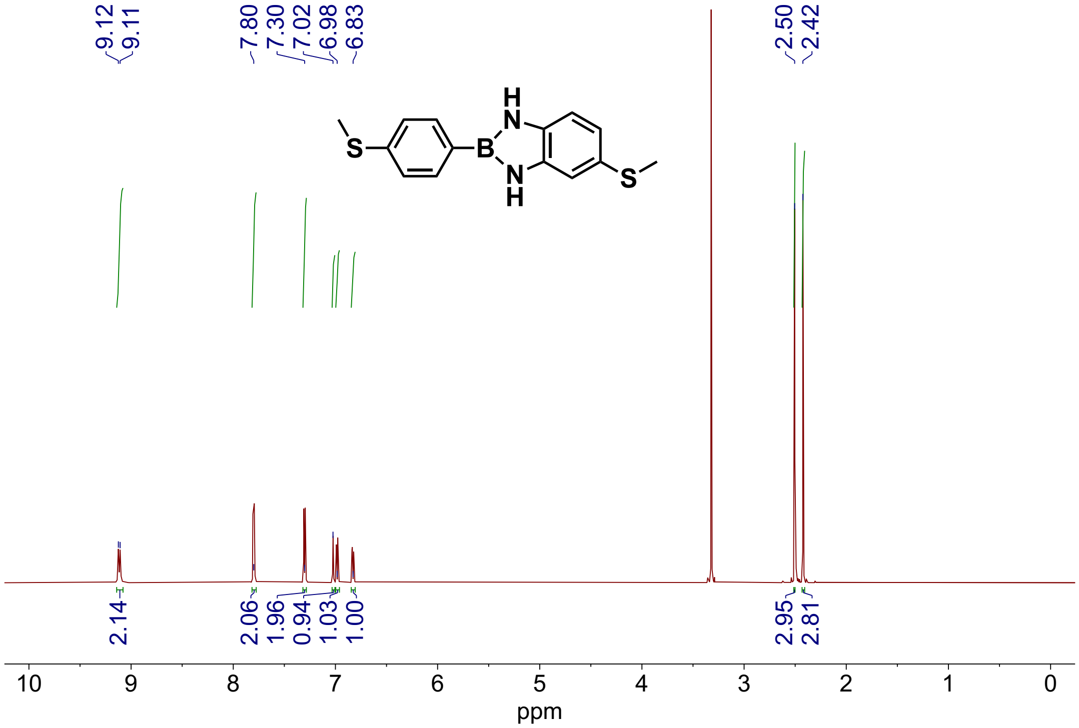

Figure S25. ^{1}H NMR (600 MHz) spectrum of **BN** in DMSO-d_6. ^{1}H NMR stability studies in toluene-d_8 are shown in **Figure S38**.

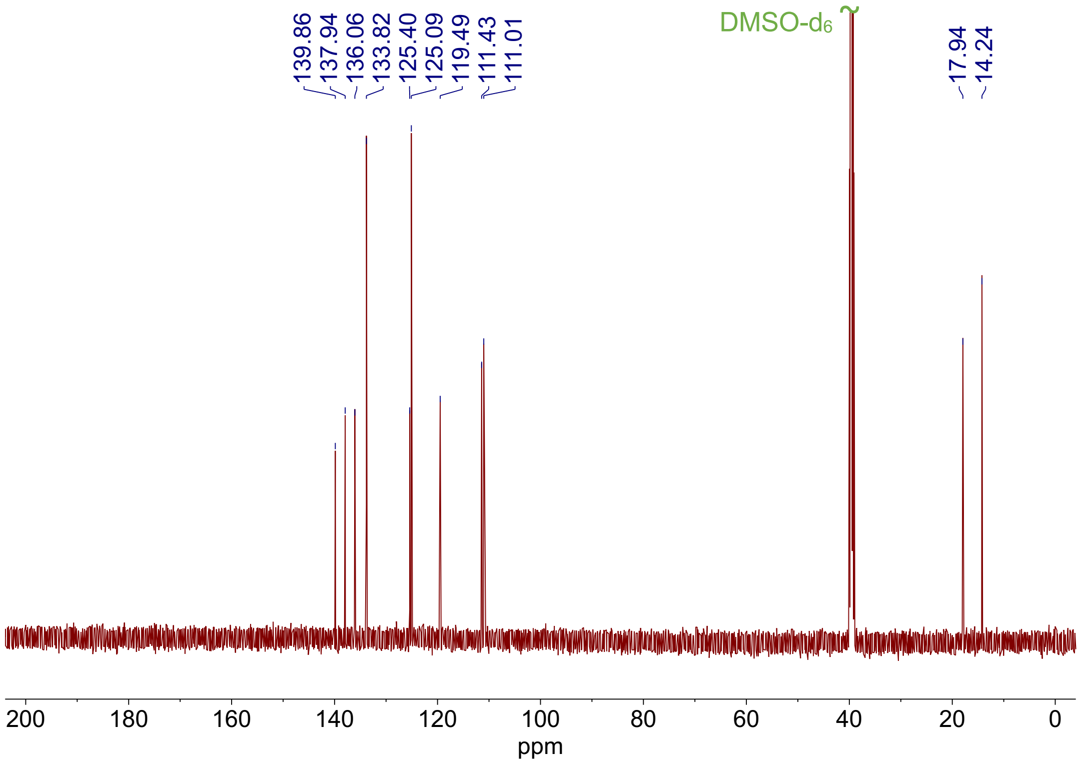

Figure S26. ^{13}C{^{1}H} NMR (150 MHz) spectrum of **BN** in DMSO-d_6.

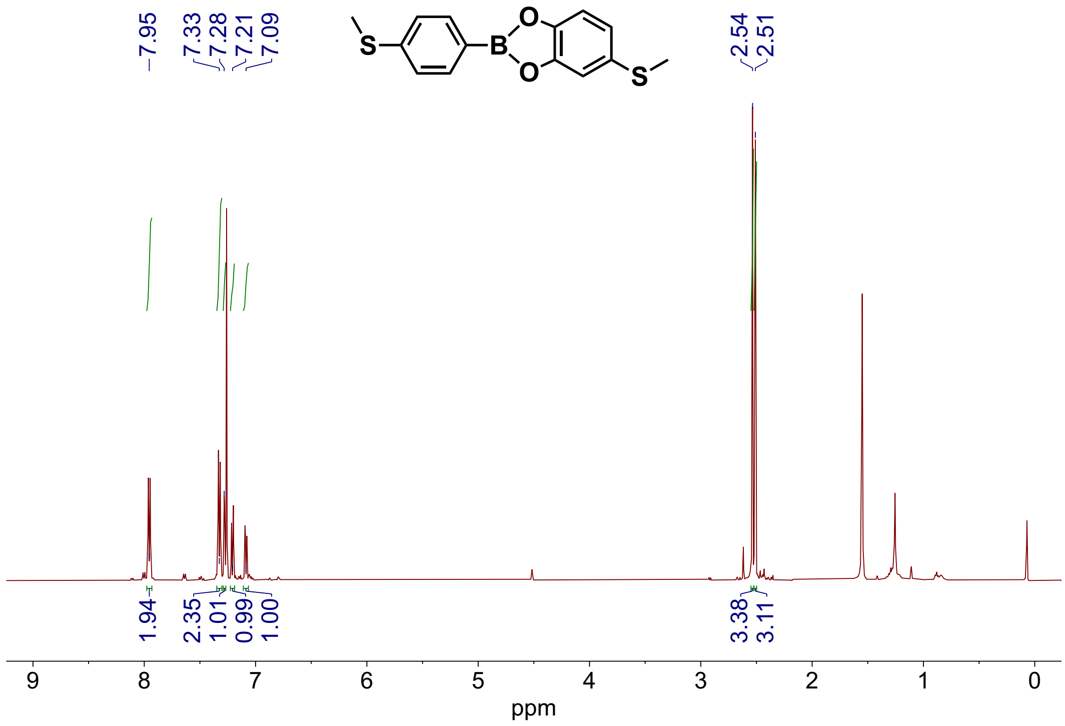

Figure S27. ^{1}H NMR (500 MHz) spectrum of **BO** in $CDCl_3$. ^{1}H NMR stability studies in toluene-d_8 are shown in **Figure S38**.

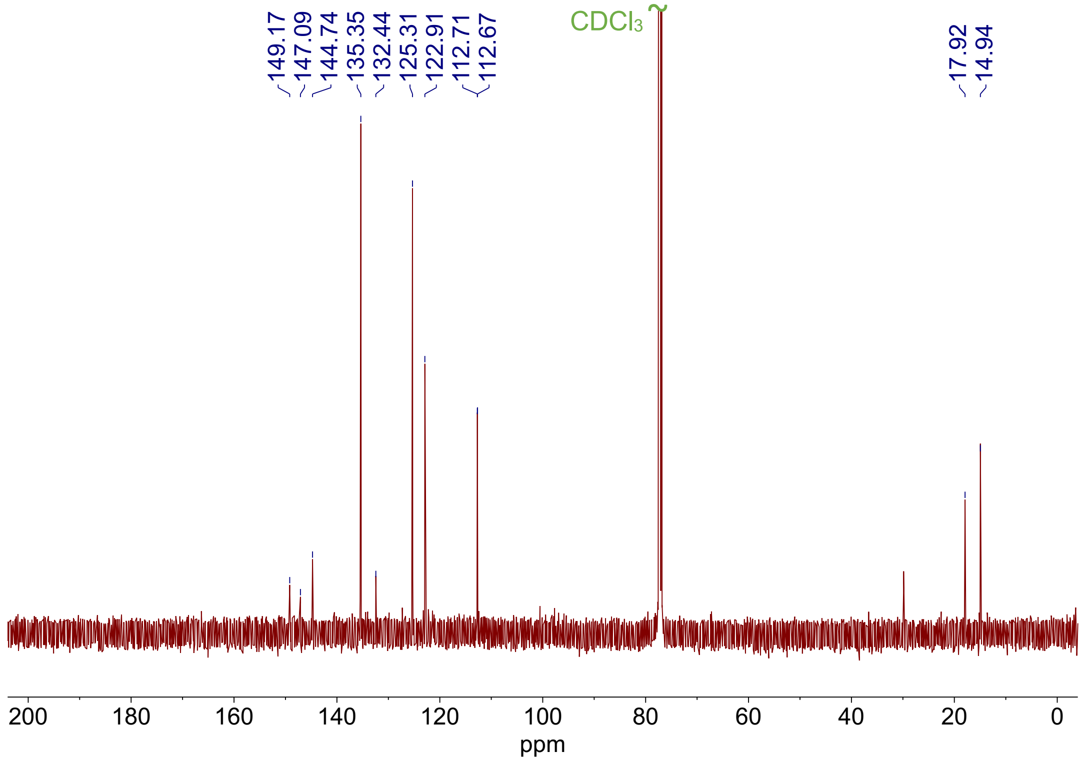

Figure S28. $^{13}C\{^{1}H\}$ NMR (125 MHz) spectrum of **BO** in $CDCl_3$.

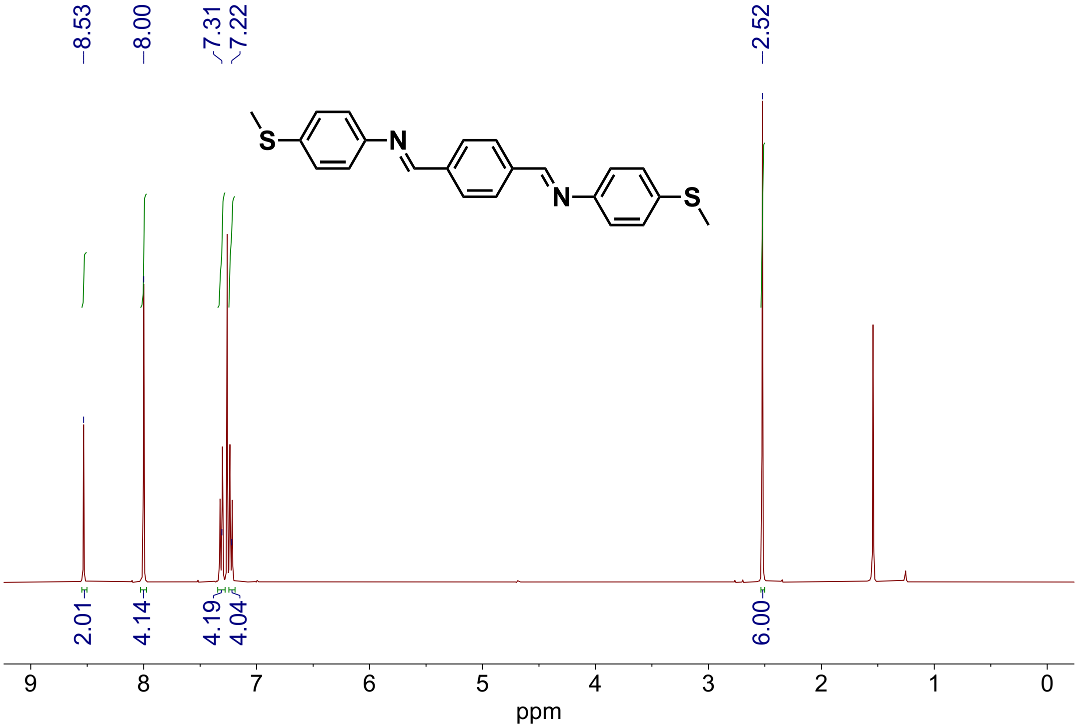

Figure S29. ^{1}H NMR (400 MHz) spectrum of **2CN-L** in $CDCl_3$.

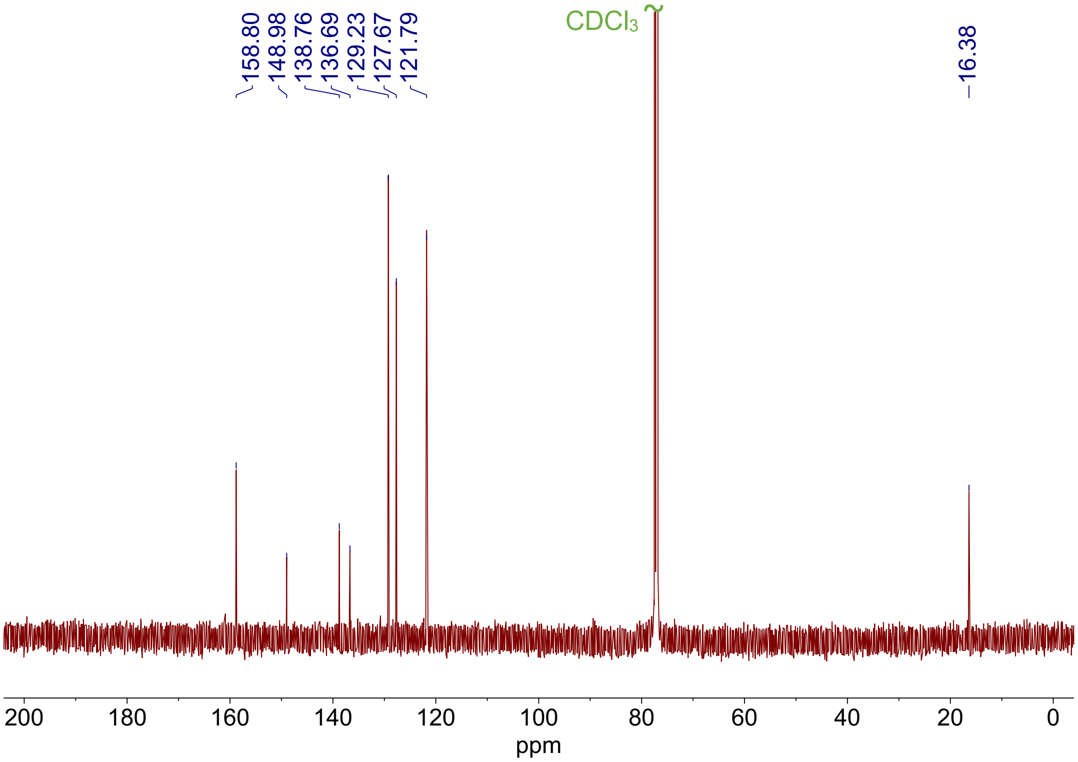

Figure S30. $^{13}C\{^{1}H\}$ NMR (125 MHz) spectrum of **2CN-L** in $CDCl_3$.

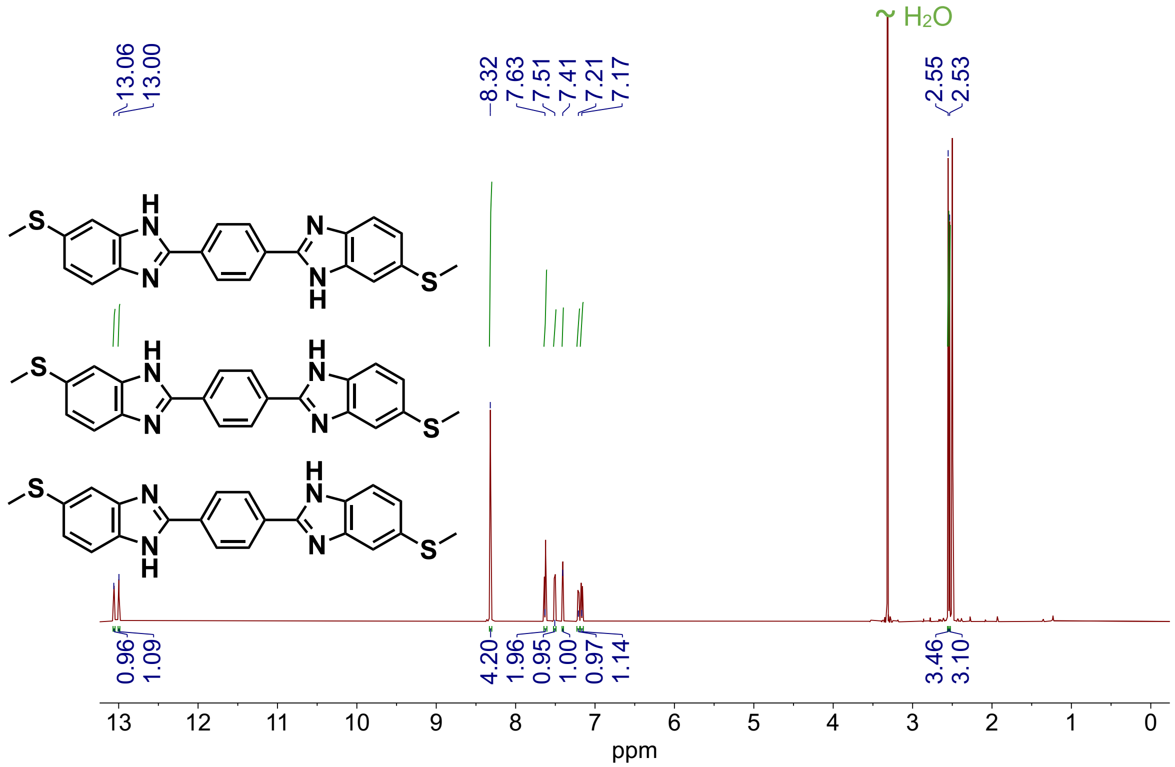

Figure S31. ^{1}H NMR (600 MHz) spectrum of **CN-L** (interconverting tautomers) in DMSO-d_6.

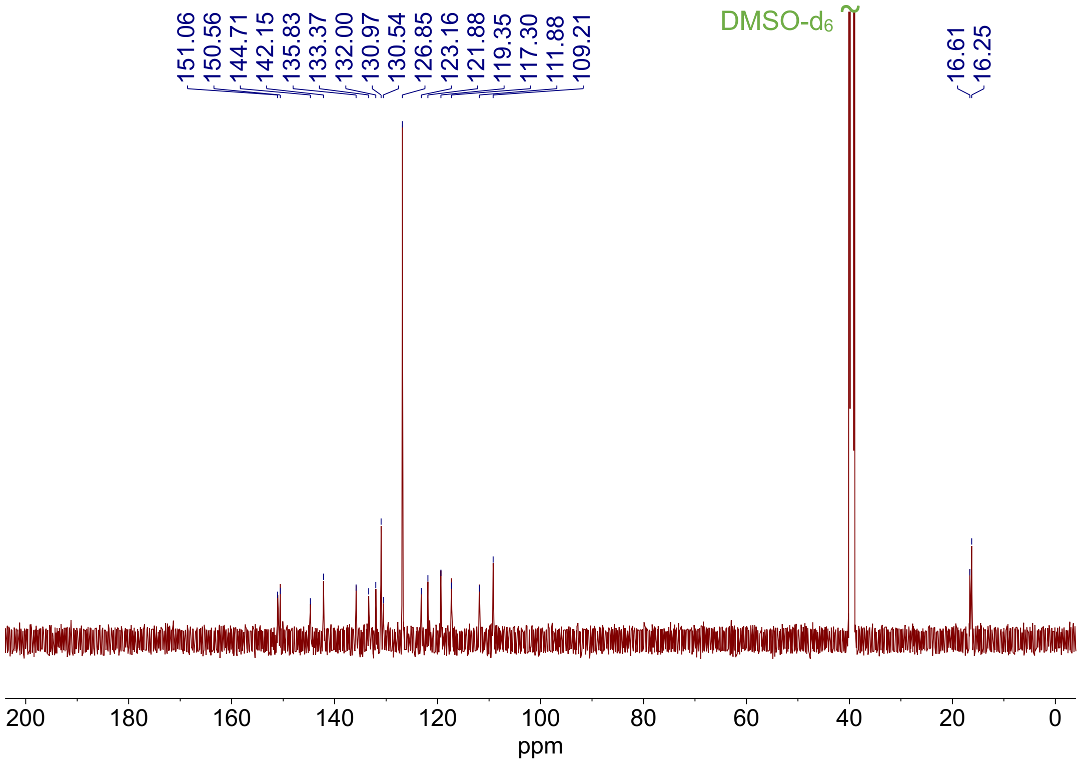

Figure S32. $^{13}C\{^{1}H\}$ NMR (150 MHz) spectrum of **CN-L** (interconverting tautomers) in DMSO-d_6.

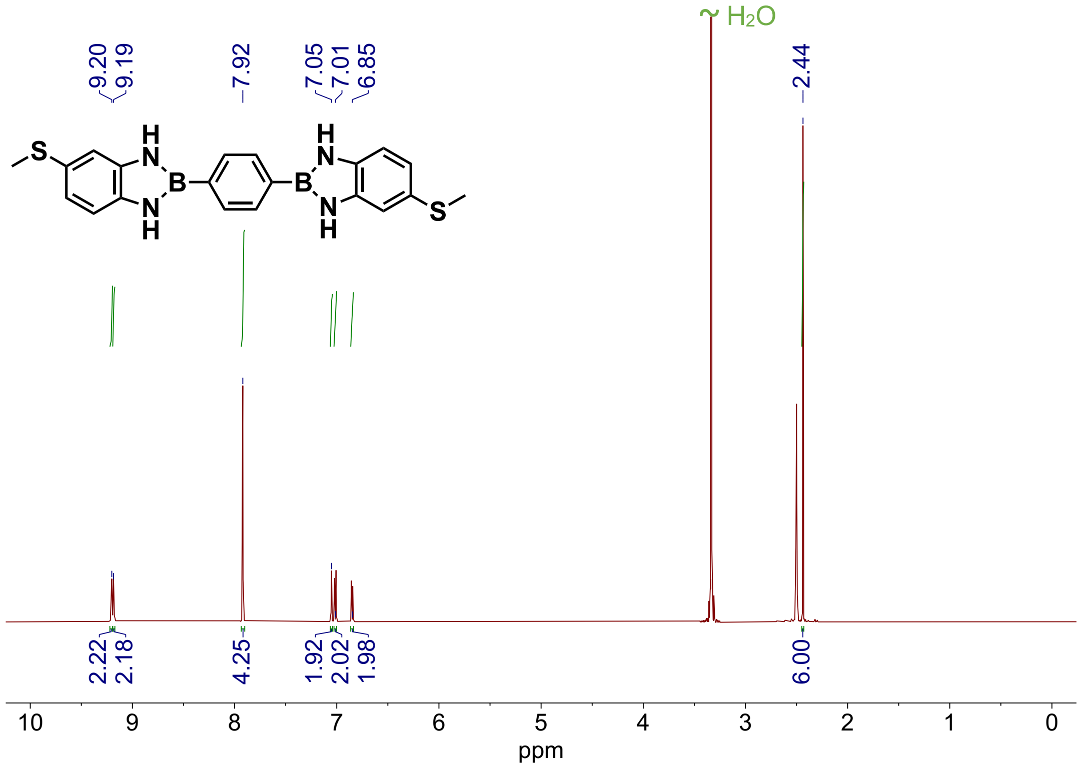

Figure S33. ^{1}H NMR (600 MHz) spectrum of **BN-L** in DMSO-d_6.

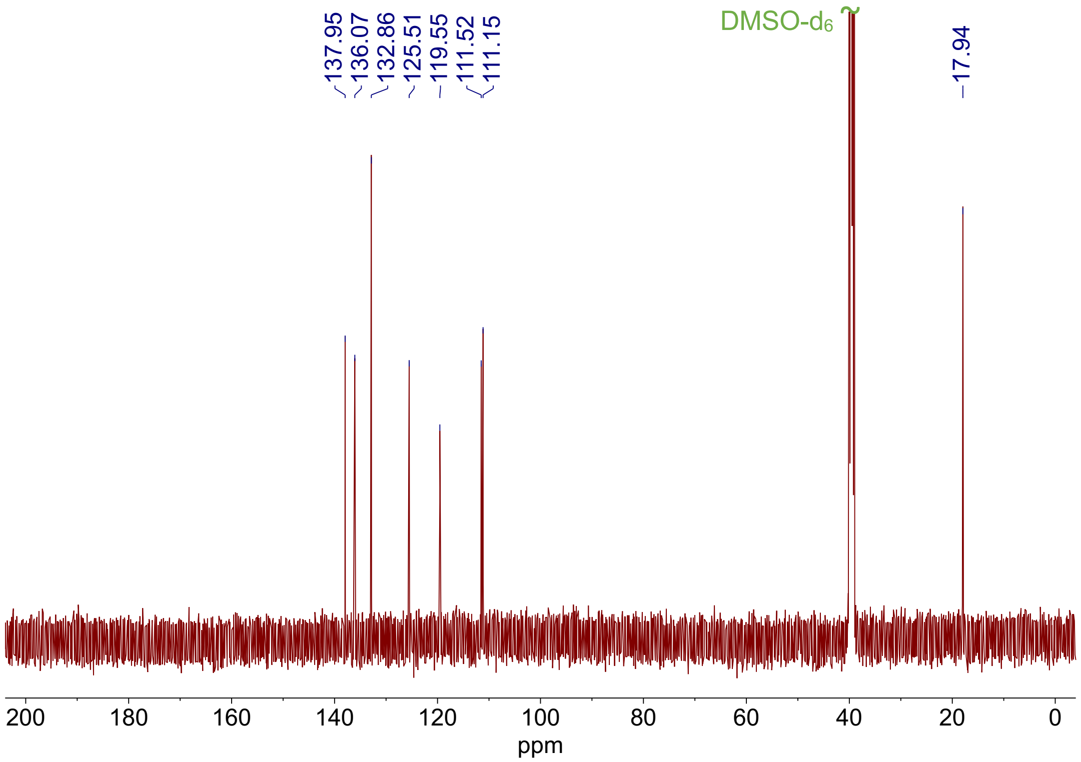

Figure S34. $^{13}C\{^{1}H\}$ NMR (150 MHz) spectrum of **BN-L** in DMSO-d_6.

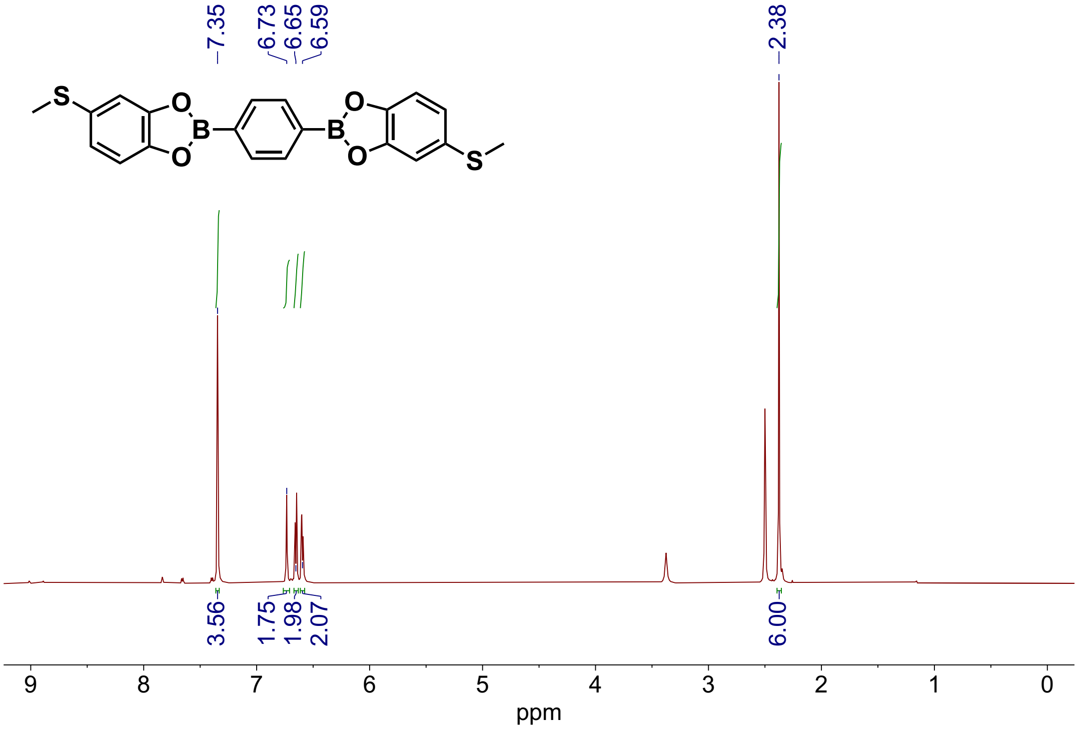

Figure S35. ^{1}H NMR (600 MHz) spectrum of **BO-L** in DMSO-d_6.

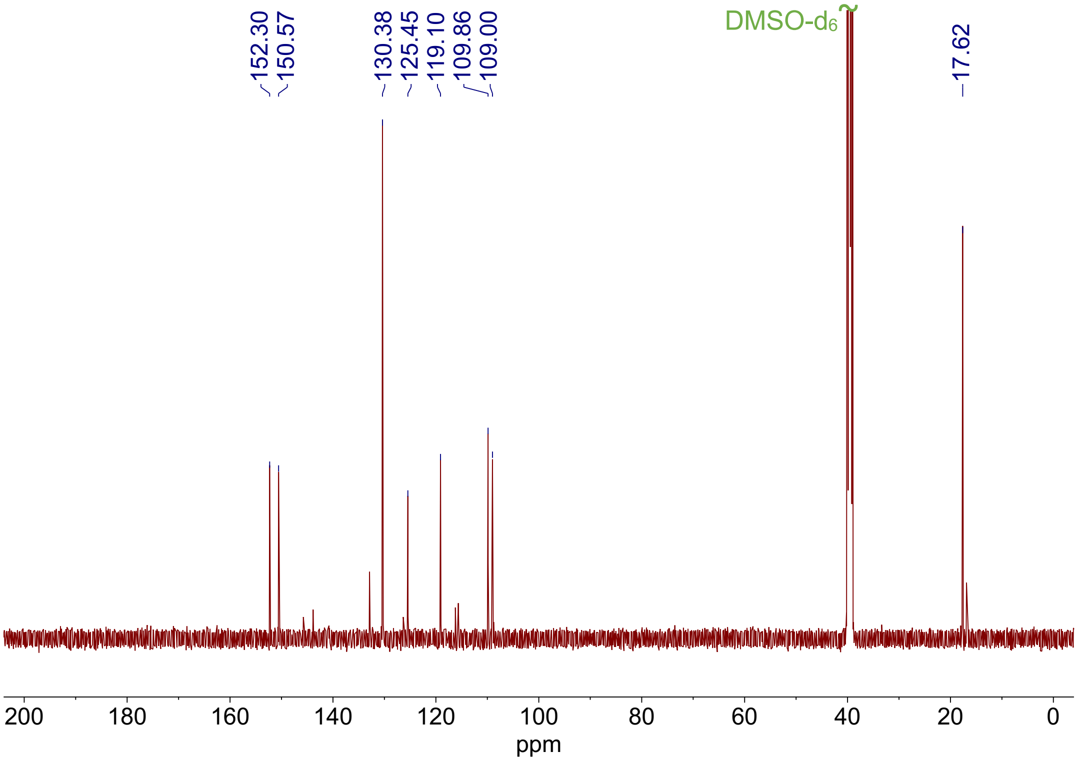

Figure S36. $^{13}C\{^{1}H\}$ NMR (150 MHz) spectrum of **BO-L** in DMSO-d_6.

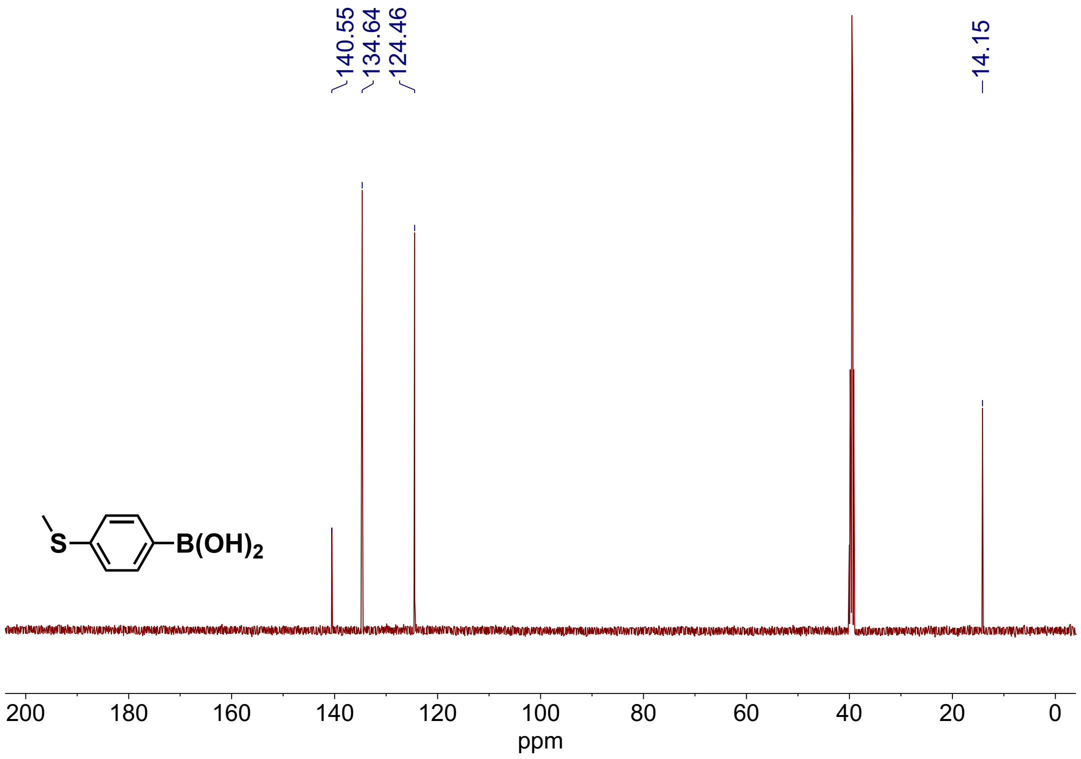

Figure S37. $^{13}C\{^{1}H\}$ NMR (125 MHz) spectrum of **MeS-BO** in DMSO-d_6, showing only three of the four expected aromatic resonances. We attribute the absent resonance to C_{ipso}-B, broadened by coupling to ^{11}B (spin = 3/2) and ^{10}B (spin = 3) nuclei.[49] Such resonances have previously been reported missing in $^{13}C\{^{1}H\}$ spectra of related compounds.[50]

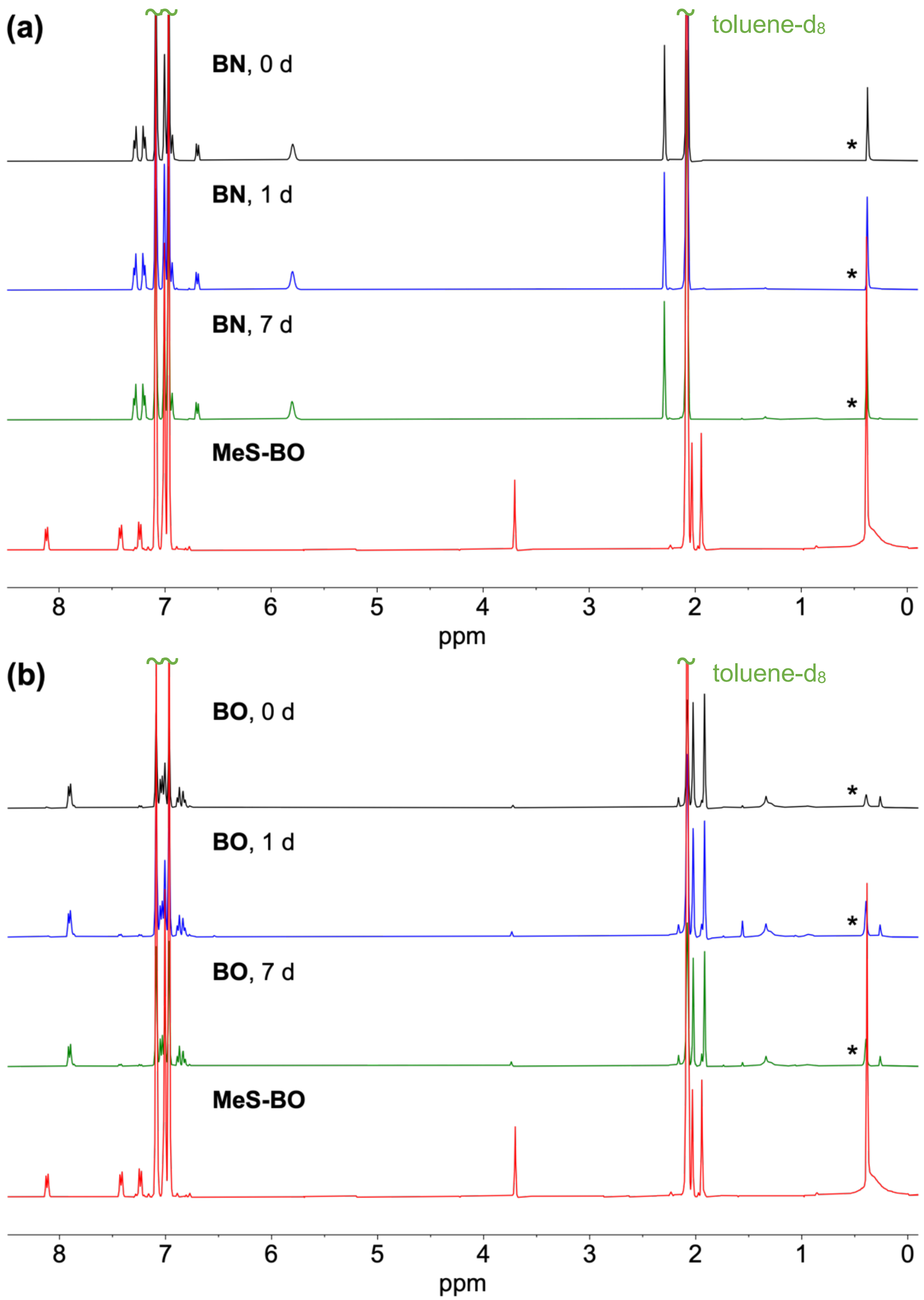

Figure S38. ^1H NMR (400 MHz) spectra of **(a) BN** and **(b) BO** measured at different time internals over 1 week in toluene-d_8 (a chemically comparable aromatic solvent), to probe their hydrolytic stability during STM-BJ experiments in TCB solutions. NMR tubes were uncapped in-between measurements to maximize air exposure. The spectrum for **MeS-BO** (a common hydrolysis product) is provided in each panel for comparison. Resonances attributable to **MeS-BO** are absent, or do not appreciably increase in intensity (where present as minor impurities), in the **BN** and **BO** solution samples. This shows these compounds do not significantly hydrolyze in solution after 1 week even in the presence of adventitious water (starred peaks at $\boldsymbol{\delta}$ ~0.43 ppm).

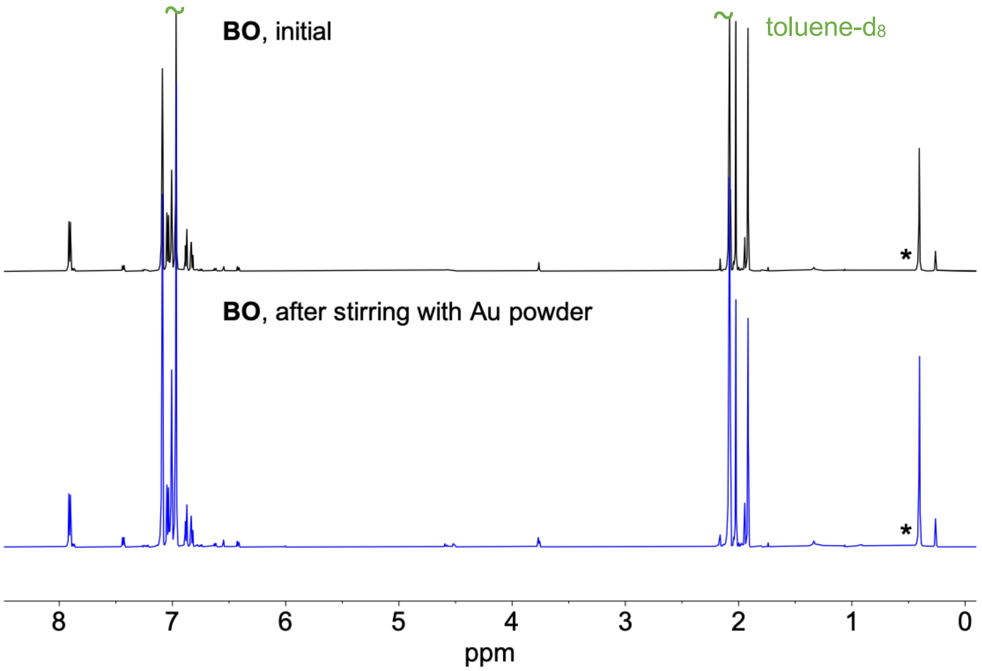

Figure S39. ^{1}H NMR (600 MHz) spectra for a sample of **BO** in ~0.6 mL toluene-d_8 measured before (top) and after (bottom) stirring open to air for ~2 h with 0.05 g Au powder (<10 µm diameter) in the presence of adventitious water (starred peaks at $\boldsymbol{\delta}$ ~0.43 ppm).[42,51] Prior to spectroscopic characterization, the stirred solution sample was passed through a sand-cotton wool filter to remove Au powder. No appreciable spectral changes are observed, indicating that significant molecular dissociation of the solute to soluble, stable products does not occur upon contact with a gold surface area of approximately the same order of magnitude as present in STM-BJ studies, on relevant measurement timescales (**Figure S4**).

6. References

(1) Williams, D. B. G.; Lawton, M. Drying of Organic Solvents: Quantitative Evaluation of the Efficiency of Several Desiccants. *J. Org. Chem.* **2010**, *75*, 8351–8354.

(2) Fulmer, G. R.; Miller, A. J. M.; Sherden, N. H.; Gottlieb, H. E.; Nudelman, A.; Stoltz, B. M.; Bercaw, J. E.; Goldberg, K. I. NMR Chemical Shifts of Trace Impurities: Common Laboratory Solvents, Organics, and Gases in Deuterated Solvents Relevant to the Organometallic Chemist. *Organometallics* **2010**, *29*, 2176–2179.

(3) Venkataraman, L.; Klare, J. E.; Tam, I. W.; Nuckolls, C.; Hybertsen, M. S.; Steigerwald, M. L. Single-Molecule Circuits with Well-Defined Molecular Conductance. *Nano Lett.* **2006**, *6*, 458–462.

(4) Inkpen, M. S.; Liu, Z.; Li, H.; Campos, L. M.; Neaton, J. B.; Venkataraman, L. Non-Chemisorbed Gold–Sulfur Binding Prevails in Self-Assembled Monolayers. *Nature. Chem.* **2019**, *11*, 351–358.

(5) Nagahara, L. A.; Thundat, T.; Lindsay, S. M. Preparation and Characterization of STM Tips for Electrochemical Studies. *Rev. Sci. Instrum.* **1989**, *60*, 3128–3130.

(6) Li, X.; Xu, B.; Xiao, X.; Yang, X.; Zang, L.; Tao, N. Controlling Charge Transport in Single Molecules Using Electrochemical Gate. *Faraday Discuss.* **2006**, *131*, 111–120.

(7) Pobelov, I. V; Li, Z.; Wandlowski, T. Electrolyte Gating in Redox-Active Tunneling Junctions—An Electrochemical STM Approach. *J. Am. Chem. Soc.* **2008**, *130*, 16045–16054.

(8) Li, X.-M.; Wang, Y.-H.; Seng, J.-W.; Zheng, J.-F.; Cao, R.; Shao, Y.; Chen, J.-Z.; Li, J.-F.; Zhou, X.-S.; Mao, B.-W. Z-Piezo Pulse-Modulated STM Break Junction: Toward Single-Molecule Rectifiers with Dissimilar Metal Electrodes. *ACS Appl. Mater. Interfaces* **2021**, *13*, 8656–8663.

(9) Deng, D. R.; Yuan, R. M.; Yu, P. K.; Xue, F.; Fan, X. X.; Lei, J.; Zhang, J. L.; Lin, X. D.; Wu, Q. H.; Fan, J. M.; Chang, J. K.; Hong, W. J.; Zheng, M. Sen; Dong, Q. F. An Enhanced Electrode via Coupling with a Conducting Molecule to Extend Interfacial Reactions. *Adv. Energy Mater.* **2021**, *11*, 1–10.

(10) Xiang, L.; Zhang, P.; Liu, C.; He, X.; Li, H. B.; Li, Y.; Wang, Z.; Hihath, J.; Kim, S. H.; Beratan, D. N.; Tao, N. Conductance and Configuration of Molecular Gold-Water-Gold Junctions under Electric Fields. *Matter* **2020**, *3*, 166–179.

(11) Kim, T. Silver Electrodes for Reversible Oxygen Sensor Applications. *J. Korean Phys. Soc.* **2015**, *67*, 823–826.

(12) Li, J.; Pudar, S.; Yu, H.; Li, S.; Moore, J. S.; Rodríguez-López, J.; Jackson, N. E.; Schroeder, C. M. Reversible Switching of Molecular Conductance in Viologens Is Controlled by the Electrochemical Environment. *J. Phys. Chem. C* **2021**, *125*, 21862–21872.

(13) Chen, H.; Jiang, F.; Hu, C.; Jiao, Y.; Chen, S.; Qiu, Y.; Zhou, P.; Zhang, L.; Cai, K.; Song, B.; Chen, X.-Y.; Zhao, X.; Wasielewski, M. R.; Guo, H.; Hong, W.; Stoddart, J. F. Electron-Catalyzed Dehydrogenation in a Single-Molecule Junction. *J. Am. Chem. Soc.* **2021**, *143*, 8476–8487.

(14) Schneebeli, S. T.; Kamenetska, M.; Cheng, Z.; Skouta, R.; Friesner, R. A.; Venkataraman, L.; Breslow, R. Single-Molecule Conductance through Multiple π-π-Stacked Benzene Rings Determined with Direct Electrode-to-Benzene Ring Connections. *J. Am. Chem. Soc.* **2011**, *133*, 2136–2139.

(15) Kay, N. J.; Higgins, S. J.; Jeppesen, J. O.; Leary, E.; Lycoops, J.; Ulstrup, J.; Nichols, R. J. Single-Molecule Electrochemical Gating in Ionic Liquids. *J. Am. Chem. Soc.* **2012**, *134*, 16817–16826.

(16) Wierzbinski, E.; Yin, X.; Werling, K.; Waldeck, D. H. The Effect of Oxygen Heteroatoms on the Single Molecule Conductance of Saturated Chains. *J. Phys. Chem. B* **2013**, *117*, 4431–4441.

(17) Capozzi, B.; Dell, E. J.; Berkelbach, T. C.; Reichman, D. R.; Venkataraman, L.; Campos, L. M. Length-Dependent Conductance of Oligothiophenes. *J. Am. Chem. Soc.* **2014**, *136*, 10486–10492.

(18) Li, H.; Su, T. A.; Camarasa-Gómez, M.; Hernangómez-Pérez, D.; Henn, S. E.; Pokorný, V.; Caniglia, C. D.; Inkpen, M. S.; Korytár, R.; Steigerwald, M. L.; Nuckolls, C.; Evers, F.; Venkataraman, L. Silver Makes Better Electrical Contacts to Thiol-Terminated Silanes than Gold. *Angew. Chem. Int. Ed.* **2017**, *56*, 14145–14148.

(19) Li, Y.; Buerkle, M.; Li, G.; Rostamian, A.; Wang, H.; Wang, Z.; Bowler, D. R.; Miyazaki, T.; Xiang, L.; Asai, Y.; Zhou, G.; Tao, N. Gate Controlling of Quantum Interference and Direct Observation of Anti-Resonances in Single Molecule Charge Transport. *Nature Mater.* **2019**, *18*, 357–363.

(20) Meng, M.; Tang, Z.; Mallick, S.; Luo, M. H.; Tan, Z.; Liu, J. Y.; Shi, J.; Yang, Y.; Liu, C. Y.; Hong, W. Enhanced Charge Transport via d(δ)-p(π) Conjugation in Mo2-Integrated Single-Molecule Junctions. *Nanoscale* **2020**, *12*, 10320–10327.

(21) Chen, H.; Brasiliense, V.; Mo, J.; Zhang, L.; Jiao, Y.; Chen, Z.; Jones, L. O.; He, G.; Guo, Q.-H.; Chen, X.-Y.; Song, B.; Schatz, G. C.; Stoddart, J. F. Single-Molecule Charge Transport through Positively Charged Electrostatic Anchors. *J. Am. Chem. Soc.* **2021**, *143*, 2886–2895.

(22) Liu, Z.-F.; Wei, S.; Yoon, H.; Adak, O.; Ponce, I.; Jiang, Y.; Jang, W.-D.; Campos, L. M.; Venkataraman, L.; Neaton, J. B. Control of Single-Molecule Junction Conductance of Porphyrins via a Transition-Metal Center. *Nano Lett.* **2014**, *14*, 5365–5370.

(23) Soler, J. M.; Artacho, E.; Gale, J. D.; García, A.; Junquera, J.; Ordejón, P.; Sánchez-Portal, D. The SIESTA Method for Ab Initio Order-N Materials Simulation. *J. Phys. Condens. Matter* **2002**, *14*, 2745–2779.

(24) Perdew, J. P.; Burke, K.; Ernzerhof, M. Generalized Gradient Approximation Made Simple. *Phys. Rev. Lett.* **1996**, *77*, 3865–3868.

(25) Quek, S. Y.; Venkataraman, L.; Choi, H. J.; Louie, S. G.; Hybertsen, M. S.; Neaton, J. B. Amine−Gold Linked Single-Molecule Circuits: Experiment and Theory. *Nano Lett.* **2007**, *7*, 3477–3482.

(26) Brandbyge, M.; Mozos, J.-L.; Ordejón, P.; Taylor, J.; Stokbro, K. Density-Functional Method for Nonequilibrium Electron Transport. *Phys. Rev. B* **2002**, *65*, 165401.

(27) Papior, N.; Lorente, N.; Frederiksen, T.; García, A.; Brandbyge, M. Improvements on Non-Equilibrium and Transport Green Function Techniques: The next-Generation TRANSIESTA. *Comput. Phys. Commun.* **2017**, *212*, 8–24.

(28) Neaton, J. B.; Hybertsen, M. S.; Louie, S. G. Renormalization of Molecular Electronic Levels at Metal-Molecule Interfaces. *Phys. Rev. Lett.* **2006**, *97*, 216405.

(29) Kronik, L.; Stein, T.; Refaely-Abramson, S.; Baer, R. Excitation Gaps of Finite-Sized Systems from Optimally Tuned Range-Separated Hybrid Functionals. *J. Chem. Theory Comput.* **2012**, *8*, 1515–1531.

(30) Frisch, M. J.; Trucks, G. W.; Schlegel, H. B.; Scuseria, G. E.; Robb, M. A.; Cheeseman, J. R.; Scalmani, G.; Barone, V.; Petersson, G. A.; Nakatsuji, H.; Li, X.; Caricato, M.; Marenich, A. V.; Bloino, J.; Janesko, B. G.; Gomperts, R.; Mennucci, B.; Hratchian, H.

P.; Ortiz, J. V.; Izmaylov, A. F.; Sonnenberg, J. L.; Williams-Young, D.; Ding, F.; Lipparini, F.; Egidi, F.; Goings, J.; Peng, B.; Petrone, A.; Henderson, T.; Ranasinghe, D.; Zakrzewski, V. G.; Gao, J.; Rega, N.; Zheng, G.; Liang, W.; Hada, M.; Ehara, M.; Toyota, K.; Fukuda, R.; Hasegawa, J.; Ishida, M.; Nakajima, T.; Honda, Y.; Kitao, O.; Nakai, H.; Vreven, T.; Throssell, K.; Montgomery, J. A., J.; Peralta, J. E.; Ogliaro, F.; Bearpark, M. J.; Heyd, J. J.; Brothers, E. N.; Kudin, K. N.; Staroverov, V. N.; Keith, T. A.; Kobayashi, R.; Normand, J.; Raghavachari, K.; Rendell, A. P.; Burant, J. C.; Iyengar, S. S.; Tomasi, J.; Cossi, M.; Millam, J. M.; Klene, M.; Adamo, C.; Cammi, R.; Ochterski, J. W.; Martin, R. L.; Morokuma, K.; Farkas, O.; Foresman, J. B.; Fox, D. J. Gaussian 16,Revision C.01. Gaussian, Inc: Wallingford CT 2016.

(31) Kuntz, K.; Emmitte, K. A.; Rheault, T. R.; Smith, S.; Hornberger, K.; Dickson, H.; Cheung, M. Benzimidazole Thiophene Compounds and Their Preparation, Pharmaceutical Compositions and Use in the Treatment of Diseases. US 0300247, 2008.

(32) Nouch, R.; Cini, M.; Magre, M.; Abid, M.; Diéguez, M.; Pàmies, O.; Woodward, S.; Lewis, W. Enantioselective Synthesis of 6,6-Disubstituted Pentafulvenes Containing a Chiral Pendant Hydroxy Group. *Chem. Eur. J.* **2017**, *23*, 17195–17198.

(33) Kabalka, G. W.; Reddy, N. K.; Narayana, C. Sodium Percarbonate: A Convenient Reagent for the Dakin Reaction. *Tetrahedron Lett.* **1992**, *33*, 865–866.

(34) Cooksey, C. J.; Land, E. J.; Riley, P. A. A Simple One-Pot Preparation of 4-Alkoxy- and 4-Alkylthio-Catechols and o-Benzoquinones. *Org. Prep. Proced. Int.* **1996**, *28*, 463–498.

(35) Robert, J.-M.; Troy-Fioramonti, S.; Demizieux, L.; Degrace, P. Preparation of 1,4-Bis(4-Methylthiophenyl)-3-Phthaloylazetidin-2-One and Derivatives Thereof Useful for Treatment of Diseases Assocd. with Hyperactivity of the Endocannabinoid System. US 0265498, 2018.

(36) Escala, N.; Valderas-García, E.; Bardón, M. Á.; Gómez de Agüero, V. C.; Escarcena, R.; López-Pérez, J. L.; Rojo-Vázquez, F. A.; San Feliciano, A.; Balaña-Fouce, R.; Martínez-Valladares, M.; del Olmo, E. Synthesis, Bioevaluation and Docking Studies of Some 2-Phenyl-1H-Benzimidazole Derivatives as Anthelminthic Agents against the Nematode Teladorsagia Circumcincta. *Eur. J. Med. Chem.* **2020**, *208*, 112554.

(37) Hong, X.; Wang, H.; Liu, B.; Xu, B. Ruthenium-Catalyzed Double-Fold C-H Tertiary Alkoxycarbonylation of Arenes Using Di-Tert-Butyl Dicarbonate. *Chem. Commun.* **2014**,

50, 14129–14132.
(38) Slabber, C. A.; Grimmer, C. D.; Robinson, R. S. Solution-State 15N NMR and Solid-State Single-Crystal XRD Study of Heterosubstituted Diazaboroles and Borinines Prepared via an Effective and Simple Microwave-Assisted Solvent-Free Synthesis. *J. Organomet. Chem.* **2013**, *723*, 122–128.
(39) Smith, M. K.; Northrop, B. H. Vibrational Properties of Boroxine Anhydride and Boronate Ester Materials: Model Systems for the Diagnostic Characterization of Covalent Organic Frameworks. *Chem. Mater.* **2014**, *26*, 3781–3795.
(40) Savino, C.; Ryan, R. P.; Knee, J. L.; Jimenez-Hoyos, C. A.; Northrop, B. H. Electronic Spectroscopy of 2-Phenyl-1,3,2-Benzodioxaborole and Its Derivatives: Important Building Blocks of Covalent Organic Frameworks. *J. Phys. Chem. A* **2020**, *124*, 529–537.
(41) Zang, Y.; Stone, I.; Inkpen, M. S.; Ng, F.; Lambert, T. H.; Nuckolls, C.; Steigerwald, M. L.; Roy, X.; Venkataraman, L. In Situ Coupling of Single Molecules Driven by Gold-Catalyzed Electrooxidation. *Angew. Chem. Int. Ed.* **2019**, *58*, 16008–16012.
(42) Li, Y.; Zhao, C.; Wang, R.; Tang, A.; Hong, W.; Qu, D.; Tian, H.; Li, H. In Situ Monitoring of Transmetallation in Electric Potential-Promoted Oxidative Coupling in a Single-Molecule Junction. *CCS Chem.* **2022**, 1–9.
(43) Thijssen, W. H. A.; Marjenburgh, D.; Bremmer, R. H.; van Ruitenbeek, J. M. Oxygen-Enhanced Atomic Chain Formation. *Phys. Rev. Lett.* **2006**, *96*, 026806.
(44) O'Driscoll, L. J.; Bryce, M. R. Extended Curly Arrow Rules to Rationalise and Predict Structural Effects on Quantum Interference in Molecular Junctions. *Nanoscale* **2021**, *13*, 1103–1123.
(45) Capozzi, B.; Xia, J.; Adak, O.; Dell, E. J.; Liu, Z.-F.; Taylor, J. C.; Neaton, J. B.; Campos, L. M.; Venkataraman, L. Single-Molecule Diodes with High Rectification Ratios through Environmental Control. *Nature Nanotechnol.* **2015**, *10*, 522–527.
(46) Sautet, P.; Joachim, C. Electronic Interference Produced by a Benzene Embedded in a Polyacetylene Chain. *Chem. Phys. Lett.* **1988**, *153*, 511–516.
(47) Mayor, M.; Weber, H. B.; Reichert, J.; Elbing, M.; von Hanisch, C.; Beckmann, D.; Fisher, M. Electric Current through a Molecular Rod - Relevance of the Position of the Anchor Groups. *Angew. Chem. Int. Ed. Engl.* **2003**, *42*, 5834–5838.
(48) Solomon, G. C.; Herrmann, C.; Hansen, T.; Mujica, V.; Ratner, M. A. Exploring Local

Currents in Molecular Junctions. *Nature. Chem.* **2010**, *2*, 223–228.

(49) Wrackmeyer, B. Progress in NMR Spectroscopy, Vol. 12 - Carbon-13 NMR Spectroscopy of Boron Compounds. *Prog. Nucl. Magn. Reson. Spectrosc.* **1979**, *12*, 227–259.

(50) Manankandayalage, C. P.; Unruh, D. K.; Krempner, C. Boronic, Diboronic and Boric Acid Esters of 1,8-Naphthalenediol-Synthesis, Structure and Formation of Boronium Salts. *Dalton Trans.* **2020**, *49*, 4834–4842.

(51) Stone, I. B.; Starr, R. L.; Hoffmann, N.; Wang, X.; Evans, A. M.; Nuckolls, C.; Lambert, T. H.; Steigerwald, M. L.; Berkelbach, T. C.; Roy, X.; Venkataraman, L. Interfacial Electric Fields Catalyze Ullmann Coupling Reactions on Gold Surfaces. *Chem. Sci.* **2022**, 20–24.
